# An MD View of Ligand Binding

**DOI:** 10.3390/molecules30244678

**Published:** 2025-12-06

**Authors:** Adrian Calderon, Eric Harbinson, Rüdiger Ettrich, Natalia Kulik, Jannette Carey

**Affiliations:** 1 Chemistry Department, University of Illinois, Urbana-Champaign, Champaign, IL 61820, USA; 2 Chemistry Department, Rutgers University, New Brunswick, NJ 08901, USA; 3 College of Biomedical Sciences, Larkin University, 18301 N Miami Ave, Miami, FL 33169, USA; 4 Centre Algatech, Institute of Microbiology, Academy of Sciences of the Czech Republic, 37901 Trebon, Czech Republic; 5 Chemistry Department, Princeton University, Princeton, NJ 08544, USA

**Keywords:** glutamyl-queuosine tRNAAsp synthetase, GluQRS, PDB 4A91, affinity, specificity, intrinsic specificity ratio, protein-targeted drug development

## Abstract

Protein–ligand complexes in crystal structures are well described by an array of bonding interactions among precisely defined functional groups. The present work examines how one representative complex behaves in one-microsecond molecular dynamics simulations, starting from a crystal structure with a native biological ligand bound, and proceeding to simulations of structures derived by docking of that native ligand, and then to docking of selected ligand analogs. The MD behaviors and system energies calculated in RMSD plateau regions using MM/GBSA are similar when initiated from the crystal structure or the structure with the docked native ligand, although independent replicate simulations differ. Despite these similarities, interatomic contact frequencies indicate that some contacts observed in the crystal structure are rarely sampled again; others are sampled only intermittently; and new contacts are recruited that can be more persistent. Docked structures of non-native ligand analogs were chosen for simulation by screening manually for features consistent with known binding interactions, and these displayed behaviors similar to those for the native ligand and, in some cases, similar calculated energies. Overall, ligands appear to cooperate dynamically with the protein in forming the observed interactions.

## 1. Introduction

The accurate prediction of protein–ligand binding is often called a grand challenge of computational biology. The statement acknowledges the difficulty of prediction and the small numbers of apparently successful cases. Prediction of a protein–ligand interaction is a first step in many contemporary approaches to protein-targeted drug development. Computational predictions are typically evaluated by comparison with a co-crystalline complex [1]. If the crystal structure differs from the prediction, does that mean the prediction is unsuccessful? This question highlights the fact that our main tools to “see” what binding looks like at the atomic level of detail are crystal or NMR structures and MD simulations, tools that operate at very different ranges of time, resolution, and conditions.

Protein-targeted drug development typically seeks maximal values of affinity and specificity, unlike biological systems, which populate the two-dimensional affinity/specificity space fully [2]. Theoretical, thermodynamic, and experimental considerations lead to certain limitations inherent in the quest to maximize the two values. Affinity, defined as ΔG, the free energy difference between bound and free states, quantifies the difference between two chemical potentials we do not know. A thermodynamically rigorous definition of specificity is the difference in ΔG between one ligand–target pair and another, i.e., ΔΔG. Thus, specificity is not an inherent property of any molecule, but rather is defined only operationally, with a value that depends on which ligand–target pairs are compared. Implicit in this definition is that quantification of specificity for candidate drugs requires comparison with all relevant ligand–target pairs, many of which are unknown or unknowable [3,4].

The free energy difference ΔG further reflects the difference between ΔH and TΔS, the enthalpic and entropic changes in the binding process, typically considered as the change in internal energy (i.e., bonding) and degrees of freedom (i.e., states) in the system, respectively. Like ΔG, the magnitudes of both terms are typically quite small relative to the (unknown) values in the free and bound states. Thus, all three net differences (ΔG, ΔH, TΔS) can vary widely with small changes in any of the many contributing factors in either state of the system. Some contributions can arise from elusive sources including solvent components. For example, the affinity of some protein–DNA interactions can change by orders of magnitude with modest changes in salt concentration, an effect that is not correlated with ionic strength [5]. Rather, it reflects a favorable entropic contribution to the overall free energy change [6] when a protein replaces counterions that partially neutralize the DNA polyelectrolyte surface but are not site-bound [7] and are not detected in crystal structures. The ubiquitous and often-dominant role of hidden contributions to affinity like this one limits the success of efforts to improve binding by improving intermolecular bonding.

Several considerations from experiments are relevant for the drug-design endeavor [8]. The magnitudes of experimental errors in measuring ΔG for ligand binding can be quite large, and are sometimes underestimated by reporting the error of fitting a model to the data, rather than the typically much larger measurement errors that can make small affinity differences meaningless. Random experimental errors can also be amplified by systematic error if the data do not adequately constrain the binding model. A common reason for this failing is if the stoichiometry of binding is treated as an adjustable parameter rather than being determined directly in the same experimental conditions used for measuring affinity. Another consideration is that affinities are sometimes reported in a range of units that may not be directly comparable. A particularly common but troublesome case is the IC_50_ value, which is used incorrectly as a proxy for enzyme/inhibitor affinity. However, IC_50_ represents affinity accurately only for specific kinetic mechanisms that govern the nature of the inhibition [9]. IC_50_ values are typically reported without specifying whether the requisite criterion is met.

Given that affinity and specificity are just numbers, accurate only in specified conditions, and with a humbling degree of error, it is possible that the quest to maximize their values in drug design might be a futile effort. Furthermore, the traditional definition of specificity does not lend itself to the task of maximization, but any alternative definition must be similarly rigorous and unambiguous. One definition has been proposed for use in computational biology that is independent of comparison with other ligand–target interactions. The intrinsic specificity ratio, ISR, relies on the distributions of calculated scores among a population of poses resulting from docking [3,4], or from system energies in MD simulation [10], as in the present work. Qualitatively, the ISR idea is that a preferred (“specific”) pose will reside in a potential energy minimum that is separated by an energy gap from the distribution of poses; as such the need for rigor and clarity is met using statistical thermodynamic principles. This measure sidesteps the definition of specificity somewhat, but nevertheless offers a way to evaluate a group of ligand–target pairs. Rather than quantifying specificity *per se*, ISR quantifies the goodness of fit for potential protein–ligand pairs; as such it sidesteps the issue of affinity as well.

The present work aims to address questions about how we understand affinity and specificity by simulating a protein–ligand complex with a native ligand or analogs. The observed associated states present behaviors that appear unexpected compared with contemporary understanding of the bound state. Some consequences relevant for protein-targeted drug development are discussed.

## 2. Results

Protein GluQRS. Glutamyl-queuosine tRNAAsp synthetase (GluQRS) interacts with its natural ligands, L-glutamic acid (Glu) and ATP, to form glutamyl-AMP, identical to aminoacyl-AMP formed by all synthetases in the first step of protein synthesis. GluQRS was originally chosen as a positive-control protein for a separate project in which random proteins were challenged with random ligands including amino acids. This unusual enzyme has been characterized thoroughly by D. Kern and coworkers, who first identified it as the protein encoded by the *E. coli yadB* gene [11,12] and determined its crystal structure and enzymatic properties [13,14]. GluQRS is a paralog of glutamyl tRNAGlu synthetase (GluRS) that shares only its catalytic aminoacylation domain, and has no equivalent of the anticodon-binding domain.

Unlike GluRS, GluQRS forms glutamyl adenylate (Glu-AMP; structure: Supplementary Figure S1) directly, independently of the binding of tRNA, which for GluRS is a requirement, i.e., GluRS is a ribonucleoprotein and GluQRS is not. Furthermore, GluQRS transfers the glutamyl moiety not onto the tRNA 3’ end, but onto the modified nucleotide queuosine in the wobble position of the tRNAAsp anticodon. Thus, despite its independence from tRNA for aminoacylation, and its lack of an anticodon-binding domain, GluQRS must bind the tRNAAsp anticodon stem-loop to transfer the activated Glu-AMP to the everted queuosine residue. No other amino acid is adenylated by GluQRS in presence or absence of tRNAGlu or tRNAAsp, notably not aspartic acid (Asp) despite its cognate tRNA being the natural substrate. GluQRS is reported to bind weakly to Glu and to ATP, and more strongly to glutamol-AMP (Glo-AMP; IUPAC: (4*S*)-4-amino-5-[[(2*R*,3*S*,4*R*,5*R*)-5-(6-aminopurin-9-yl)-3,4-dihydroxyoxolan-2-yl]methoxy-hydroxy-phosphoryl]oxypentanoic acid (structure: Supplementary Figure S1), in which the alpha-carboxylate group of Glu is substituted by a hydroxymethyl group that is esterified to the AMP phosphate [15]. Glo-AMP is a more stable analog of the native phosphoanhydride reaction product Glu-AMP. The affinity (K_d_) of GluQRS for Glu is reported to be ~2 mM in absence of ATP, and ~0.05 mM in its presence [14]. Such relatively weak binding might be related to the very high intracellular concentration of L-Glu in *E. coli* (~10 mM; [16]).

**Figure 1:**
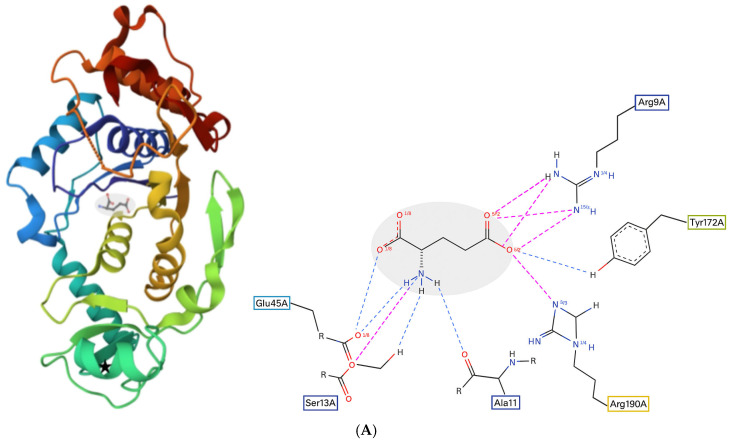

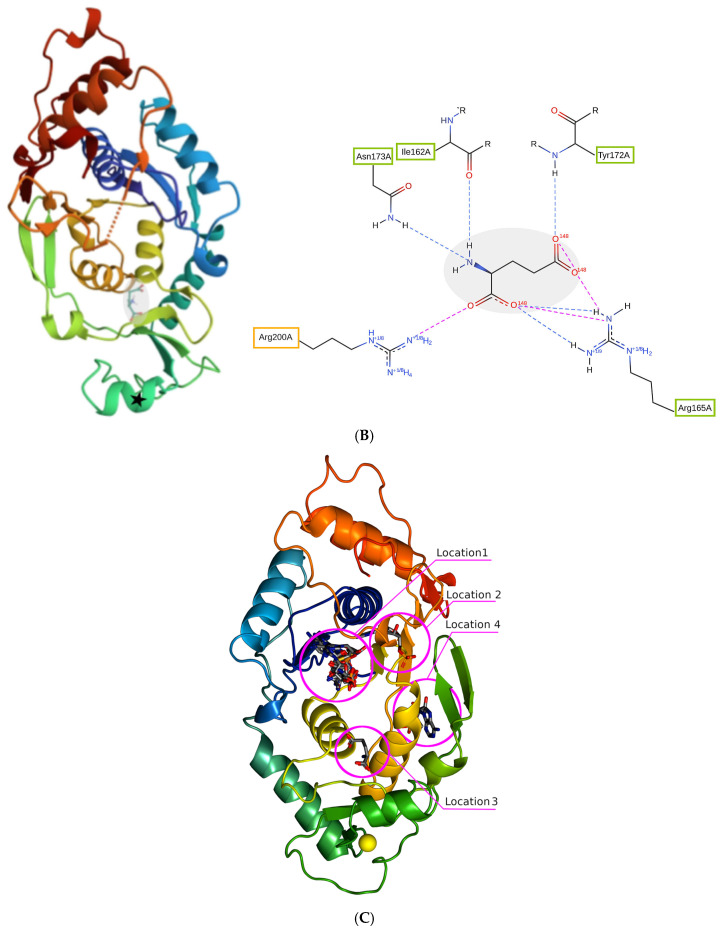
GluQRS interactions with native ligand Glu. **Left**, secondary structure cartoon of GluQRS in rainbow colors from N-terminus (blue) to C-terminus (red). Missing residues Asn233 and His234 are indicated by the dashed orange line. The location of the zinc ion that was removed from the PDB file is marked by a black star. **Right**, PoseEdit view of residues surrounding the ligand. Functional group contacts are indicated by residue name and number in boxes color-coded to their locations on each cartoon (A, unit cell chain A). Dashed lines indicate predicted hydrogen bonds (blue) and charged-group proximities (pink). (**A**). Ligand Glu bound in the GluQRS crystal (PDB:4A91). The Glu ligand is shown in a grey oval shadow as skeletal model with atomic colors and black carbons. (**B**). As in panel (**A**) but with Glu docked at novel Location 3 identified in panel (**C**). The protein is rotated from panel (**A**) to best show the location of Glu. (**C**). Ligand locations examined in this work and tabulated in Table 1. The protein orientation is that of panel (**A**). The zinc ion (yellow sphere) is shown in the site it occupies in the GluQRS crystal structure. The ligands are represented simultaneously but the figure is not meant to imply simultaneous occupancy of different locations. Location 1 is the native site for Glu as observed in the GluQRS crystal structure. Some of the ligand orientations observed for Glu when docked and simulated at this location are shown. Location 2 marks the approximate site modeled for AMP by Blaise et al. [14]. Location 3 illustrates Glu in the novel site identified in this work. Location 4 illustrates AMP in the novel site identified in this work.

Figure 1A left panel show cartoon depictions of the crystal structure of GluQRS from PDB file 4A91; the bound amino acid ligand Glu is shown, and the position of the zinc ion present in the crystal structure is indicated, although it was removed in the majority of the simulations reported in this work. Figure 1C identifies four distinct amino acid ligand locations examined in this work, and for reference it includes the zinc ion in its exact location in PDB:4A91. Simulation results show that the protein remains fully folded in absence of zinc (Supplementary Figure S10), and that simulations of GluQRS and GluQRS/Glu with and without bound zinc resemble each other closely. The dynamics in the zinc-binding region are similar to those observed in other mobile loop regions of GluQRS and do not propagate beyond the empty zinc site. Although crystals of zinc-free GluQRS/Glu have not been reported to date, zinc binding in GluQRS was eliminated by mutating its coordinating residues [17], resulting in slow aggregation that could be reversed by binding of Glu or ATP, indicating that each of these ligands can bind in absence of zinc. The available Zn concentration in *E. coli* is in the fM range [18], whereas the concentration of Glu is ~10 mM, suggesting that the protein exists at times with Glu, but not Zn, bound.

This work does not aim to examine functional GluQRS; rather, it aims to examine the nature of ligand binding during simulation to shed light on what binding may look like. The guiding premise is that the results for GluQRS/Glu without zinc report usefully on what binding of Glu looks like in simulations with zinc-free GluQRS. The results and conclusions for GluQRS/Glu in presence of zinc may well be different; that outcome would be independent of the picture developed here of what binding looks like in its absence. As documented in this work, at some times during the simulations in absence of zinc the results do resemble quite well the position and interactions of Glu with GluQRS that are observed in the crystal with bound zinc, indicating that zinc is not a requirement to capture the crystal behavior for Glu. This finding means that the behaviors reported here cannot be ascribed simply to the absence of zinc, and it reinforces the fact that the crystal structure is just one snapshot from the protein’s range of dynamic behaviors.

The PoseEdit view of Figure 1A right shows that in GluQRS/Glu crystals the Glu ligand is surrounded by six residues that form a network of bonding interactions including thirteen inferred hydrogen bonds (blue), four of which involve at least one charged group (pink). The protein functional groups forming the bonding interactions include the Arg9 guanidino, Ala11 carbonyl, Ser13 hydroxyl, Glu45 carboxylate, Tyr172 hydroxyl, and Arg190 guanidino. These groups originate in diverse elements of the protein’s tertiary structure, as indicated by the rectangles enclosing numbered residues that are color-coded to their secondary structure locations. (Note that PoseEdit representations maximize the visibility of bonding interactions in 2D but do not necessarily display the 3D disposition of residues or ligands accurately.) The GluQRS binding site for Glu is thus an entirely typical ligand-binding site sharing the features of many *bona fide* binding sites in proteins seen in the PDB. These features include combinations of different types of interactions originating from multiple residues in distant parts of the protein, reflecting engagement of its tertiary structure, and triangulation with multiple atoms from distinct functional groups of the ligand.

MD simulations with GluQRS. PDB file 4A91 containing GluQRS with Glu and a zinc ion bound [14] was selected as the starting structure for simulations in this work. The zinc ion was removed prior to preparing the file for most simulations reported in this work, as indicated. In the various simulations reported here the Glu ligand was either left in, removed, or replaced by docking with Glu or another amino acid, as indicated. The PDB structure file contains a two-residue gap where Asn233 and His234 in the middle of a twenty-residue irregularly structured segment are missing from the model. These were repaired for simulation (but not for docking) in one of two ways as indicated in each simulation: in some cases, GROMACS (version 2021.4) replaced the gap with an artificial peptide bond between residues 232 and 235; in other cases, the gap was replaced by the two native residues that were modeled as an energy-minimized loop in YASARA (version 18.42.4S).

**Figure 2:**
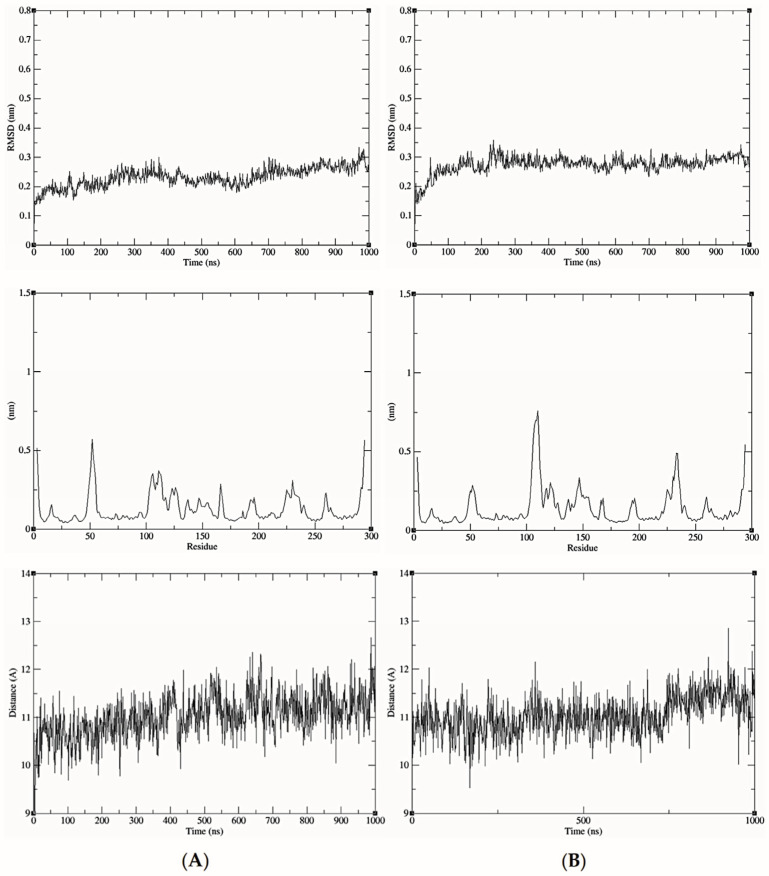
Results of simulations with GluQRS crystal structure without Glu. The crystal zinc ion and Glu ligand were both removed. Top, RMSD; middle, RMSF calculated for alpha carbons during the entire one-microsecond time course; bottom, distance between alpha carbons of Val176 and Ile188. (**A**). The two-residue gap at missing residues Asn233-His234 was repaired by GROMACS (version 2021.4) with the introduction of an artificial peptide bond between residues 232 and 235. (**B**). The gap was replaced by a modeled, energy-minimized loop consisting of residues Asn233 and His234.

MD simulations of GluQRS without Glu. Independent simulations were initiated from the prepared crystal structure file with the Glu ligand removed to evaluate the protein’s dynamics when both the Glu and zinc ligands are absent. Figure 2 shows results from one-microsecond simulations of GluQRS without bound Glu with an artificial peptide bond replacing the gap at residues 233–234 (Figure 2A), and with a modeled, energy-minimized Asn233–His234 loop replacing the gap (Figure 2B). The RMSD plot of Figure 2A reaches a stable plateau by ~700 nsec at ~0.25 nm with deviations of +/− ~0.1 nm. Inspection of the structures during the trajectory indicates that the protein fold is well maintained throughout, and that two local regions persist in making excursions of up to ~1 nm, as shown by the RMSF plot. The RMSD plot for simulation of the protein lacking Glu and zinc ligands with the gap replaced by a modeled Asn-His loop (Figure 2B) shows that by ~200 nsec the structure reaches a stable RMSD plateau at ~0.3 +/− 0.05 nm. The corresponding RMSF plot shows that the regions of major chain excursions are the same in both cases, though damped in Figure 2B to maxima of ~0.5 nm in RMSF. One of the two regions of high mobility in both simulations, residues ~223 to 242, overlaps with the long irregularly structured segment containing the two missing residues. An earlier crystal structure of the unliganded protein before its functional ligands were identified (PDB:1NZJ; [13]) lacked the entire segment from 224 to 236, suggesting high mobility. Although the modeled PDB:4A91 structure used here does contain most of these irregular residues, the model shows that they project away from the main body of the protein, with few tertiary interactions. The mobility of such a segment is not unexpected [19].

The second segment with high mobility in the RMSF plots, residues ~101 to 129, encompasses the zinc-binding site, including residues Cys101, 103, and 119 and His118 responsible for coordinating the missing zinc ion. In the crystal structure where zinc is present residues 104 to 111 inclusive form a short helix that is quite solvent-exposed on the protein surface, and thus may be less stable in the absence of the constraints imposed by bonding of zinc with residues in the segments flanking the helix. In addition, three of the residues of this short helix have beta-branched sidechains (Thr104, Ile108, and Ile111). Beta-branched residues are disfavored statistically in helices [20], perhaps because when the backbone is in helical conformation these sidechains have access to only one rotamer due to steric hindrance [21]. All these factors local to the ~101 to 129 and ~223 to 242 regions, including the absence of other secondary structure elements, conspire to yield the relatively unstable RMSDs of the protein in absence of both Glu and Zn. The results of these simulations thus indicate that the mobility of the protein does not depend on how the two-residue gap is repaired. In all simulations in this work the mobility of the protein was assessed in RMSF plots and in VMD by using heat mapping to indicate regions of higher and lower mobility (Supplementary Figure S2). The results support the conclusion that the large values of RMSD reflect excursions of the mobile-loop regions of the chain.

The maintenance of the protein fold during these simulations was further evaluated by distance measurements reflecting the tertiary structure. Residues Val176 and Ile188 face the Glu-binding site from opposite sides in the crystal structure but do not interact with the ligand. The distance between their respective alpha carbons was chosen as an indicator of motion in the central part of the fold away from the mobile loops. The bottom panels of Figure 2 show that this distance, ~11 Å, varies by only ~1 Å during the one-microsecond time course of the simulations, and with little correlation to the RMSD plot. This result strengthens the suggestion that the relatively large RMSD values reflect the local motions of the two long loop regions, and do not indicate instability of the protein fold; inspection of all reported structures in VMD invariably confirmed stability of the fold. Thus, relatively large RMSD values can be expected for this protein.

**Figure 3:**
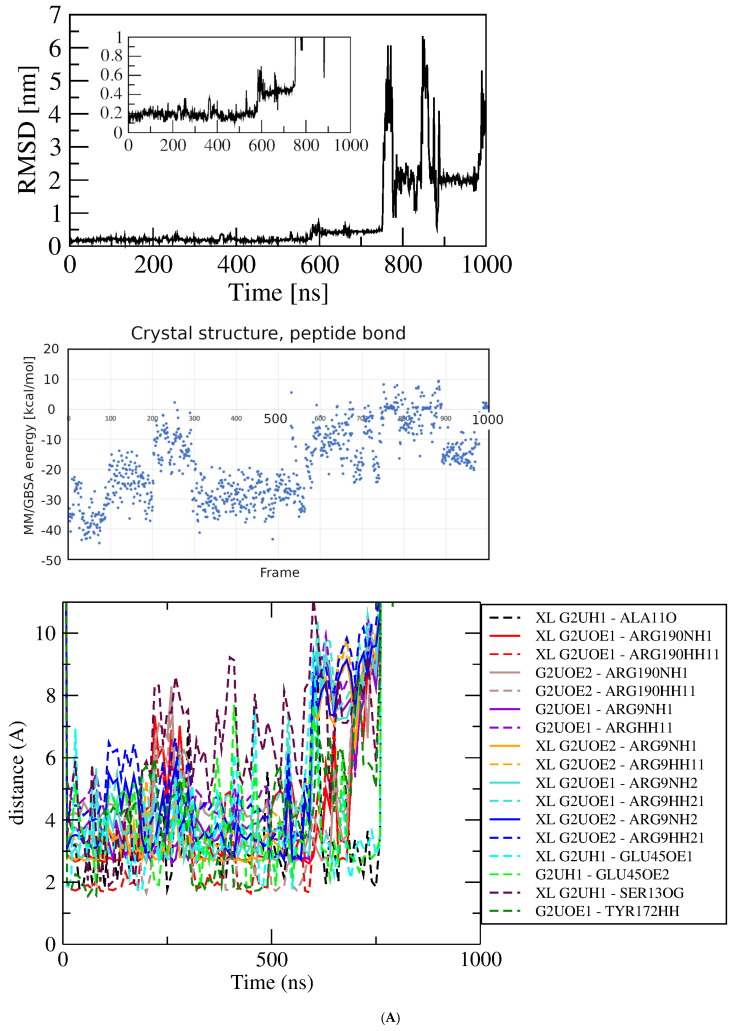

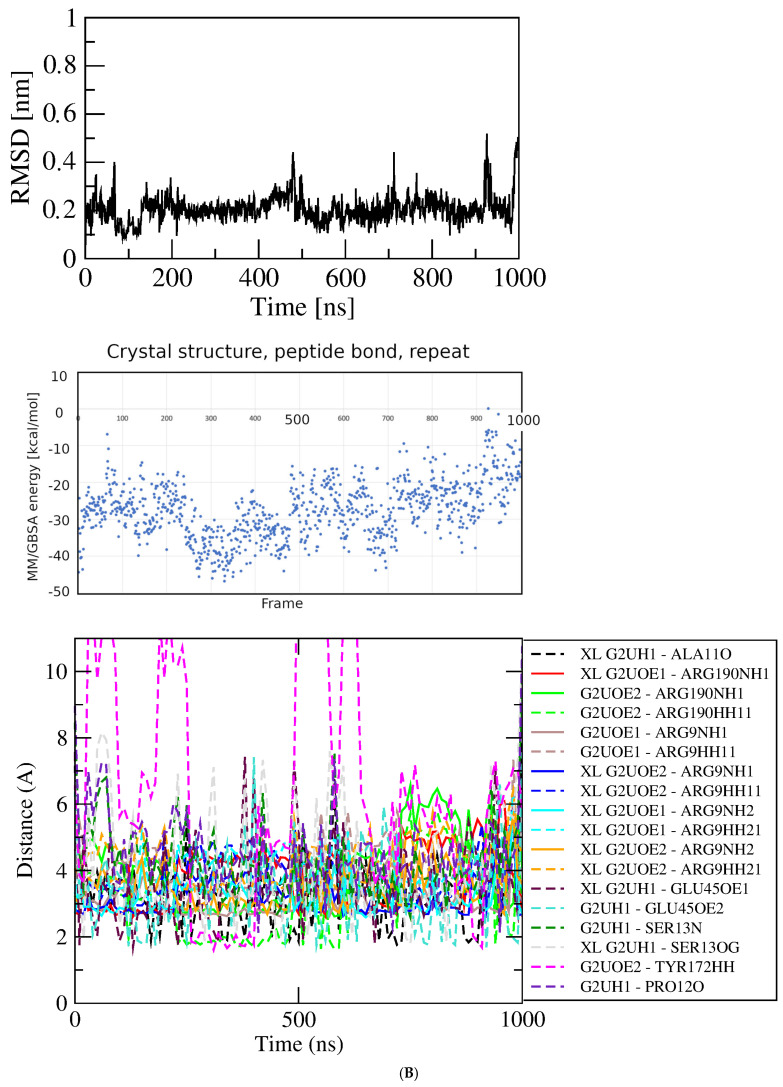

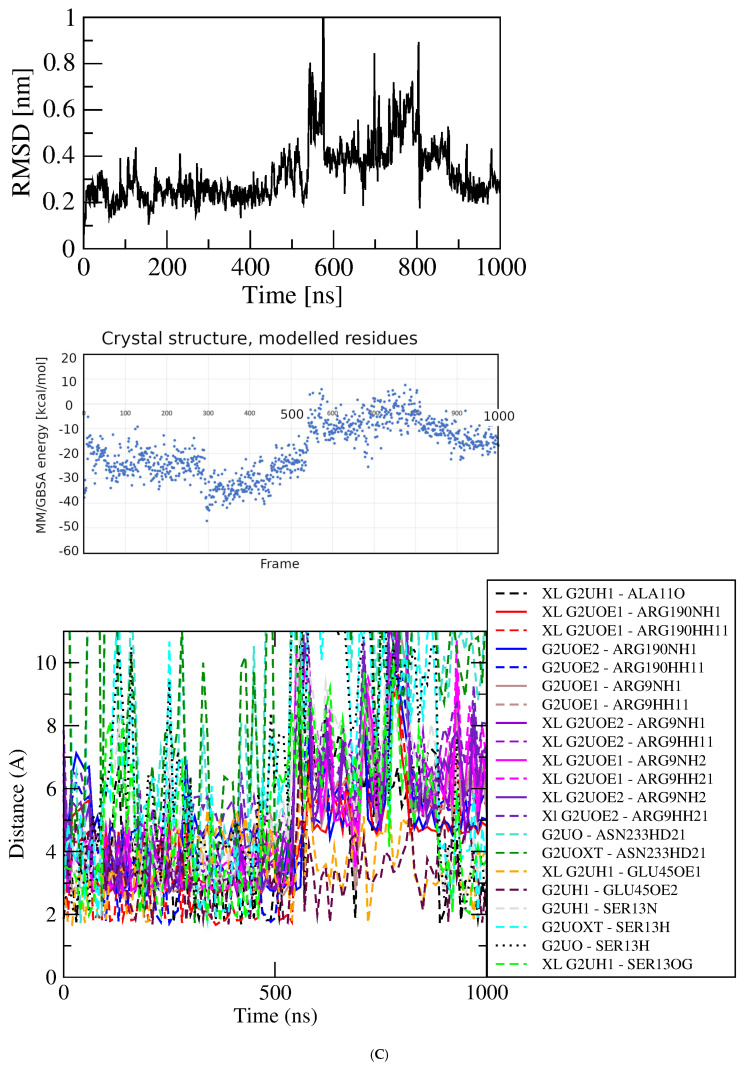

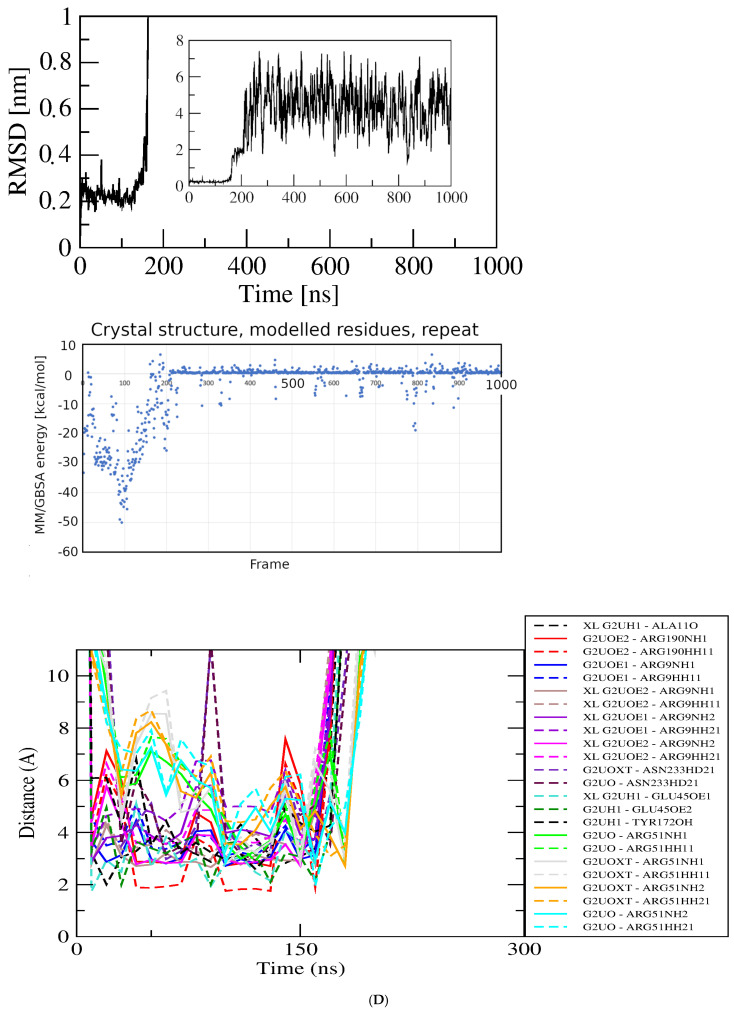
Results of simulations with GluQRS/Glu crystal structure. The crystal zinc ion was removed, and the two-residue gap repaired as indicated. Panels are aligned in the x-axis dimension to show correspondence of time and frames except in (**D**). **Upper**: RMSD at full y-axis scale and expanded y-axis scale (where inset is added, its location and axis scale differ to optimize space). **Center**: interaction energy calculated by MM/GBSA. **Lower**: atomic distances during the MD trajectory. The key (inset) identifies atom pairs used in distance measurements made in VMD as described in the text: dashed lines, H bonds; solid lines, interactions involving any charged group; XL, atom-pair interactions noted in the crystal structure. (**A**). The two-residue gap at residues Asn233-His234 repaired by introduction of an artificial peptide bond between residues 232 and 235. (**B**). Independent replicate of the simulation in 3A. (**C**). The two-residue gap repaired by introduction of a modeled loop consisting of residues Asn233 and His234. (**D**). Independent replicate of the simulation in 3C. Note that distances in (**D**) are plotted for 0 to ~160 nsec only, and with ~five-fold x-axis scale expansion, so the time scales do not align.

MD simulations of GluQRS/Glu. In describing the results of simulations with protein/ligand complexes the ligand is referred to as being in an “associated” state where it “interacts” with protein functional groups, without implying that the observed features signify “binding.” Four independent simulations were initiated from the prepared crystal structure file that has Glu bound as displayed on Figure 1 upper (location 1 in Figure 1C); two simulations had the 233–234 gap replaced by a peptide bond, and two had the gap replaced by a modeled loop. Figure 3A shows RMSD, MM/GBSA system energies, and atomic-distance results from a one-microsecond simulation of the GluQRS/Glu crystal complex with a peptide bond replacing residues 233–234. The RMSD plot (top panel) shows that the structure reaches an apparently equilibrated state within the first few nsec after steepest-descent minimization and NPT-NVT equilibration. A stable RMSD value of ~0.25 nm with deviations of less than ~0.1 nm persists for over 550 nsec, indicating an equilibrated state. Some brief excursions to ~0.35 nm occur occasionally in which the ligand loses its close distances to Glu45 and Tyr172 due to a change in Chi1 to ~+60 degrees from its usual values that alternate between ~−170 and ~−60. During these excursions the ligand maintains close distances with residues Arg9, Ser13, and Arg190. Thereafter until ~750 nsec the RMSD value increases to a new plateau at ~0.5 nm and the ligand remains at the crystal site with high conformational mobility. During the next ~150 nsec the ligand twice leaves the crystal binding site before returning to location 3 near the zinc-binding site for the final 100 nsec.

The energies of interaction calculated over the time course using MM/GBSA are shown in the middle panel. The GBSA system energy varies widely during the first ~300 nsec, from less than ~−40 kcal/mol to ~zero without any obvious correlation to this stable region of the RMSD plot. The most persistent stable energies occur from ~350 to ~500 nsec, with an average value of −29.00 +/− 4.49 kcal/mol, despite some significant RMSD excursions during ~350 to ~400 nsec. Interestingly, the time period with lowest deviation in energies (but not the lowest energy, only −11.47 +/− 6.40), occurs in the final ~100 nsec, even though during that period the RMSD deviations are consistently as large as the largest deviations in the first ~500 nsec, and the ligand has shifted from the crystal Glu binding site to location 3 near but not in the crystal zinc binding site.

The bottom panel shows distances between pairs of functional group atoms for the first ~750 nsec, i.e., during the time the ligand is located at the crystal site. All distances consistent with hydrogen (H) bonds or ionic interactions are plotted. In order to distinguish H-bond distances from ionic interactions between the same atom pairs, H-bond distances were measured from heavy atom to proton, not heavy atom to heavy atom as is the convention. Thus, the lowest populated bond distances are ~1.9 Å corresponding to presumptive H bonds, and distances around 2.9 Å indicate ionic interactions that are also presumably hydrogen-bonded. When these distances are frequently populated during the simulation their values become visible as distinct horizontal lines of color. Longer distances reflect charged-group interactions too distant for H bonds. The pattern of distances over time indicates that many atomic distances in the crystal structure are observed only rarely and transiently in the simulation, and several close distances occur in the simulation that are not observed in the crystal structure. Several of the H-bond distances in the crystal structure, including GluNH with SerOH, GluOE1 and GluOE2 with Arg9 guanidino, and GluNH with AlaC=O, are often absent, notably during the RMSD plateau from ~350 to ~500 nsec that is apparently stable as judged also by GBSA energy of −29.00 +/− 4.49 kcal/mol. All the characteristic distances expected for a complex with the numerous close atomic distances seen in the crystal are populated only sparsely in the simulation. The overall frequencies of individual atomic distances that are populated at least 10% of the time during the simulation are summarized in a histogram (Supplementary Figure S3).

The seemingly inconsistent results among the RMSD, GBSA, and bond-distance analyses suggest that the view in the crystal structure can differ substantially from the view observed in the simulation. Similarly as for the protein without ligands, a direct repeat of this simulation with independent initial velocities yields somewhat different details but an overall similar picture (Figure 3B). The RMSD stabilizes within a few nsec at ~0.2 nm but with slightly larger and very brief deviations of ~0.2 nm that do not indicate excursions of the ligand from the crystal Glu binding site. The overall GBSA energy is −27.64 +/− 7.79 kcal/mol. As before, the correspondence between the RMSD and GBSA plots is weak, with only a short period of concordance between maximum GBSA stability and minimum RMSD deviation at ~250 to ~350 nsec. Only during that limited period are the majority of bond distances in the crystal structure observed in the simulation. These results indicate that repeated simulations of the same system, though differing in details, yield a consistent overall picture that reinforces the suggestion that the crystal and MD views of binding differ significantly in bonding, both in the identities and distances of the atomic partners and the duration of bond occupancy.

Two independent simulations were completed starting from the crystal structure with the two-residue gap replaced by a modeled, energy-minimized Asn233-His234 loop. In one repeat the RMSD quickly reaches a plateau at ~0.25 nm with deviations of ~0.1 nm and one short excursion to ~0.4 nm, after which the ligand dissociates and does not return. During the initial ~150 nsec plateau the ligand remains associated with the protein, and the energy calculated for the system reaches a low extreme briefly at ~−50 kcal/mol, but does not in general mirror the stability of the RMSD plot. While associated with the protein the ligand makes only few and brief close approaches to functional groups of the crystal Glu binding site, and repeatedly approaches residues Arg51 and Asn233 that are not contacts made in the crystal. These residues lie in loops directly above the crystal binding site close the protein surface. Throughout this initial RMSD plateau, close approaches of the ligand to residues at the crystal binding site alternate with those near the protein surface, indicating that the ligand repeatedly samples a partly dissociated state. The only exception, a H-bond distance from one ligand sidechain carboxylate oxygen to the Arg190 guanidino group, can be maintained even as the ligand migrates toward the surface, due to the long Arg sidechain and the linear ligand conformation. These results indicate that although the ligand starts out in the crystal binding site it begins immediately to sample partly dissociated states, then soon leaves the protein entirely and does not return in the following ~800 nsec.

**Figure 4:**
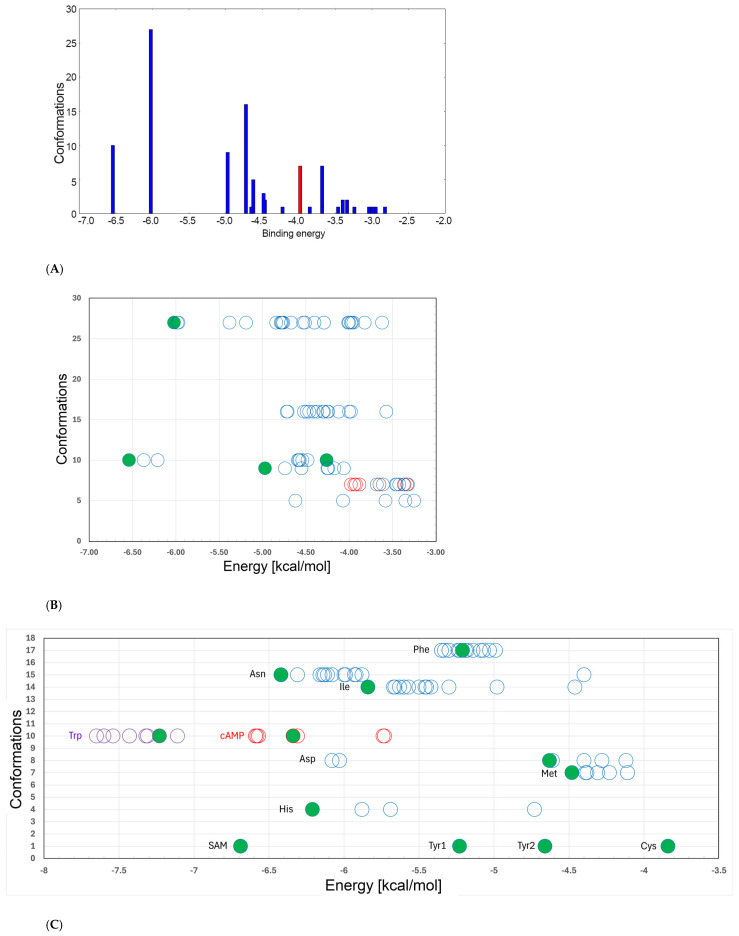
Distribution of energies for GluQRS docked ligands. (**A**). Bin energies for docked native ligand Glu. The x axis “binding energy” is calculated in vacuum by AutoDock4 in units of kcal/mol; the y axis is number of conformations (see text). Two bins have seven conformations; one of them is in red to distinguish it in panel (**B**). (**B**). Relationship of bin energies to pose scores. Axes as in panel (**A**). Seven of the AutoDock bins in panel (**A**) are plotted at the y-axis value representing the number of conformations in each bin. Circles indicate the energies calculated by AutoDock4 for each individual pose within its bin, with the most negative energy being the bin energy of panel (**A**). Green circles mark the poses chosen for the four simulations with docked Glu. (**C**). Pose scores for non-native docked ligands. Energies are calculated by AutoDock4 for all poses (circles) in the AutoDock bin from which each non-native ligand pose was chosen for simulation (green circles); y-axis values indicate the number of conformations in that bin. Ligands are labeled to the left of the lowest energy of the bin; Trp and cAMP are purple and red, respectively, to distinguish them.

Docking. All remaining simulations reported here began from structures obtained through docking of the protein with ligands using global docking in AutoDock4. The protein and ligand are placed separately within the simulation box, i.e., the ligand is not placed deliberately on the protein. The protein is treated as static, the ligand as unconstrained, and the ligand is allowed to explore the simulation box freely. From up to 2.5 million iterations one hundred poses are returned for each protein–ligand pair, grouped by the program into bins, and presented in the form of a histogram (Figure 4A). However, the bins do not represent common energy groups as the AutoDock histogram implies. Rather, each bin represents a group of ligand poses at a location on the protein that is defined by the identities of the surrounding residues, but with diverse configurations including variation in bond numbers, participating functional groups, ligand orientation and conformation, and, importantly, varying docking scores calculated in vacuum. As a result, the constant widths of the bins do not correspond to constant score ranges. Instead, each bin is plotted in the AutoDock histogram at the lowest calculated score (strongest predicted “binding”) of any pose in that bin, but only the lowest-energy pose(s) in the bin will have this score. Therefore, the score plotted for each bin in the AutoDock histogram will be referred to here as the bin energy. The 100 docked poses of the native ligand Glu with GluQRS fall into twenty-one such bins with calculated bin energies ranging from ~−6.5 to ~−2.8 kcal/mol, as shown in Figure 4A.

Given this non-standard meaning of the histograms produced by AutoDock it proved informative to create a plot in which the AutoDock scores for each individual pose are displayed for all poses within each bin. Figure 4B is such a plot for the seven most populated and lowest-scoring (strongest predicted binding) bins of panel A for Glu binding to GluQRS; bins with fewer than five poses, or calculated scores weaker than −4.5 kcal/mol, are not considered further. The large range of scores within each bin is clearly evident. For example, the ten poses at bin score ~−6.5 kcal/mol in panel A have a bimodal distribution of scores in panel B with different population sizes: three poses around −6.5 kcal/mol and seven around −4.5 kcal/mol. Similarly large ranges are observed for all seven bins with bin scores ranging from ~−6.5 to ~−3.2 kcal/mol and containing in total 81 of the 100 AutoDock poses. The 27 poses at the next-lowest bin score (~−6.0 kcal/mol) display a more continuous population distribution than observed for the ten poses of the first bin. Note that the second bin contains eleven poses with scores lower (stronger) than for the majority of poses in the first bin. In total sixteen poses in four bins on Figure 4B have scores < ~−4.6 kcal/mol, i.e., stronger than the group of weaker-scoring poses in the bin at ~−6.5 kcal/mol. This observation suggests that similar scores for a ligand can be found for presumably distinct locations on the protein with different bonding interactions.

This suggestion was tested by inspection of structures in VMD to determine the structural locations of ligands on the protein for all 21 bins in Figure 2A. Thirteen poses, in three bins, are all located at the crystal binding site for Glu, with calculated pose scores ranging from ~−6.5 to ~−4.5 kcal/mol. This range of pose scores is approximately correlated with the numbers of contact residues and interactions with Glu, with the strongest predicted poses displaying all the interactions observed in the crystal structure, and the weaker poses displaying different subsets. The remaining eighteen bins contain poses at locations other than the crystal binding site for Glu. These bins represent eight novel protein locations when considered according to the principles used here rather than by AutoDock grouping. Nearly half the total, comprising 39 of the 100 poses in four bins, are in location 3 in the vicinity of the zinc-binding site distant from the Glu crystal site (Figure 1C). This group is defined by poses with slightly different surrounding residues at bin scores ~−6.2 kcal/mol with 27 members ranging to ~−3.6 kcal/mol; ~−5.0 kcal/mol with nine members ranging to ~−4.0 kcal/mol; ~−3.8 kcal/mol with one member; and ~−3.4 kcal/mol with two members ranging to ~−3.0 kcal/mol. These poses found independently in multiple runs suggest a relatively robust local energy minimum that might indicate potential secondary interaction at location 3 for Glu. Half the remaining novel locations are represented by more than one bin, but no other location is dominant.

Pose selection. Given the above results indicating limited confidence in the calculated AutoDock pose scores, the scores were not used to prioritize docked poses for MD analysis of ligands. Rather, an effort was made to identify among the docked poses sites with the potential for binding based on known protein–ligand binding sites by manual inspection in VMD and with PoseEdit views of intermolecular contacts. Note that the two-dimensional PoseEdit views do not reflect the relative positions of interacting residues surrounding the ligand; thus, positional changes in the PoseEdit views do not necessarily imply changes in relative orientation, which were always confirmed in VMD. Poses prioritized for MD were those displaying some or all of the following characteristics that are commonly found for known protein–ligand interactions: diversity of number, type, and location of bonds between partners, i.e., combinations of hydrogen bonds, ionic interactions, and hydrophobic interactions with distinct functional groups of the ligand, preferably from multiple residues that triangulate with multiple atoms of the ligand; bonding with distant parts of the protein as judged by the sequence numbers of interacting residues, reflecting engagement of the protein’s tertiary structure; binding locations judged subjectively to be recessed from the protein surface, without using any specific distance or solvent-accessibility metric. Poses sharing these features were not always those with the lowest scores calculated by AutoDock4. The pose chosen for simulation from a given bin was the one judged to have the best match to the above criteria. Table 1 lists the AutoDock4 scores and locations for each ligand examined in this work.

**Table 1:** Summary of all simulations. Column one, ligand identity, manner of repairing the two-residue gap, and initial location of the ligand in each simulation; column two, time periods of simulations used for calculating GBSA energies; column three, energy of docked structures calculated by AutoDock4; columns four and five, GBSA energies (mean, m and standard deviation, s); column six, intrinsic specificity ratio calculated from GBSA energies as described in the text.

Ligand/Gap/Location	Time (ns)	AutoDock (kcal/mol)	GBSA μ (kcal/mol)	GBSA σ (kcal/mol)	ISR
Location 1					
Glu/peptide bond/crystal site	0–1000		−18.25	12.26	2.10
	50–550		−25.09	8.82	
	350–500		−29.00	4.49	
replicate	0–1000		−27.64	7.79	2.36
replicate	200–450		−33.62	6.29	
Glu/modeled loop/crystal site	0–1000		−18.60	10.72	2.65
	200–400		−25.42	1.74	
replicate	0–1000		−4.43	10.49	4.35
replicate	1–150		−26.12	9.76	
Glu/peptide bond/dock bin ~−6.5 kcal/mol	–	−6.54			
	0–1000		−29.31	5.69	2.06
	140–350		−31.52	3.96	
replicate	–	−6.54			
replicate	0–1000		−31.13	5.58	3.20
replicate	250–400		−34.38	2.84	
Glu/peptide bond/dock bin ~−6.5 kcal/mol	–	−4.26			
	0–1000		−21.50	7.03	2.35
	250–400		−21.00	6.14	
Asp/peptide bond/dock bin ~−6.0 kcal/mol	–	−4.63			
Asp	0–1000		−29.60	4.42	2.80
Asp	110–890		−30.52	3.97	
Asn/peptide bond/dock bin ~−6.5 kcal/mol	–	−6.42			
Asn	0–1000		−15.92	3.40	2.97
Asn	1–800		−15.80	3.12	
His/peptide bond/dock bin ~−6.2 kcal/mol	–	−6.21			
His	0–1000		−19.27	6.72	2.19
His	400–600		−19.97	3.21	
Ile/peptide bond/dock bin ~−6 kcal/mol	–	−5.84			
Ile	0–1000		−22.52	2.66	2.06
Ile	440–630		−22.36	2.66	
Met/peptide bond/dock bin ~−4.5 kcal/mol	–	−4.48			
Met	0–1000		−32.15	3.80	2.85
Met	120–740		−32.27	3.60	
SAM/peptide bond/dock bin ~−6.5 kcal/mol	–	−6.69			
	0–1000		−33.46	7.25	2.83
	450–750		−38.05	7.63	
cAMP/peptide bond/dock bin ~−6.5 kcal/mol	–	−6.34			
	0–1000		−56.19	7.38	1.87
	500–750		−57.46	5.09	
AMP/peptide bond/dock Glu crystal site	–	−4.94			
	0–1000		−14.25	10.55	3.39
	550–650		−23.33	6.48	
AMP/peptide bond/dock novel site	–	−6.24			
	0–1000		−14.54	9.12	2.57
	660–750		−23.38	6.37	
**Location 2**					
AMP/peptide bond/dock AMP modeled site	–	−4.96			
	0–1000		−23.15	5.12	3.88
	500–700		−22.69	4.65	
**Location 3**					
Glu/peptide bond/dock bin ~−5.0 kcal/mol	–	−4.97			
	0–1000		−5.50	7.91	2.72
	460–700		−13.67	5.68	
**Location 4**					
AMP/peptide bond/dock novel site	–	−6.24			
	0–1000		−14.54	9.12	2.57
	660–750		−23.38	6.37	

All ten poses of Glu in the bin with strongest (lowest) bin score resemble the crystal structure very closely as shown in the PoseEdit views of Supplementary Figure S4, with the pose at ~−6.5 kcal/mol being essentially identical as judged in VMD. The principal structural difference between the two groups of poses in this bin (at ~−6.5 kcal/mol and ~−4.5 kcal/mol), as confirmed in VMD, is the formation of contacts with the ligand by six vs. five residues, respectively, and in the orientation of the ligand relative to the protein (recalling that in docking the protein is fixed). In the group at −6.5 kcal/mol, the ligand sidechain carboxylate engages the two Arg residues and Tyr172 as in the crystal, whereas in the group at −4.5 kcal/mol these residues engage the ligand’s alpha substituents. Within each group some bonding details and/or identities of the interacting residues also differ. Despite these differences, all ten docked poses are similar to each other, with a common set of five or six surrounding residues making a total number of bonding interactions ranging from eleven to fourteen. Some identical poses are represented more than once within each group, suggesting the potential relevance of those poses.

Some of the ligands shown on Figure 4C were investigated only partially or not at all. Tryptophan (Trp), phenylalanine (Phe), and cysteine (Cys) were simulated from docked poses at the crystal site for binding of Glu. In simulations of one microsecond both Trp and Phe soon move to novel locations. Despite displaying the strongest pose scores, even stronger than the substantially larger SAM and AMP ligands, Trp does not persist at any location and soon dissociates from the protein. Phe persists at its novel location with two periods of RMSD plateaus that were not investigated structurally, nor were Tyr1 and Tyr2, which were simulated from docked poses in novel locations. Cys has an early RMSD plateau with average GBSA energy −18.79 ± 3.86 kcal/mol (Table 1) that persists for ~200 nsec. Thereafter it is mostly dissociated from the protein until ~700 nsec, although it returns very briefly to a new location near the (empty) zinc-binding site, where the ligand recruits residues Tyr115 and Arg158. Despite apparently stable behavior in the final 300 nsec, analysis of the structures shows only one interaction, with the Gly191 amide, a new recruited contact residue in the immediate vicinity of the Glu crystal site. All other ligands are discussed in full detail below, and PoseEdit views of the selected docked pose for each of them are shown in Supplementary Figure S5.

**Figure 5:**
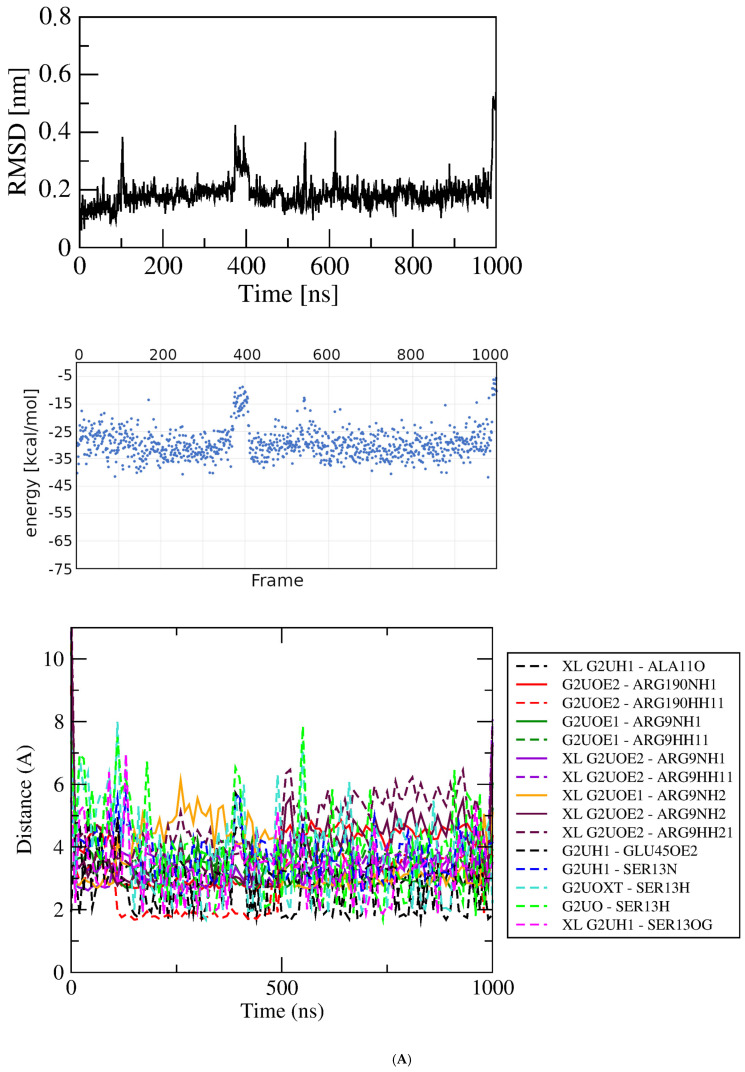

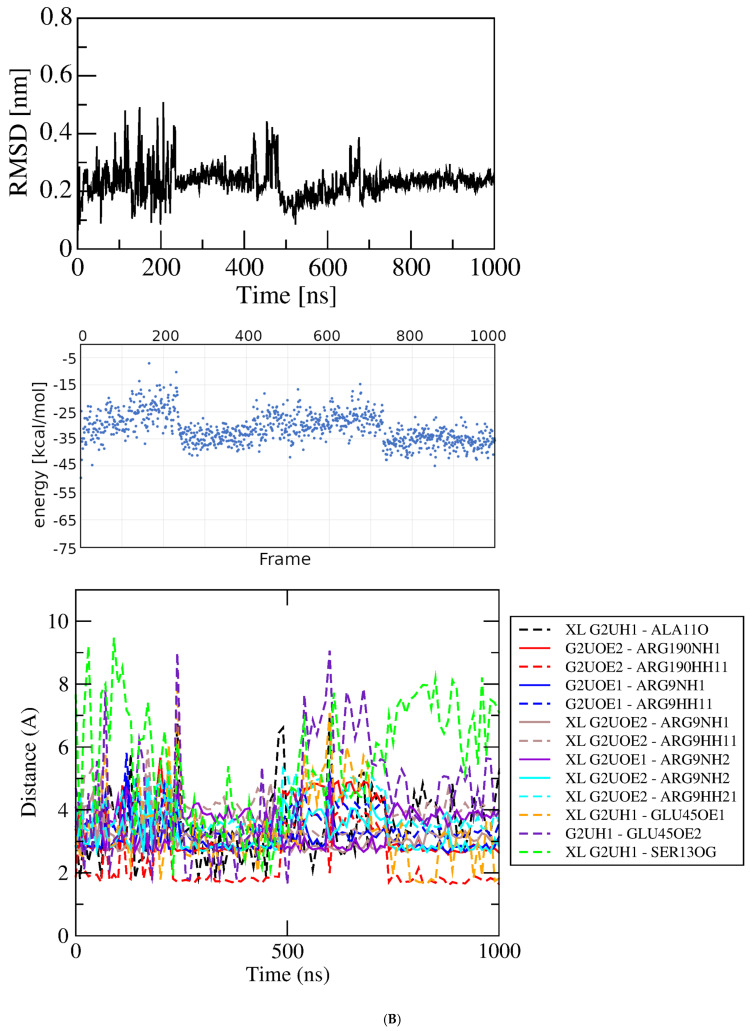

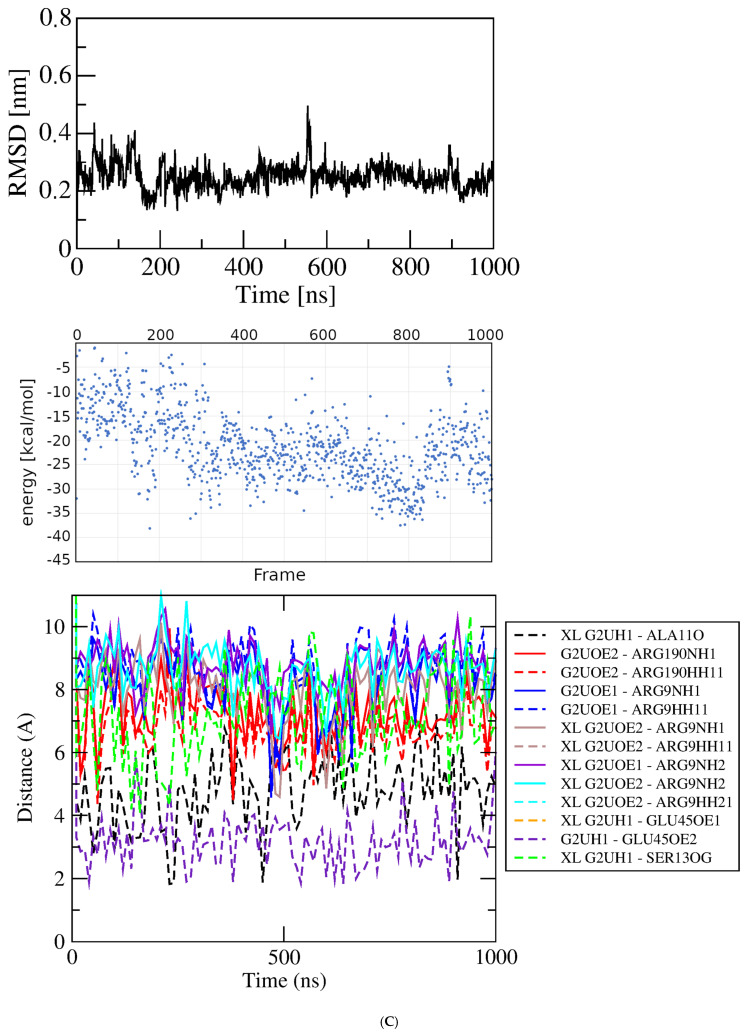

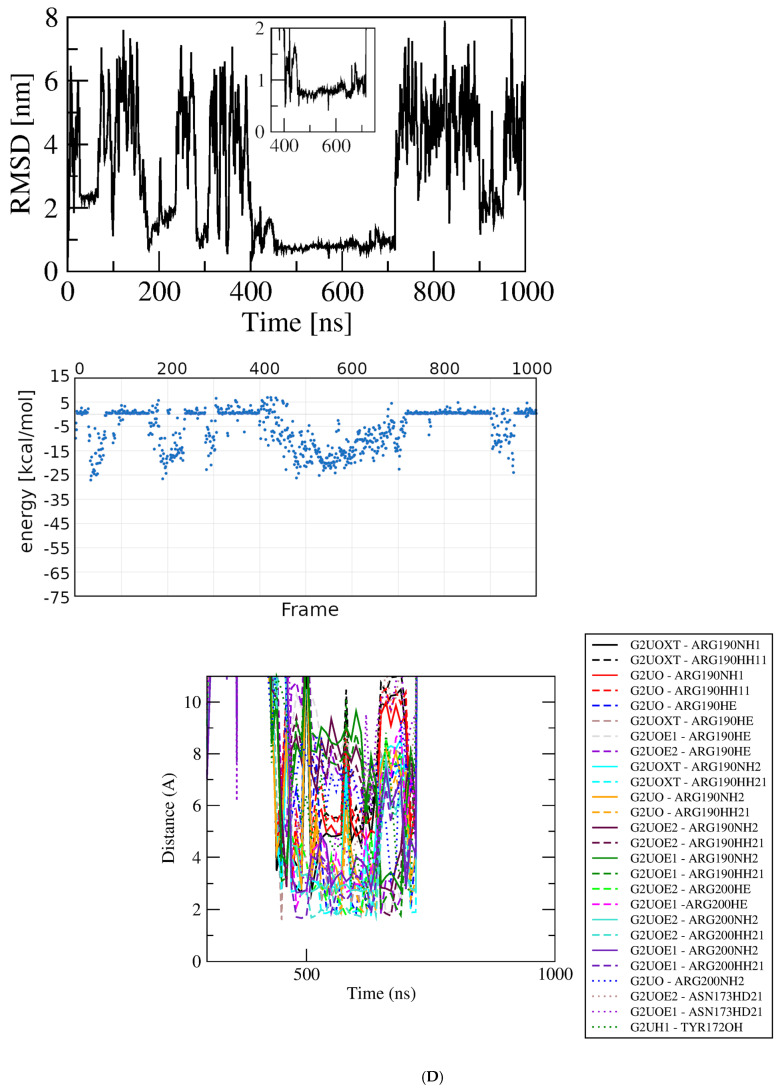
MD results for Glu docked in crystal site. Labeling as in Figure 3. (**A**) Glu crystal site −6.5 kcal/mol pose. (**B**) Glu crystal site −6.5 kcal/mol pose replicate. (**C**) Glu crystal site −4.5 kcal/mol pose. (**D**) Glu novel site (Location 3) −6.0 kcal/mol pose. Note time scale alignment in (**D**).

MD simulations of GluQRS with Glu docked at the crystal site. Three independent simulations were initiated from two poses of Glu docked at its crystal binding site for comparison with the crystal structure simulations: the poses with the highest and lowest calculated AutoDock4 scores from the bin at ~−6.5 kcal/mol of Figure 4A (~−6.5 and ~−4.5 kcal/mol in Figure 4B), with the ~−6.5 kcal/mol pose simulated twice (green in Figure 4B). This pose is essentially identical to the GluQRS/Glu crystal (Figure 1A) as judged in VMD and viewed in PoseEdit (Supplementary Figure S4). All three simulations had the two-residue gap replaced by GROMACS (version 2021.4) with a peptide bond between residues 232 and 235. Figure 5A displays the RMSD, GBSA, and atomic-distance plots for one MD simulation of GluQRS with its native ligand Glu initiated from the docked structure with AutoDock4 score ~−6.5 kcal/mol on Figure 4B. An early RMSD plateau at ~0.15 nm with deviation ~+/− 0.1 nm, and two later plateau regions, both at RMSD values ~0.2 nm +/− ~0.1 nm, define associated states at the crystal location as confirmed by VMD. The ligand remains at this location throughout the simulation except for very brief excursions to slightly distant locations marked by the larger deviations from the RMSD plateau values.

Although association is maintained in the plateau regions, examination of structures indicates that frequent changes occur in the number of contact residues and identities of some bonding groups, as shown in the distance plots. This result indicates that even though the docked structure replicates the crystal residue contacts and includes one additional hydrogen bond (Supplementary Figure S4), these interactions are not maintained. The system continues to evolve during the first ~600 nsec before reaching an apparently equilibrated state, although the RMSD deviations remain relatively large (+/− ~0.5 nm) even in the equilibrated phase, reflecting the mobile peripheral loops. During this phase the ligand repeatedly engages only five of the six residues contacted in the crystal and initial docked structures, consistently lacking contacts with Tyr172. The five residues do not maintain contact throughout, and, as seen in the simulations of the crystal structure itself, many atomic distances observed in the crystal structure are rarely, transiently, or never populated.

The results for the independent replicate of the simulation in Figure 5A are shown in Figure 5B. Here again, as seen in the simulations of the crystal structure, the details differ but the overall picture is entirely consistent: atomic distances that are fully populated in the crystal structure are rarely, transiently, or never populated. For both of these simulations the GBSA energy remains variable, 29.31 +/− 5.69 and 31.13 +/− 5.58 kcal/mol, and its variation is not correlated strongly with the RMSD. Figure 5C shows results for the third independent simulation of Glu docked at its crystal binding site. The docked structure selected for this simulation had the weakest score, ~−4.5 kcal/mol, of the ten poses at bin score ~−6.5 kcal/mol in Figure 4B. In this pose, the ligand orientation in the binding site is opposite that of the simulations of Figure 5A and B. Arg9, Tyr172, and Arg190 engage the Glu alpha-carboxylate group instead of its sidechain carboxylate, which interacts only with Ser13; Glu45 interacts with the ligand alpha-amino group, and Ala11 is not contacted in the docked structure. The ligand orientation does not change during the time course, and most interactions exchange frequently as seen in the distance plot.

Thus, the relative stability evidenced by the RMSD and GBSA values in the plateau regions of these three simulations belies frequent fluctuations in numbers of bonds and details of the bonding patterns among the interacting residues. Although the ligand remains at the crystal site throughout, the numbers and identities of contact residues and bonding interactions shift frequently over time, repeatedly decreasing and/or increasing. This dynamic picture of the associated state differs considerably from the crystal view.

MD simulation of GluQRS with Glu docked at a novel site. The 39 poses at bin scores of ~−6.0, ~−5.0, ~−3.8, and ~−3.4 kcal/mol were screened manually by VMD and PoseEdit to select an initial pose for MD. The pose with AutoDock4 score ~−6.02 kcal/mol at bin score ~−6.0 kcal/mol (green in Figure 4B) was used for simulation. This pose is located near, but not in, the zinc-binding site of GluQRS, as shown in Figure 1C. Like the crystal binding site for Glu, this location presents a diverse group of residues, but unlike the crystal site, four of the five interacting residues, Ile162, Asn163, Arg165, and Tyr172, arise from a local sequence segment; thus, tertiary engagement of the ligand is limited at this location. Together with the fifth contact residue, Arg200, a total of ten bonding interactions is present in the docked structure as viewed in PoseEdit (Supplementary Figure S4). A locally high positive charge may attract the ligand to this vicinity: a mobile loop region following His164 includes Arg165 and Arg166, and extends to Ala171, the N-terminal residue of slightly distorted helix 7. The large phenol sidechain of Tyr172 lying between the crystal site and location 3 can contact a Glu ligand in either location. Figure 5D shows that the ligand is not associated with the protein during most of the simulation time, and even during the RMSD plateau at ~450 to ~650 nsec the ligand does not maintain close distances except for occasional charge interactions with Arg200. The ligand also occasionally approaches atoms of the long Arg190 sidechain which, like Tyr172, can make contact on either the crystal site or location 3. 

**Figure 6:**
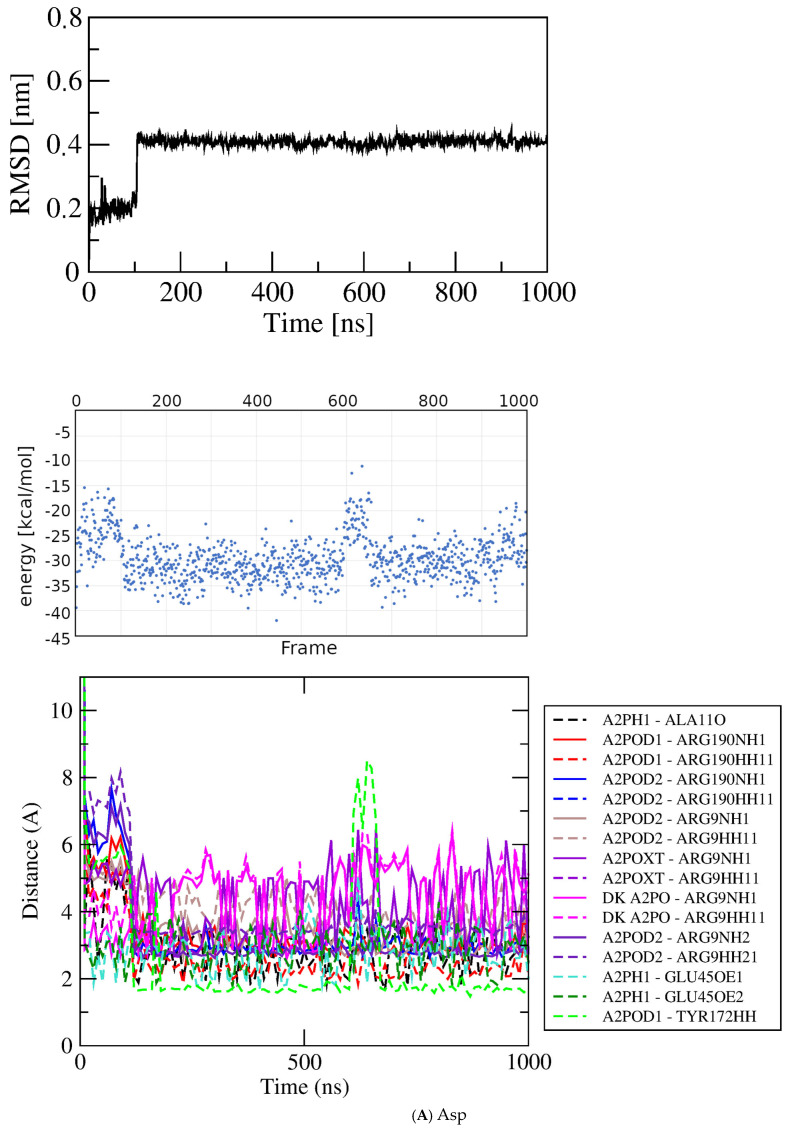

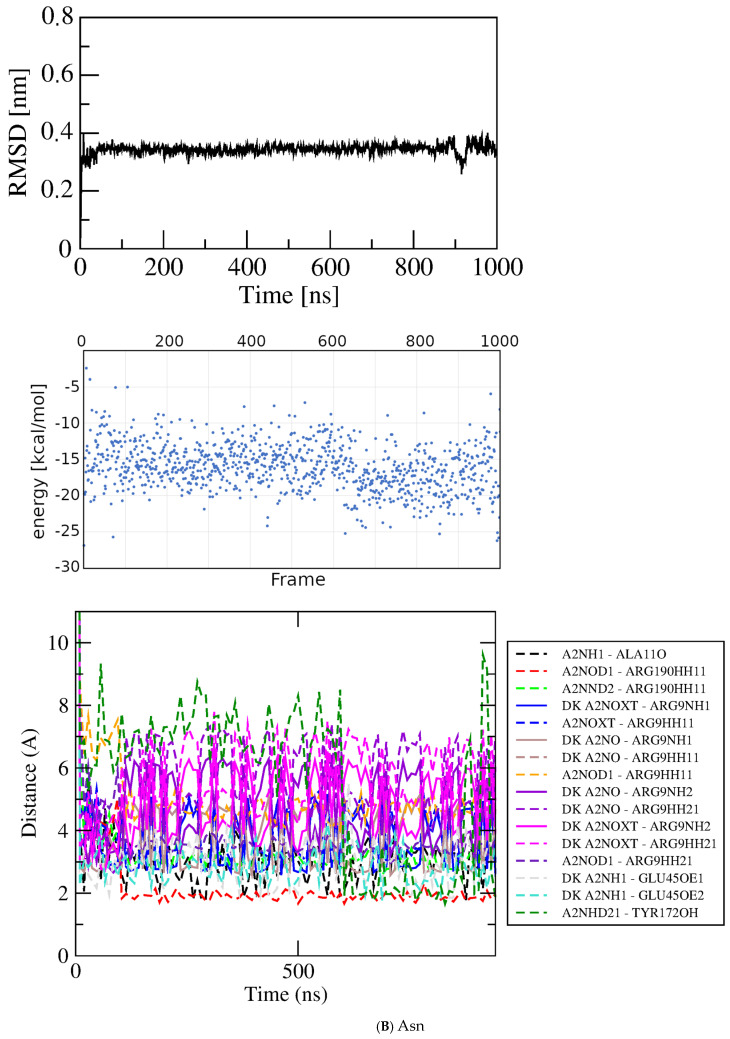

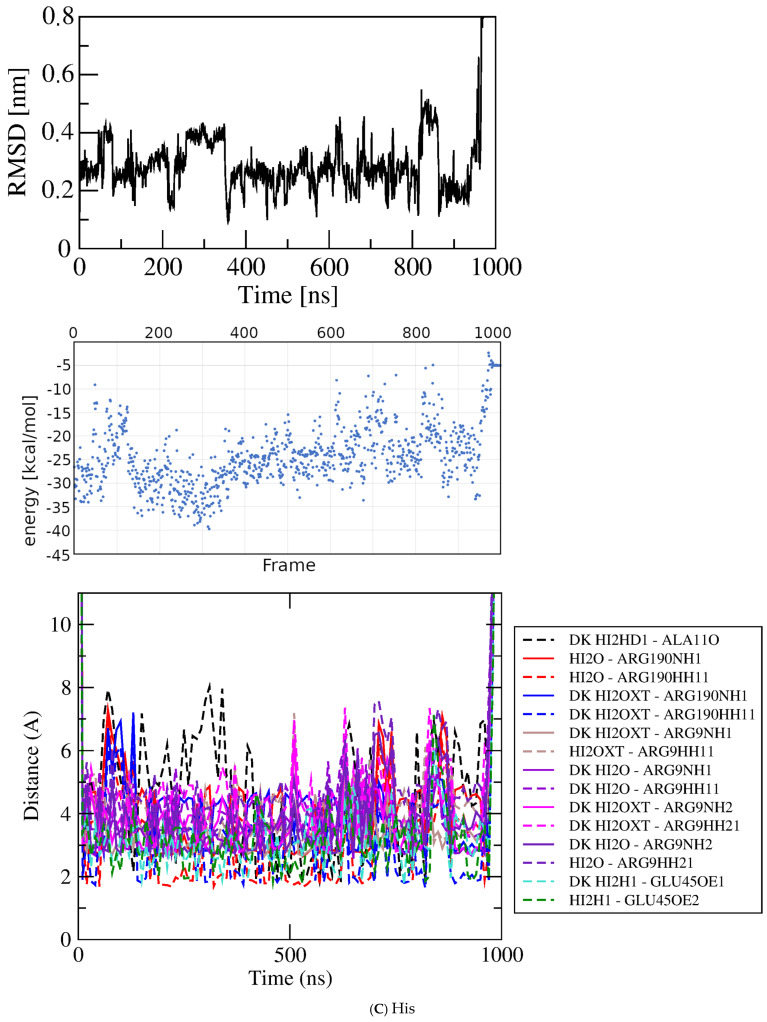

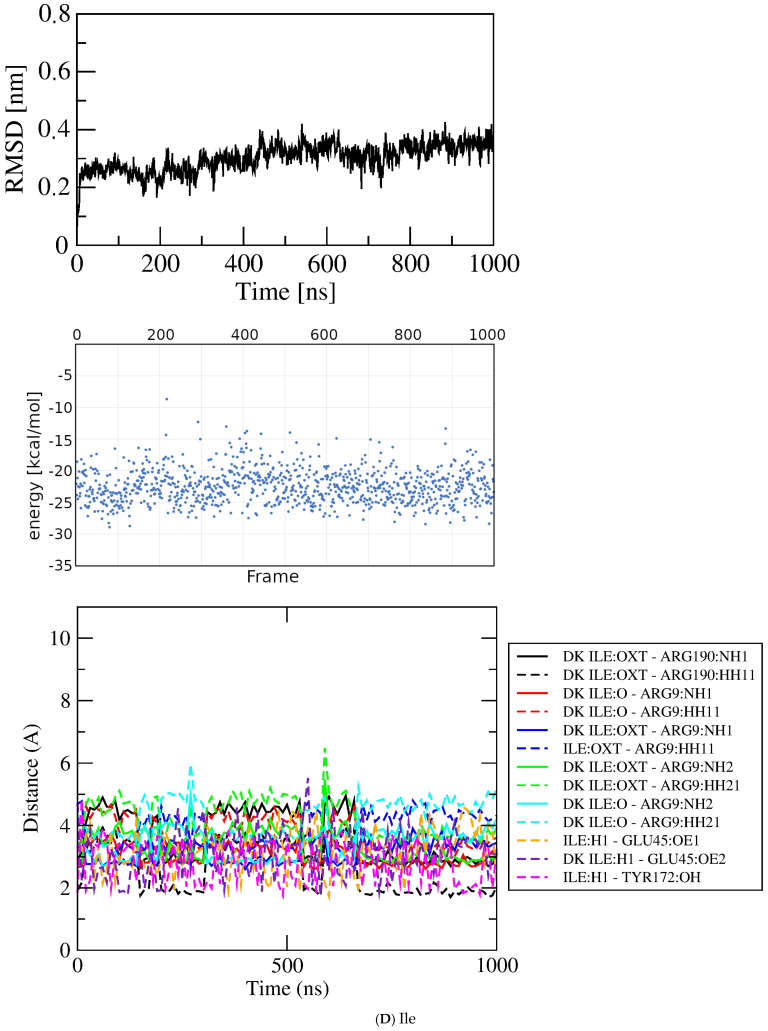

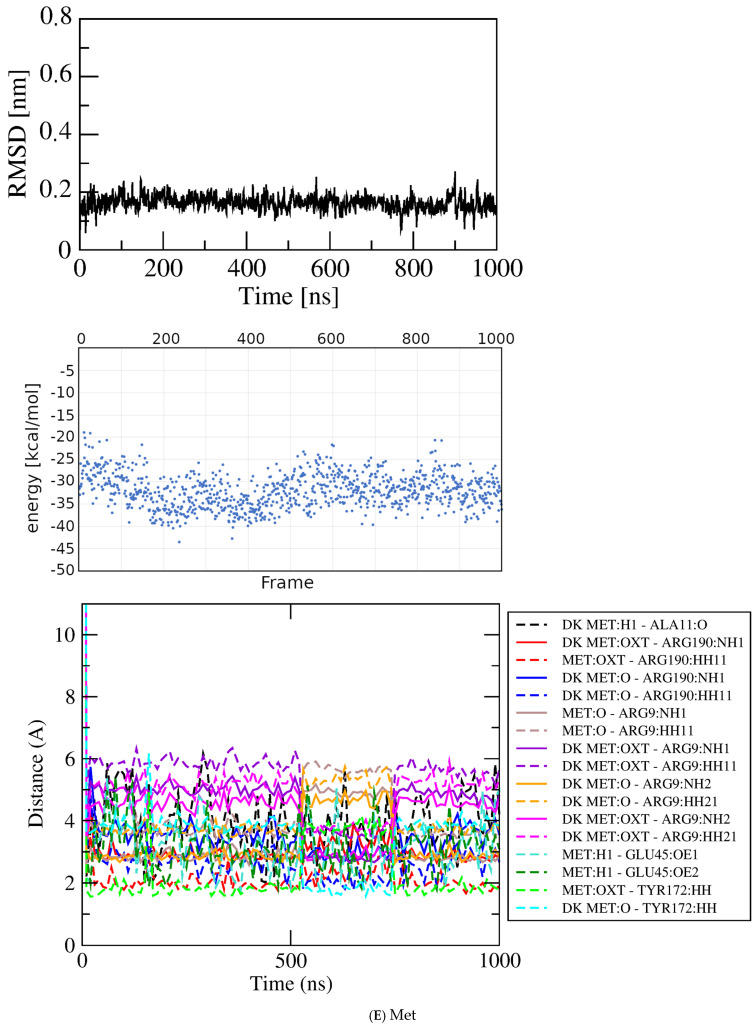
Results for docked Glu analogs. Top, RMSD; middle, GBSA energies; bottom, atomic contact distances (DK, contact present in docked structure). (**A**) Asp. (**B**) Asn. (**C**) His. (**D**) Ile. (**E**) Met.

GluQRS interactions with non-native ligands at the crystal site for L-Glu. MD simulations were conducted for GluQRS (PDB:4A91) with eleven non-native ligands, eight of them amino acids, plus S-adenosyl methionine (SAM), adenosine monophosphate (AMP), and cyclic (cAMP). Nine ligands docked exclusively in the L-Glu crystal binding site, and seven of those maintained association there during simulation, though not always continuously, as discussed below. Ligands that docked and stayed at the crystal site for all or much of one microsecond are listed in Table 1, and typical RMSD plots for each ligand are shown in Figure 6. L-glutamine (Gln) binds poorly to GluQRS in presence or absence of ATP without tRNA [14]. Docked poses of Gln at the crystal binding site for Glu were simulated but did not present persistent association and were not studied further.

Aspartic acid. Aspartic acid (Asp) may be considered of particular interest due to its close structural similarity to Glu and the functional relationship to GluQRS through its tRNA, which is the substrate for glutamylation of queuosine by GluQRS in the anticodon loop. GluQRS is reported not to aminoacylate Asp [14]. Asp is the only non-native ligand in Figure 4C with a bimodal distribution of pose scores like that of Glu at bin score ~−6.5 kcal/mol Figure 4B. The two Asp pose-energy groups in this bin are both at the crystal binding site for Glu, but they differ in the Asp conformation, with the weaker-energy group displaying a linear conformation like that of Glu, and the stronger group displaying a more compact conformation in which the sidechain carboxylate is closer to the alpha substituents due to rotation about Chi1. An initial docked pose for Asp in the linear conformation, with dock score −2.93 kcal/mol in Figure 4C, was chosen for simulation. The docked pose (PoseEdit, Supplementary Figure S5) has twelve bonding interactions to Asp from five of the six residues that provide the fourteen bonding interactions observed for crystalline Glu.

The RMSD plot (Figure 6A) displays two sharp changes at ~zero and ~115 nsec. Visual inspection of structures in VMD confirms that Asp remains associated at the Glu crystal site throughout the simulation, but with frequent changes in the details of bonding with its five partner residues, similarly to the observations with Glu. The initial shift to RMSD ~0.18 nm corresponds to loss of one bond with Ala11 and gain of one bond with Gly191. By 20 nsec, one contact residue contributing one interaction is lost. Thereafter, RMSD variability of up to ~0.1 nm during the first ~100 nsec reflects frequent changes in Asp orientation and frequent excursions to an alternate conformation like the one observed in the docked poses at stronger scores. As assessed by analyzing the dihedral angle Chi1 (atoms N-CA-CB-CG), already from zero time the ligand alternates frequently between the favored *trans* conformation (~180°; [22]) in which the alpha-carboxylate and the sidechain carboxylate are nearby, and the more extended or linear *gauche*+ (~60°). Thereafter, Chi1 wobbles around 180 +/− 10°. The sharp transition to RMSD ~0.4 nm at ~105 nsec corresponds to loss and then net gain of bonds to a maximum of sixteen, including regain of the residue lost in the earlier shift, Ala11, the first residue of the turn following strand one.

The *trans* conformation of Asp persists only upon establishment of a bond between its alpha-amino group and the backbone carbonyl oxygen of Ala11. Although Ala11 is nearby prior to forming this contact and comes within bonding distance occasionally during earlier frames, a momentary excursion of the adjacent Ala11–Pro12 peptide bond and a change in ligand conformation enable a persistent hydrogen bond between the carbonyl oxygen and the ligand amino group. Detailed examination of structures in the frames near the sharp transition reveals that the Ala11-Pro12 *cis* peptide bond omega (dihedral Ala11CA–C’–N–Pro12CA) wobbles from its average value of ~175° to its most extreme position, ~156°, in the frame immediately before Asp undergoes a flip of Chi1 (dihedral N-CA-CB-CG) that brings its alpha-amino group into position to bond with the Ala11 carbonyl oxygen (C=O - - - N-C distance ~2.8 Å). In the first 75 nsec, the ligand samples the linear conformation eight times; after 75 nsec the ligand does not sample the linear conformation at all, and Chi2 stops sampling extremes, remaining within the range ~−110 to −150. This new ligand conformation with contacts to the five residue partners is maintained for the remaining ~900 nsec, with the total number of bonding interactions fluctuating between fourteen and fifteen as seen in PoseEdit (Supplementary Figure S6).

These results suggest that the stochastic motions of the protein and ligand enable a mutual induced fit, as has been reported recently in other cases (see, e.g., [23]), that in the case of GluQRS/Asp leads to persistent association. This result recalls the result with Glu at the novel site discussed above, where slight unraveling at the N-terminus of helix seven limits the close approach of Tyr172 and terminates association of Glu. The results further suggest that Asp may indeed bind to GluQRS, a prediction that can be tested in future experiments to measure affinity and crystallize the complex. Confirmation of binding in the *trans* conformation would suggest that the propensity of Asp to adopt this conformation in the Glu binding site could be a factor that contributes to the inability, reported previously [14], of GluQRS to aminoacylate Asp. Extensive efforts to evaluate whether Glu also can adopt a conformation in the crystal binding site like that of *trans* Asp indicate that it does not do so.

Asparagine. Like Asp, asparagine (Asn) has a bimodal distribution of docked-pose scores, with one pose at a much weaker score (~−4.5 kcal/mol) than the main group at ~−6.5 kcal/mol. The weaker-scoring pose has a slightly more compact conformation due to a small deviation in Chi2 than the main group with linear conformation, but is not fully in the compact form seen with Asp. The linear docked structure selected from the main group for MD at the crystal binding site of Glu (PoseEdit, Supplementary Figure S6) shares some features with the docked structure of Glu and others with that of Asp. Like Glu, the Asn docked structure engages all six contact residues, including Ser13 that is absent from the docked contacts of Asp. The thirteen functional group interactions of Asn seen in PoseEdit include some of the twelve of Asp and fourteen of Glu, but uses them differently. The Asn alpha-carboxylate interacts with Arg9, Arg190, and Tyr172, whereas with Glu these residues interact with the sidechain carboxylate. Instead of engaging the alpha-amino group as for Asp and Glu, Ala11 joins Ser13 in interactions with the sidechain amide of Asn. The sidechain carbonyl oxygen of Asn has no interaction partner, unlike that of the alpha-carboxylate of Glu with Ser13.

The RMSD plot for Asn is shown in Figure 6B. Despite the seemingly promising docked structure, residue contacts with Tyr172 are lost in the first frame, resulting in a shift from zero to ~0.35 nm. Thereafter, the RMSD is remarkably stable with very small deviations, +/− ~0.02 nm. The three charged residues maintain their interactions with Asn throughout the simulation. By ~15 nsec an interaction with the Tyr172 amide replaces its sidechain interaction, enabled by some unraveling in the first turn of helix seven from its N-terminus at Ala171 that persists until ~100 nsec and is occasionally revisited throughout the simulation. By 60 nsec Ala11 replaces Tyr172 as the fourth contact residue, with the interaction alternating thereafter between the Ala11 carbonyl oxygen and the Tyr172 hydroxyl. Around 90 nsec the ligand undergoes a conformational change about Chi1 to adopt the *trans* conformer as Asp does around 100 nsec. However, for Asn there is no corresponding large shift in RMSD, contact residues, or interacting groups. Rather, the ligand conformation continues to shift back and forth throughout the simulation while the contact residues and interactions adapt, maintaining an average of ~twelve PoseEdit interactions and GBSA energy of ~−15.80 ± 3.11 kcal/mol. Although this energy value suggests that Asn has significantly weaker association than Asp or Glu with energies ~−30 kcal/mol, the overall pattern of the interactions in time and number of close distances is rather similar for Asp and Asn.

Histidine. The results for Asp and Asn suggested that the proximal part of the sidechain imidazole ring of histidine (His) might mimic the *trans* conformation of those ligands, perhaps recruiting alternate partners for the imido functional groups. A docked structure with AutoDock4 score ~−6.2 kcal/mol in Figure 4C was selected for simulation at the GluQRS crystal site for binding of Glu. Figure 6C shows the MD results for His binding. Despite the rather large deviations of RMSD, inspection of the structures in VMD confirms that the ligand remains associated with the protein during the entire one microsecond simulation. Like Asp and Asn, the ligand shifts frequently between extended and compact conformations. In addition to these shifts in conformation, and independently from them, the extended ligand orientation also flips by ~180 degrees while maintaining approximate alignment with the protein long axis. These motions account for the large RMSD values as well as their large deviations. As a result, the ligand rarely populates short distances to bonding functional groups, and remains anchored by a few charged-group interactions that persist at relatively long distances.

Isoleucine. The beta-branched isoleucine (Ile) sidechain places the gamma-methyl group in a position like the sidechain substituents of Glu, Asp, and Asn, but without the functional groups that provide their bonding capacity. The docked structure selected for simulation with Ile engages only the three charged residues plus Tyr 172, forming fifteen bonding interactions exclusively with the ligand alpha substituents (Supplementary Figure S4). Nevertheless, the RMSD results (Figure 6D) indicate a single plateau at ~0.25 nm rising gradually to ~0.35 nm and deviations +/− 0.1 nm throughout, and inspection of structures by VMD indicates that the ligand remains associated with the protein for the duration of the simulation. Those same four residues contribute the only contacts during the time course, with bonding interactions decreasing to twelve at 2 nsec then rising to and remaining at thirteen by ~300 nsec. The average energy in the plateau at ~440 to ~630 ns is –22.36 ± 2.66 kcal/mol.

Methionine. Methionine (Met) is a near-isostere of Glu, lacking only one sidechain oxygen, but with rather different polarity. The docked structure of Met (Supplementary Figure S5) shows the ligand linearly extended and positioned similarly as Glu, with eleven contacts to five of the six residues contacted by Glu in its crystal structure (all but Ser13), plus a novel hydrophobic interaction with Phe10. The RMSD plot for a one-microsecond simulation in Figure 6E shows an initial jump from zero to ~0.15 nm, with deviation +/− ~0.1 nm that persists for ~900 nsec until doubling to ~0.2 nm in the final 100 nsec. Inspection of structures in VMD confirms the ligand remains in an associated state during the entire simulation, although as for Glu, Asp, and Asn, the identities of contact residues and the numbers and nature of bonding interactions change. By 20 nsec, contacts with Phe10 and Ala11 are lost, and bonds persist with the three charged residues plus Tyr172. By 250 nsec Ala11 contact resumes. By 500 nsec Ala11 has been lost again, thereafter alternating with hydrophobic interactions with the methylene groups of the Arg190 sidechain; this condition is maintained through 980 nsec. Thus, although its sidechain is more hydrophobic, the behavior of Met during the simulation is similar to that of Glu in that it remains in a linear conformation at the same protein location with a constant set of contact residues while occasionally shifting its bonding interactions, including recruiting the new hydrophobic interaction. The GBSA energy in the plateau region at ~100 to ~750 nsec is −32.86 ± 3.74 kcal/mol.

SAM. The results with Met motivated the choice of S-adenosyl methionine (SAM), which contains in addition to the Met moiety the adenosyl moiety like the GluQRS reaction product Glu-AMP. The docked pose chosen for simulation with SAM shows its Met moiety situated very similarly as Glu at its crystal site, as for docked Met (Supplementary Figure S5). Five of the same six residues are engaged (except Ser13), making ten bonding interactions with the Met moiety of SAM compared to the thirteen with Glu in its crystal and fourteen in its docked structure. The bonding arrangements differ very slightly in the docked structures of free Met and the Met portion of SAM. In addition to the contacts with the Met moiety, the adenosyl moiety of the ligand contacts residues Pro12, His19, Ser22, and Leu194 that form a network of hydrophobic contacts with the Met sidechain and with some of the Met contact residues. This group of residues is on the upper side of the crystal binding site as viewed in the cartoon of Figure 1A. Three new hydrogen-bonding interactions from residue Asp193 and one pi-stacking interaction with His19 bring the total number of bonding interactions formed with SAM to fourteen. The AutoDock4 score of the docked pose is ~−6.7 kcal/mol.

This extraordinary tertiary engagement, represented by the colors identifying the structural locations of the interacting residues (Supplementary Figure S5), is lost progressively and not revisited as the partner numbers, identities, and bonding arrangements evolve during the simulation. The RMSD plot shows an undulating behavior centered at ~0.6 +/− ~0.1 nm over the entire one microsecond. Because of the large numbers of functional groups on the ligand, its interactions with the protein are described here with reference to the inferred bonding displayed in the PoseEdit structures (Supplementary Figure S7) for clarity, rather than with atomic-distance plots. The adenine ring itself remains engaged to only a limited extent in time and bonding interactions, whereas the ribose ring maintains hydrogen bonding with Asp193 throughout. Like the other ligands, SAM remains highly mobile and many of its interaction partners change frequently over the time course. By 20 nsec the residue partners with the Met moiety are reduced to three, and to one with the adenosyl moiety, while the total number of bonding interactions counted by PoseEdit decreases to fifteen. These four partner residues are maintained through ~50 nsec while the total number of bonding interactions fluctuates. By 120 nsec four additional hydrophobic contacts are recruited to the adenine ring but are lost by 450 nsec. Thereafter only charged groups remain engaged with the ligand, including newly recruited Lys231 that forms one predicted hydrogen bond with the adenine exocyclic amino group. Bonding interactions reach twelve at 700 nsec before declining to seven at 800 nsec. By the last PoseEdit time point even the charge interactions with Arg9 and Arg190 are lost. Despite the loss of most interactions and the undulating RMSD, the calculated GBSA energy over the entire simulation is ~−33.5 with a maximum value of ~−38.0 kcal/mol in the 450–750 nsec plateau, presumably reflecting the strong contributions of the charged-group interactions. Thus, even the seemingly promising docked pose for SAM apparently does not lead to a stable associated state.

Cyclic AMP. Cyclic AMP (cAMP) was chosen as a ligand for the structural (but not electronic) resemblance of its phosphodiester linkage to the phosphoanhydride moiety of Glu-AMP. The docked location is at the crystal site for Glu binding with AutoDock4 score ~−6.3 kcal/mol, although only two contact residues each form one hydrogen bond with phosphate oxygens; two additional residues make hydrophobic contacts with the adenine ring. In contrast to the results with SAM, the docked structure chosen for simulation of cAMP has relatively few contacts and interactions, but their numbers grow progressively over the course of the simulation. The RMSD plot shows that a stable plateau is reached before 100 nsec and is maintained with ~0.05 nm deviations throughout the simulation. As with SAM, the PoseEdit interactions are cited here for clarity (Supplementary Figure S7), rather than atomic distances. At 5 nsec the number of bonding interactions and hydrophobic contacts rises to three each. At 200 nsec two hydrophobic contacts form, and six bonding interactions with recruitment of Glu17. At 438 nsec hydrophobic contacts are four, and bonding interactions are seven with addition of Arg51; at 650 nsec hydrophobic contacts are three, bonding interactions are nine with recruitment of His19, loss of Arg51, and changes of bonding details; at 850 nsec, hydrophobic contacts are three, bonding interactions are eleven with unchanged partners but altered bonding details. Calculated GBSA energy reaches its value of maximum stability during the plateau at ~500 to ~750 nsec at ~−57.5 kcal/mol; the average GBSA energy for the entire simulation is ~−56.2 kcal/mol. Comparison of the SAM and cAMP results thus shows that two docked structures of similar AutoDock scores can diverge significantly: a seemingly excellent one can progress to a barely interacting one, whereas a seemingly poor one can evolve to a highly interacting one.

AMP. Adenosyl monophosphate (AMP) was simulated from structures docked at three distinct locations (Supplementary Figure S9): at the modeled site for ATP/AMP [14]; at the crystal binding site for Glu; and at novel location 2 near but not in the crystal binding site of Glu (Figure 1C). At the modeled site for ATP/AMP, the docked pose features bonding interactions with Gly21, the only residue bonding with the adenosyl moiety; Ala221 and Ser230 that interact with the ribose moiety; and Lys228, which interacts with the phosphate group. The RMSD plot displays no plateau regions, increasing gradually over the time course to ~0.4 +/− 0.15 nm. By 900 nsec two new interactions are recruited, with Ala192 and Arg147. As with the other simulations in this work, the ligand maintains mobility in the binding location, and interacting partners often change during the time course, although they may be preserved in any specific time point captured by PoseEdit. The average GBSA energy over the simulation is −23.2 kcal/mol.

At the crystal binding site for Glu, AMP engages four of the six residues engaged by Glu in its crystal structure, the phosphate group with Arg9, Tyr172, and Arg190, and the ribose with Ser13. These interactions do not persist although the ligand remains associated as the RMSD increases gradually over the first ~300 nsec to ~1 nm with deviations less than ~0.2 nm before a dissociation event. Thereafter, reassociation is observed around ~550 nsec to a new location with plateau at ~0.3 +/− 0.25 nm that persists until ~850 nsec before dissociating again. This location is closer to the zinc-binding site, where the ligand recruits new residues Arg105, His124, and Arg166 briefly. By the end of this plateau period only Arg105 and Ala106 make bonding interactions with the ligand, i.e., loss of tertiary engagement. The GBSA energy quickly diminishes from ~−40 kcal/mol in the first ~50 nsec, with an average value over the simulation of −14.25 +/−10.55 kcal/mol, reaching the most favorable value during the 660–750 nsec plateau region of −23.33 +/− 6.48 kcal/mol. The large deviations of the GBSA data, together with the RMSD results, indicate that this simulation does not reach a stable condition; therefore, the frequencies of atomic distances were not measured.

At novel location 2 the ligand engages Thr143 with the adenosyl moiety, Lys211 with the phosphate, and Val212 and Asp214 with the ribose. Three of these four residues arise from a short linear segment of the sequence, representing poor tertiary engagement at this location. Indeed, the ligand soon dissociates as seen in an early RMSD spike, then returns to a location near the one visited at ~600 nsec in the AMP simulation that started from the crystal site for Glu. This location is maintained during an RMSD plateau at ~3.5 +/− 0.5 nm from ~100 to ~500 nsec. By 700 nsec the RMSD stabilizes for ~100 nsec at ~3 +/− 0.2 nm as the ligand has recruited additional interacting partners, but these are all from linear chain segments. Following another dissociation the ligand returns to yet another location nearby the previous one, with similar RMSD values. As with the other simulations in this work, the ligand maintains mobility in each location, and the interacting partners often change during the time course including during the plateaus. The average GBSA energy over the simulation is −14.54 +/−9.12 kcal/mol, reaching the most favorable value of −23.38 +/− 6.37 kcal/mol during the 660–750 nsec plateau region. As in the case of AMP at the Glu crystal site, the frequencies of atomic distances were not measured.

Intrinsic specificity ratio. Supplementary Figure S9 displays histograms of the GBSA energies calculated for all ligands during the entire one-microsecond simulations, prepared from the dot plots for each respective ligand simulation examined here. Table 1 summarizes the average GBSA energies and standard deviations during the entire one-microsecond simulations and during selected RMSD plateau regions. These data were used to compute the intrinsic specificity ratio, ISR, for each ligand according to the method of Wang et al. [3]. Those authors had used docking scores obtained by allowing AutoDock to run to convergence in order to acquire a comprehensive distribution of energies. The GBSA energies used here, calculated over the entirety of each one-microsecond simulation, thus represent a similarly comprehensive distribution, although the values are in a different range because of the manner of calculation.

The ISR value for a given protein–ligand pair is the ratio δE/ΔE, where δE is calculated as the strongest energy of the distribution minus the mean energy, and ΔE is the value of one standard deviation of the distribution. The ratio thus represents the degree to which the strongest-energy pose is separated in energy from the vast majority of poses. As such it quantifies goodness of fit of one ligand pose relative to other poses. This measure is not the same as specificity relative to other ligands or targets, and ISR values cannot be compared with specificity values calculated as ΔΔG for pairs of protein–ligand interactions. Once experimental ΔG values are acquired in future work with GluQRS and its ligands studied here the two measures can be compared. In any case they do not quantify the same property, a fact independent of definitions that does not diminish the potential utility of the comparison.

The ISR values calculated for each protein–ligand pair simulated in this work are given in Table 1 for each respective GBSA histogram (Supplementary Figure S8). The values are uniformly small, even for the native ligand Glu, and not constant over replicate simulations, consistent with the wide variations seen in this work within, between, and among simulations. Glu is one of the few ligands studied here with a maximum docking score that is separated from the main distribution, and only a subset of the Glu simulations display a separation in GBSA maximum-energy value from the mean. The only other ligands that present a maximum-energy value separated from the main distribution are Asp, Met, and AMP. In all cases the population of maximum-energy values is also uniformly small, suggesting these values belong to the tail of the main distribution rather than being separate from it. Consistent with this suggestion, the few separated maximum-energy values observed here for some ligands are hardly distant from the main distribution, considerably closer than those found for inhibitors of the Cox-2 protein by Wang et al. [3] using docking scores. A weak preference could be consistent with the rather open nature of the GluQRS binding site that must also accommodate ATP as well as Glu, or Glu-AMP plus the Q-base anticodon region.

**Figure 7:**
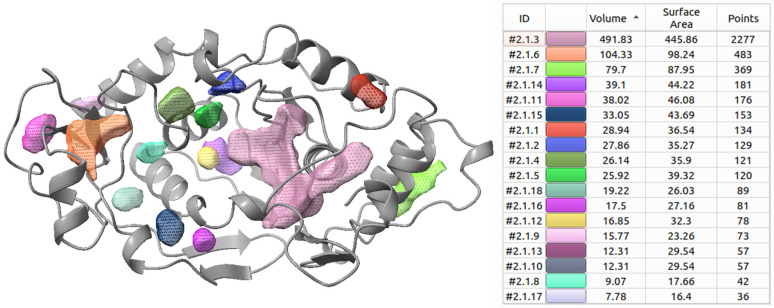
Predicted cryptic sites. The free online tool CryptoSite [19] was used to predict cryptic sites using as input file PDB:4A91 chain A. **Left**. Secondary structure cartoon of protein crystal structure (grey) and predicted cryptic sites (colored mesh). The orientation of the protein is 90° clockwise rotation from the view in Figure 1A, with zinc binding site at left. **Right**. Key to colored volumes (Å^3^) shown at left. The colors and ID numbers (#) are arbitrary and serve only to identify each volume uniquely. For reference, the volume of one water molecule is approximately 29.5 Å^3^.

Comparison with predicted cryptic binding sites. The free online tool CryptoSite was used to predict unoccupied volumes that might serve as ligand-binding locations within GluQRS (PDB:4A91 chain A) after removal of the crystal ligands Glu and zinc. The results are shown in Figure 7. Volumes smaller than one water molecule are included; these might coalesce with others nearby in presence of a ligand or under dynamics (the cryptosite calculation is static), but they are not analyzed further here. Six volumes larger than a water molecule are predicted, only three of which (~80, 104, and 492 Å^3^) are large enough to accommodate any free amino acid. The smallest of the three volumes can accommodate only the smallest amino acid, glycine. The next larger volume includes the crystal binding site for zinc, and can accommodate alanine (Ala) or serine (Ser). The largest of the three volumes can accommodate all amino acids and is more than twice as large as the largest, tryptophan (Trp). The location of the Trp novel site is partially within this volume; the Trp ligand does not maintain association there after ~200 nsec.

The largest predicted volume extends to and includes a small part of the location that in the crystal structure is occupied by the sidechain carboxylate of bound Glu (i.e., the wrong end of Glu for aminoacylation with the gamma phosphate of ATP). The volume is not large enough to accommodate ATP (~750 Å^3^), the second natural substrate of GluQRS, consistent with the report that ATP does not extend all the way into the binding site [14], but it is more than large enough to accommodate all of Glu and the AMP part of ATP. AMP occupies ~300 Å^3^, suggesting the site can accommodate Glu-AMP following aminoacylation. The third substrate of GluQRS, tRNAAsp, must also bind and present its anticodon loop, or at least the everted queuosine base, to accept the glutamyl moiety from aminoacyl-AMP. More detailed studies will be required to evaluate how these substrates are accommodated simultaneously and in reactive proximity. The space available at this location is considerably expanded in the course of MD simulation of the unliganded protein reported above, largely due to motion of the long irregularly-structured loop at residues 223–242.

## 3. Discussion

The work reported here documents significant departures from contemporary understanding of protein–ligand complexes when viewed in long, atomistic MD simulations. Many different poses for Glu are returned by a docking algorithm at distinct locations on the protein, with sometimes overlapping score ranges, and even the best of these does not remain associated in a fixed manner during simulations starting from the docked location, nor even when simulated from the crystal structure without docking. Instead, the numbers and identities of contact residues and bonding partners change continually, with some initial contacts never revisited and new contacts recruited, all while the ligand is conformationally mobile in its original location. The results suggest that ligand and protein cooperate dynamically in forming the observed interactions. Analogs of the native ligand for which there is no experimental evidence of interaction displayed comparably effective durations of association and energies in MD, and similar frequent shifts of functional-group interactions. All predictions of potential interaction based on the present results should be tested directly in ligand-binding experiments. The possibility that Asp, Asn, or cAMP may bind to GluQRS also motivates a search for evidence that could imply a metabolic connection.

The molecular origins of ligand-binding affinity and specificity are typically considered to be the bonds between the partners. This view inspires protein-targeted drug design, in which bonding and steric fit are optimized between a disease-related protein and candidate drugs or drug fragments from a compound library. Typical steps are similar to those used in the present work, i.e., docking (including ensemble docking [24,25,26] not used here), followed by MD simulations and energy calculations. Interactions in the complex are then adjusted in efforts to optimize affinity for the chosen target and specificity vs. other potential target proteins present in vivo. This approach has had some notable success, but one might well ask why the successes appear to be so few. One reason can be related to results reported for other systems showing that ΔG can be quite large and negative but that ΔH need not be, and can even be positive (see, e.g., the example of Cro/DNA binding cited by Klotz [27]). The net effect of bond-making and bond-breaking anywhere in the system, and not necessarily in the protein/ligand bonds, shows up in the sign and magnitude of ΔH. Thus, even negative values of ΔH need not reflect the observed bonding interactions. These facts indicate that the bonds between the partners are not “holding” the complex together, nor determining its specificity. Rather it is the overall free-energy lowering of the system in the bound state relative to the free state that defines ligand affinity. The abstractness of this conclusion appears unsatisfying against the seductive visual nature of partner bond interactions.

It is customary, but not strictly correct, to think of preferred ligands as having better steric and functional-group complementarity to maximize non-covalent bonding in the bound state. In this context, ligand mobility while associated might at first glance seem to be a sign of poor binding. However, it could instead represent a favorable entropic contribution to the overall free energy change upon binding, as well as an opportunity to achieve a net improvement of interactions and energies by mutual adaptation. Although bonding complementarity is relevant for binding selectivity in systems that are highly preorganized, where it can confer great predictive power, this principle breaks down in adaptable systems that undergo reorganization of ligand and/or target upon interaction, as suggested for the cases studied here. Reorganization is a hallmark of many biological interactions, with consequently profound effects upon affinities and specificities [8]. If there is a reorganization cost anywhere in the system for achieving optimal bonding complementarity, then that cost could exceed the benefit of improved bonding, perhaps leading to reversal of preference among ligands.

This analysis of selectivity may be relevant for understanding the small ISR values found here. GBSA system energies can be expected to give different results from the docking scores used in the original ISR approach, because the protein is treated as static in docking but in MD it is mobile and can adjust to the ligand—and with it, as seen here. It is not unexpected that this adaptability may result in a continuum of energies among locations and conformations, i.e., a less rough potential-energy landscape for interaction and a smaller numerator in the ISR calculation. The ISR approach is likely to be valuable for identifying differential binding among ligand candidates depending on the degree of preorganization in each system. Large ISR values based on GBSA system energies may indicate a higher degree of preorganization, and thus higher likelihood of success at improving binding by optimizing bonding interactions.

The results raise many questions including, What exactly constitutes binding? When do docking or MD results qualify as binding? What standards should be used to judge successful prediction of binding that is critical for contemporary approaches to protein-targeted drug development? Cocrystallization with a known ligand is ordinarily assumed to represent binding. Docking of a ligand in its known pose at its crystal site, as in some of the simulations here with native ligand Glu, would ordinarily be considered to represent binding at that site as well. Simulations operate on very different scales of time, space, and energy than the two main ways we have of viewing the bound state, NMR and crystallography. In this report the term “binding” was not used except to describe the known crystal structure of GluQRS with bound Glu. This choice was made to avoid referring to the results of docking or simulation as binding, rather asserting that those results represent potential associated states, and interactions that may occur within them.

Taken together the results of the present work suggest distinct views of what constitutes binding that depend on how you look. With adequate electron density, crystal structures can resolve associated species and map their bonding contacts, even for water or crystallization additives that are not typically considered to be ligands and whose interactions may be undetectable by other methods. This common observation suggests that localization is possible even for species that may occupy a broad and shallow potential-energy minimum, and leads to the question, What potential-energy surface is required to see binding in structures? In MD? In experiments? From an experimental viewpoint it is not unreasonable to say there is no such thing as no binding, only binding that is too weak to observe with the chosen concentrations, solution conditions, and methods. Broad, shallow potential-energy surfaces presumably correlate with binding that is weak and not well-limited to a unique location or set of interactions. But what potential-energy surfaces correlate with binding? How deep? How narrow? In truth we do not have a reliable way, experimentally, computationally, or even theoretically, to distinguish binding from non-binding, or to define a cutoff point between them, nor do we know much about the dynamic properties of the bound state of ligand and target. NMR has the power to resolve ligand and target dynamics in the bound state (see, e.g., [23,28,29]), although relatively few reports have done so as of yet. NMR approaches may be able to characterize intermediate states that may exist in the liminal domain between bound and free. The present results might stimulate new investigations along these lines.

## 4. Materials and Methods

The crystal structure of *E. coli* glutamyl-queuosine tRNAAsp synthetase (GluQRS, UniProt P27305), containing GluQRS with Glu and a zinc ion bound (PDB:4A91) was selected as the starting structure for this work. The GluQRS PDB structure 4A91 lacks electron density and modeled structure for the backbone and sidechains of residues Asn233 and His234. The protein structure was used for docking without repairing this small chain break, as this choice was considered less perturbing than an artificial connection because these two residues are at the center of a twenty-residue irregularly structured chain segment (Asn223 to Asp242 inclusive). Furthermore, the docking results were used only qualitatively to identify potential binding locations as judged by human knowledge of true ligand-binding interactions. For MD simulations the gap was replaced as indicated either by two residues modeled in YASARA (version 18.42.4S) as a loop, or by GROMACS (version 2021.4) with an artificial joint between the termini at residues 232 and 235.

All ions including zinc, water, and other compounds present in PDB:4A91 were removed from the structure file, and hydrogen atoms were added. In some simulations as indicated the zinc ion was replaced into its crystal site. Amino acid ligands were built in YASARA (version 18.42.4S) and minimized by a standard protocol [30]. For histidine only the protonated ring structure (proton at Nδ atom) was used. Force-field parameters for amino acid residues as zwitterions were derived for the alpha-amino and alpha-carboxylate groups in aqueous solution. Side-chain parameters were taken from AMBER99SB [31]. Molecules were optimized ab initio at the HF/6-31G level of theory with Gaussian09. Electrostatic potentials (ESPs) for residues were calculated by the Merz–Singh–Kollman scheme [32]. Initial atomic charges were assigned by fitting to the electrostatic potential calculated at the points selected according to the scheme. As fixed-charge forcefields do not account for polarizability, to mitigate spurious sensitivity of ESP fitting to molecular conformation, restrained electrostatic potential (RESP) was employed to restrain the polarization of buried atoms as described in Wang et al. [33]. Alpha-amino and alpha-carboxylate charges were set for the following residue groups: positively charged (Arg, Lys based on Lys), Pro, negatively charged (Asp, Glu based on Glu), and other (based on Ala). Existing Amber99 force fields were modified to account for the zwitterionic charges and correct numbers of hydrogens on amino acid ligands.

To assess the impact of removal of the Zinc ion on the tertiary structure and active site an additional structure was prepared with a zinc ion bound and with both zinc and Glu bound (documented in Supplementary Figure S10). To analyze the difference in stability between the protein with Zn and without, three repetitions of MD simulation were initiated in GROMACS (version 2021.4). Force field parameters for the coordination of the Zn^2+^ ion by the cysteine residues were obtained from the literature [34]. The charges on Tyr115 were modified for a protonated (i.e., neutral) hydroxyl group, as this is the most likely state for interaction with the Zn ion, as it is not involved in electron transfer in GluQRS [35,36]. The charge on the oxygen atom was decreased by 0.1 and the charge on the Cζ carbon atom was increased by 0.1.

The tertiary structure of GluQRS was stable in all MD simulations, reaching the same RMSD plateau, and comparable RMSF values, for the apo protein with and without Zn at the end of the simulations. RMSD, RMSF, and structure comparison between Zn-bound and Zn-free proteins are reported in Supplementary Figure S10. As discussed in Results, under concentrations observed in cells and in biochemical assays the protein can exist with bound Glu and without bound Zn ion [17,18]; thus, all other simulations reported here were conducted without bound Zn ion.

Force-field parameters for SAM were derived as described in [37]. Parameters for AMP were generated using acpype/Antechamber (Webserver version 23-01-24) [38]. Parameters for cyclic AMP were derived from R.E.DD.B, a database of RESP and ESP atomic charges and force field libraries for small molecules and molecular fragments [39]. In the process of simulation preparation, existing topology files were modified to include these parameters.

Global docking with AutoDock4, a free open-source software that uses a Lamarckian genetic algorithm [40], was carried out for each protein with each ligand in turn. In AutoDock the protein is treated as rigid, the ligand as unconstrained. The maximum number of energy evaluations was set to 2.5 million iterations for docking all ligands. An energy evaluation refers to the AutoDock scoring function for each ligand pose, i.e., the conformation at its current position and orientation in the grid-based receptor map, based on RMSD ≤ 2 Å. The AutoDock convergence criterion (not used here; Wang et al. [3]) is when the population of candidate solutions (poses) has clustered to the point where the average energy and RMSD of solutions do not improve in a set number of generations. One hundred poses with calculated energy scores were returned as a histogram for each protein–ligand pair. Their structures were screened manually as described in Results to select poses for MD based on features expected for known ligand-binding interactions.

Each selected pose was prepared for MD in explicit water (TIP3P; [41]) using GROMACS (version 2021.4). Structures were placed in a cubic box extended in each direction by 1 nm from the protein. Periodic boundary conditions were used with temperature 300 K, pressure 1 bar, and addition of Na^+^ or Cl^−^ ions as needed to achieve charge neutrality. The cutoff for long-range electrostatics used the Verlet Particle Mesh Ewald scheme. The number of replicas was one to three (Table 1). After energy minimization and short equilibration in NVT/NPT conditions (constant number of particles, volume, and temperature; constant number of particles, pressure, and temperature) MD simulations were initiated using standard parameters in NPT ensemble, modified Amber99 force field, with modified Berendsen thermostat (v-rescale, [42]) to keep temperature constant and Parrinello–Rahman barostat [43] for pressure control. Simulations were checked after ~100 nsec for maintenance of the ligand in an associated state by evaluating the RMSD relative to the initial structure. RMSD is measured over protein alpha carbon atoms and all atoms of the ligand including hydrogens, with the reference being the structure at the end of the equilibration period. Translating and rotating motions of the protein were removed by fitting each snapshot of the simulation onto the initial protein backbone.

MD calculations were run in the Princeton Research Computing resources center at Princeton University. Visualization of structures in MD trajectories used VMD (version 1.9.3) [44] and visualization of interactions used the online tool PoseEdit (https://proteins.plus/ from Universität Hamburg, accessed on 1 June 2025). PoseEdit indicates a hydrogen bond by a blue dashed line when two suitable atoms are within 1.9 +/− 0.5 Å and angle ≥ 120°, and a pink line when charged groups are involved. Note that the PoseEdit views are optimized to display the three-dimensional interactions in two dimensions with minimal mutual visual interference. As a result, they do not reflect the relative locations of residues surrounding the ligand; all structural descriptions in this work were verified in VMD.

Pose scores from docking were calculated with AutoDock4 [45], which considers interactions in vacuum. System energies were calculated with MM/GBSA (generalized Born surface area, [46]) using the gmx_MMPBSA (version 1.5x) tool for free energy calculation with GROMACS (version 2021.4) [47], which uses an empirical scoring function that also accounts for solvent; it calculates the protein and ligand separately and together to generate the difference between the associated and free states. The structure of the associated state at each plateau region of the RMSD plot as defined here was examined manually in VMD to determine if that plateau represents the ligand at a single location on the protein and to examine all interactions and conformations of both protein and ligand. System energies were calculated during the entire simulation times using MM/GBSA (version 1.5x) as well as during the plateau regions identified in Table 1. Each plateau was identified by visual analysis with the selection criterion being RMSD oscillations lower than +/− 0.2 nm from the mean in each region.

The intrinsic specificity ratio, ISR, for each ligand was calculated by an adaptation of the method of Wang et al. [3], who used docking scores obtained by allowing AutoDock to run to convergence; this work used GBSA system energies [10]. The ISR value for a given protein–ligand pair is the ratio δE/ΔE, where δE is calculated as the strongest energy of the distribution minus the mean energy, and ΔE is calculated as one standard deviation of the distribution. The very recent adaptation by Wang’s group to include system entropy [4] was not implemented here.

The free online tool CryptoSite (version main.f8471b9) [19] was used to predict cryptic binding sites using as input file PDB:4A91 chain A. Molecular graphics and analyses of the outputs were performed with UCSF ChimeraX (version 1.9) [48].

## Data Availability

Data are available upon request.

## Supplementary Materials

The following supporting information can be downloaded at: https://www.mdpi.com/article/10.3390/molecules30244678/s1, The Supplementary Materials include ten figures: Figure S1. Structures of Queuosine, Glu-AMP, and Glo-AMP. Figure S2. RMSF plots and heat maps. Figure S3. Histograms of atomic contact distances. Figure S4. PoseEdit views of all ten docked structures of Glu. Figure S5. PoseEdit views of all analogs as docked. Figure S6. PoseEdit views of conformational changes of Asp. Figure S7. PoseEdit views of SAM and cAMP. Figure S8. Histograms of GBSA system energies. Figure S9. Gallery of results for AMP: RMSD plots, GBSA energies, and PoseEdit views. Figure S10. Comparison of MD results with and without bound zinc: RMSD, RMSF, and structural overlays.
