# Supplementary material for: An MD View of Ligand Binding
Source: Molecules. 2025 Dec 6;30(24):4678. doi: 10.3390/molecules30244678 (PMC12736043; doi:10.3390/molecules30244678)
Supplement: Supplementary file 1 [file molecules-30-04678-s001.zip › Supplemental Figure S10 Comparison of MD results with and without bound zinc RMSD, RMSF, and structural overlays.pdf]

Supplemental Figure S10. Comparison of MD results with and without bound zinc: RMSD, RMSF, and structural overlays.

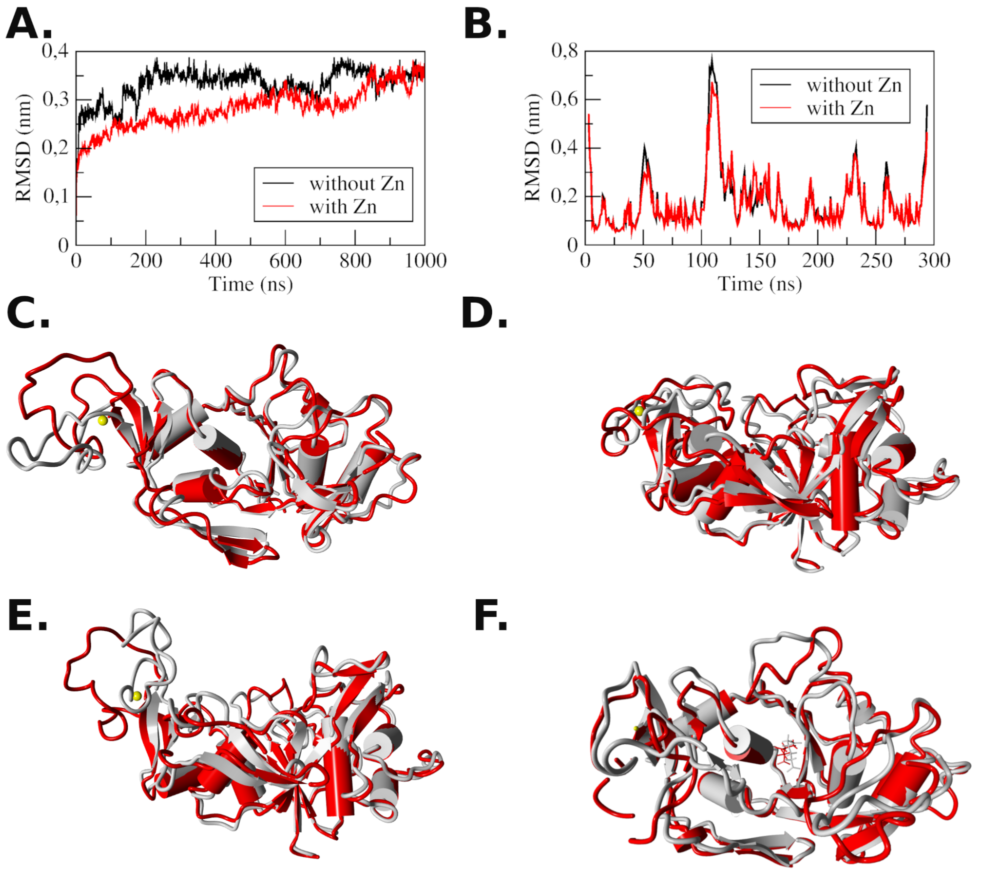

Supplemental Figure 10. Comparison of simulation results in presence and absence of zinc ion. Each panel compares results with Zn and without Zn. **A. RMSD of C-alpha atoms averaged over three independent simulations of GluQRS.** RMSD is slightly larger in absence of Zn initially but converges with the Zn-containing protein by end of the simulation. **B. RMSF of C-alpha atoms of each residue in GluQRS in absence of Glu.** The largest fluctuations for both proteins are in the region of loop 101-130 that includes all four Zn-coordinating residues. This region has a disordered conformation in both proteins (see left side of panels C and E). The fluctuations in the remaining parts of the protein are the same with and without Zn, indicating that its absence is not communicated throughout the protein, nor 'felt' in any other particular region. **C. Overlay of aligned structures after 100 nsec simulation in absence of Glu.** Result from the first of three replicate simulations that illustrate the overall fold of the protein is maintained and the mobile regions are confined to loops (with Zn: red protein chain, yellow Zn ion; without Zn: grey protein chain). **D. A rotated view from the second replicate simulation.** As in panel C, the overall fold is maintained and mobile regions are confined to loops. **E. A rotated view from the third replicate simulation.** As in panel C, the overall fold is maintained and mobile regions are confined to loops. **F. Overlay of aligned structures after 100 nsec simulation in presence of Glu.** A rotated view is shown to visualize Glu in location 1 as defined in Figure 1C. As in panels C, D, and E, the overall fold is maintained and mobile regions are confined to loops, and the location of Glu is the same in presence and absence of Zn.
