# Supplementary material for: An MD View of Ligand Binding
Source: Molecules. 2025 Dec 6;30(24):4678. doi: 10.3390/molecules30244678 (PMC12736043; doi:10.3390/molecules30244678)
Supplement: Supplementary file 1 [file molecules-30-04678-s001.zip › Supplemental Figure S2 RMSFs and heat maps.pdf]

Supplemental Figure S2 RMSFs and VMD heat maps. Left, RMSFs calculated over entire 1000nsec. Right, protein orientation is that of Figure 1. Red-white-blue, less mobile to more mobile. Each mobile region is identified by a labeled alpha carbon in the top panel. ***will be moved to correct place in text, and renumbered if needed.***

GluQRS peptide bond

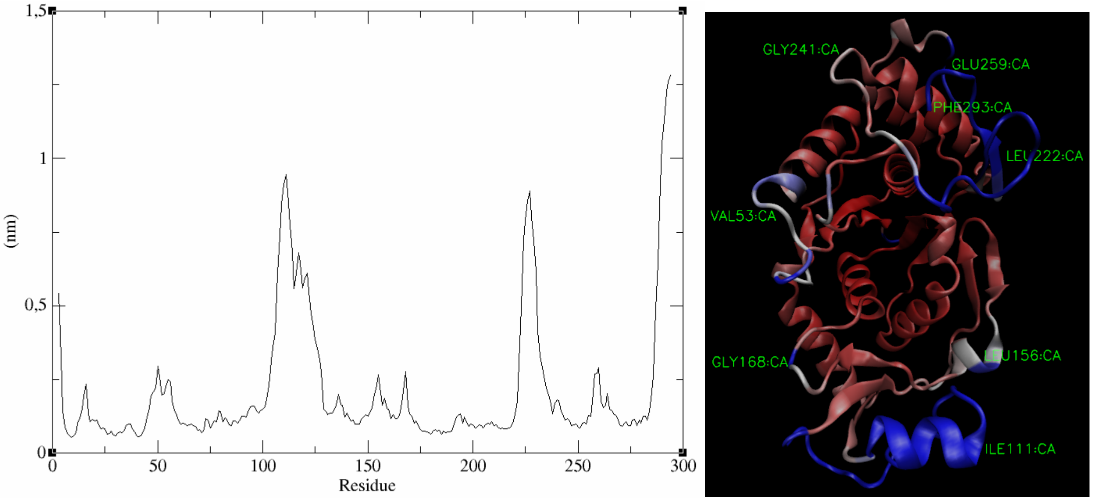

GluQRS peptide bond repeat

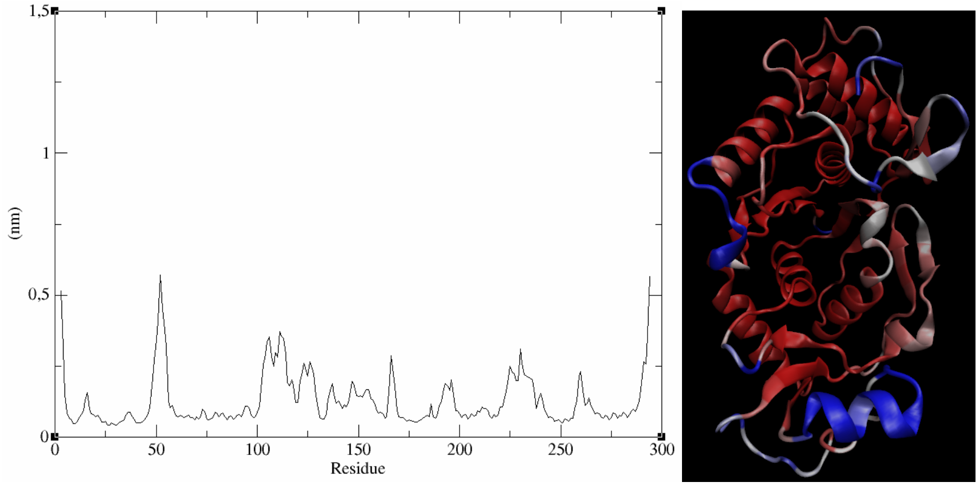

GluQRS modeled residues

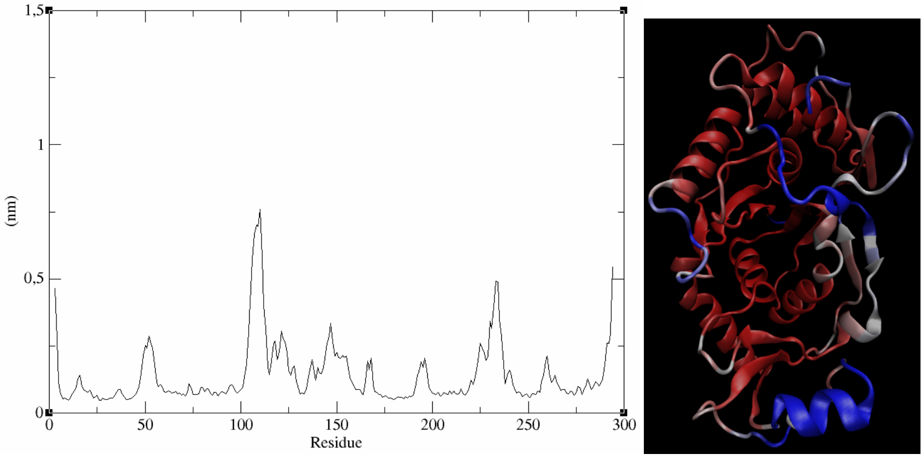

GluQRS/Glu peptide bond

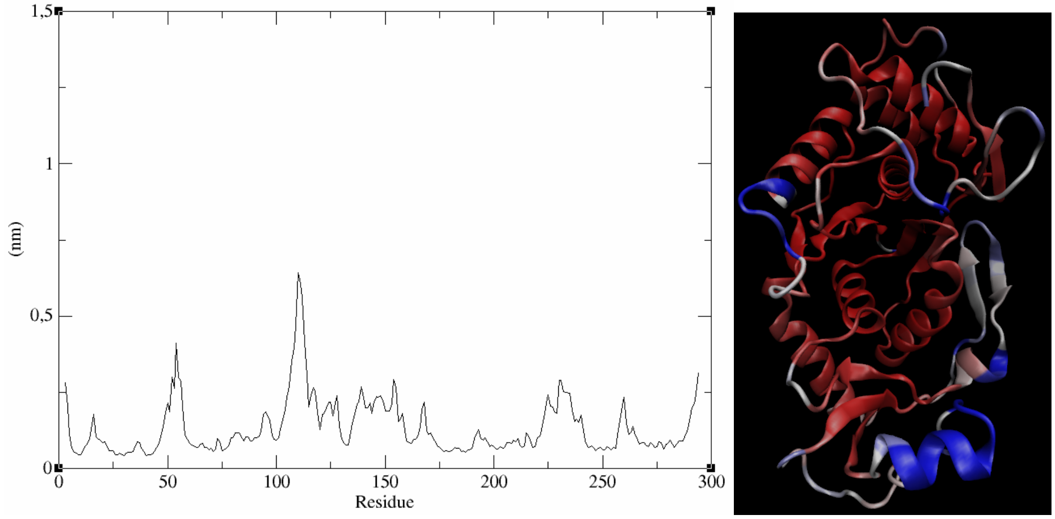

GluQRS/Glu peptide bond repeat

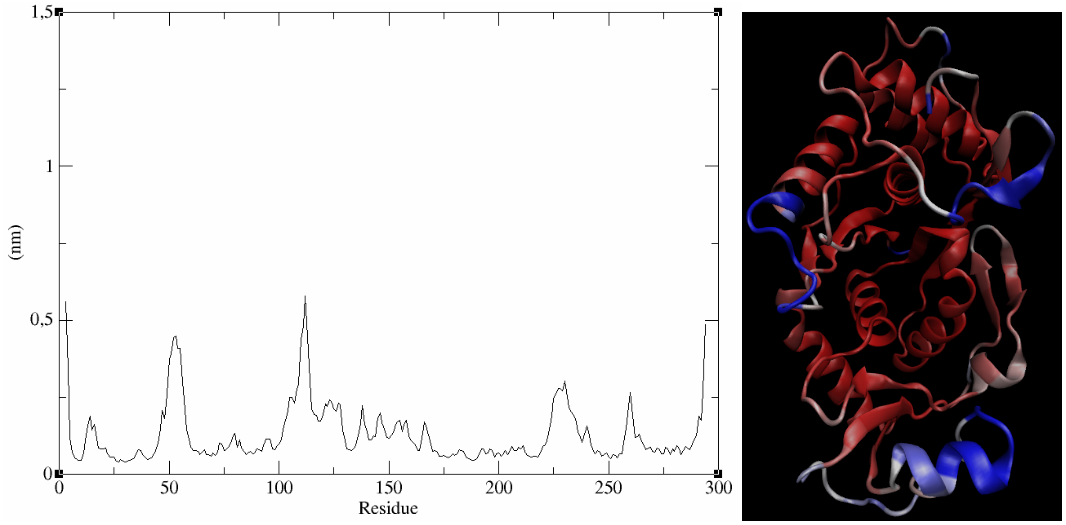

GluQRS/Glu modeled residues

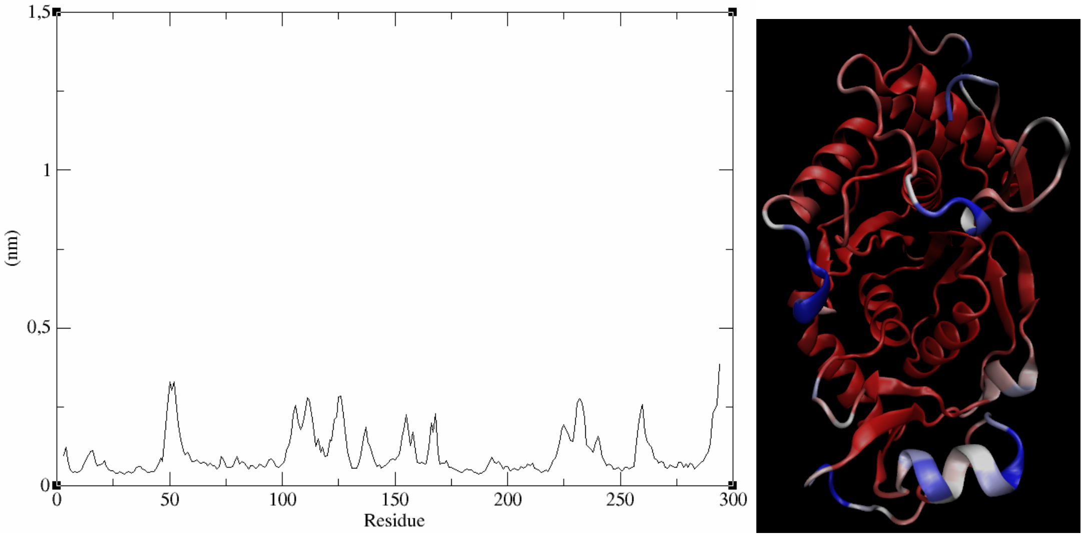

GluQRS/Glu modeled residues repeat

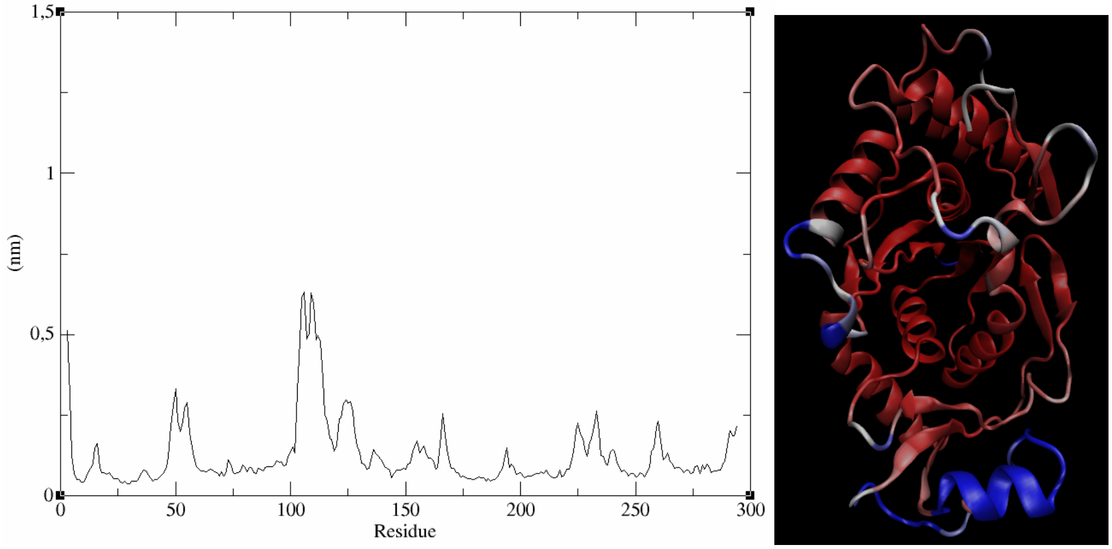

GluQRS/Glu peptide bond crystal site dock -6.5 kcal/mol

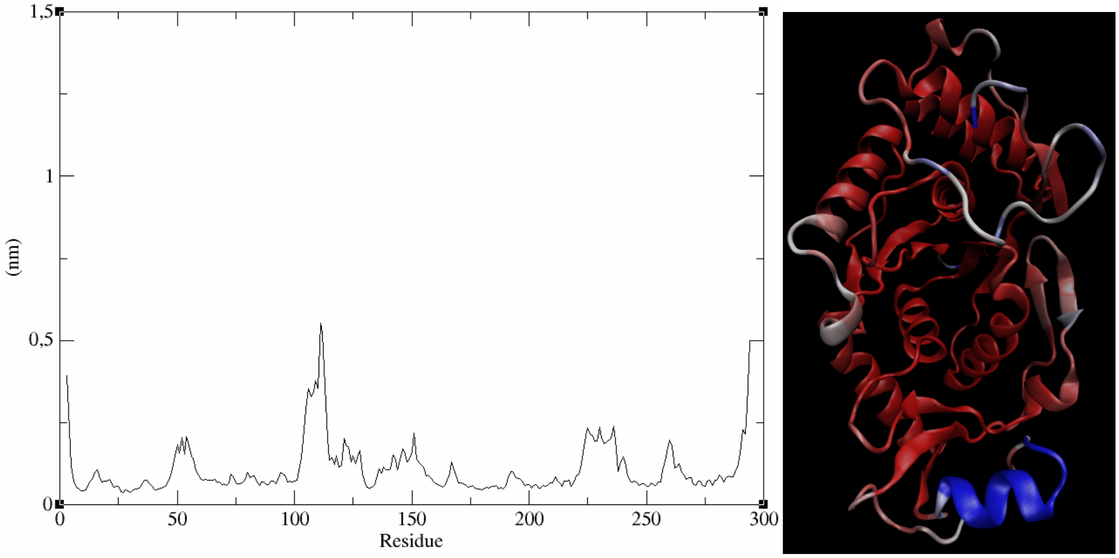

GluQRS/Glu peptide bond crystal site dock -6.5 kcal/mol repeat

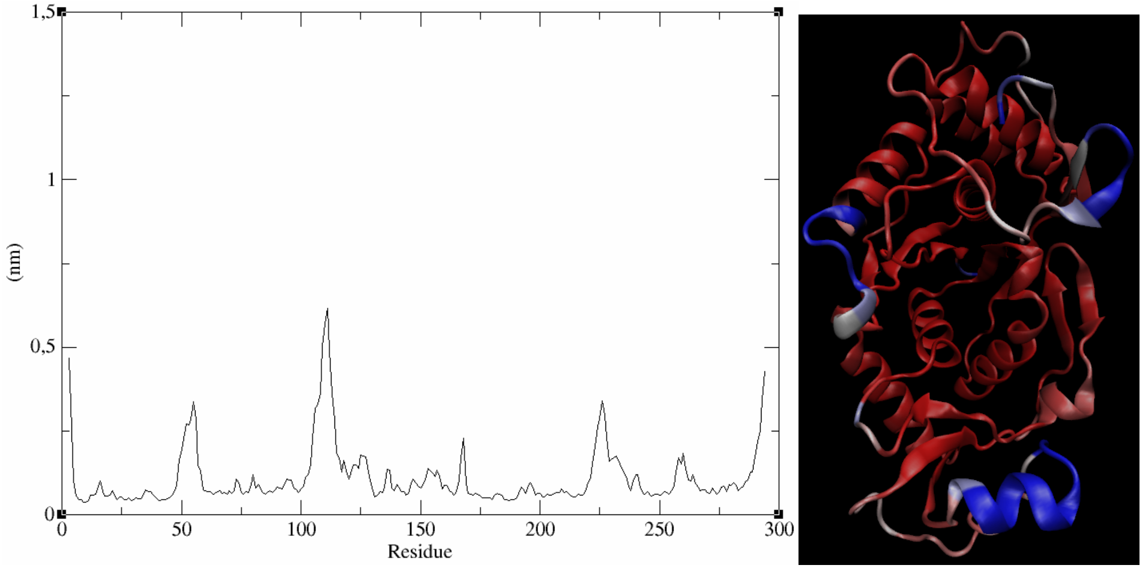

GluQRS/Glu peptide bond crystal site dock -4.5 kcal/mol

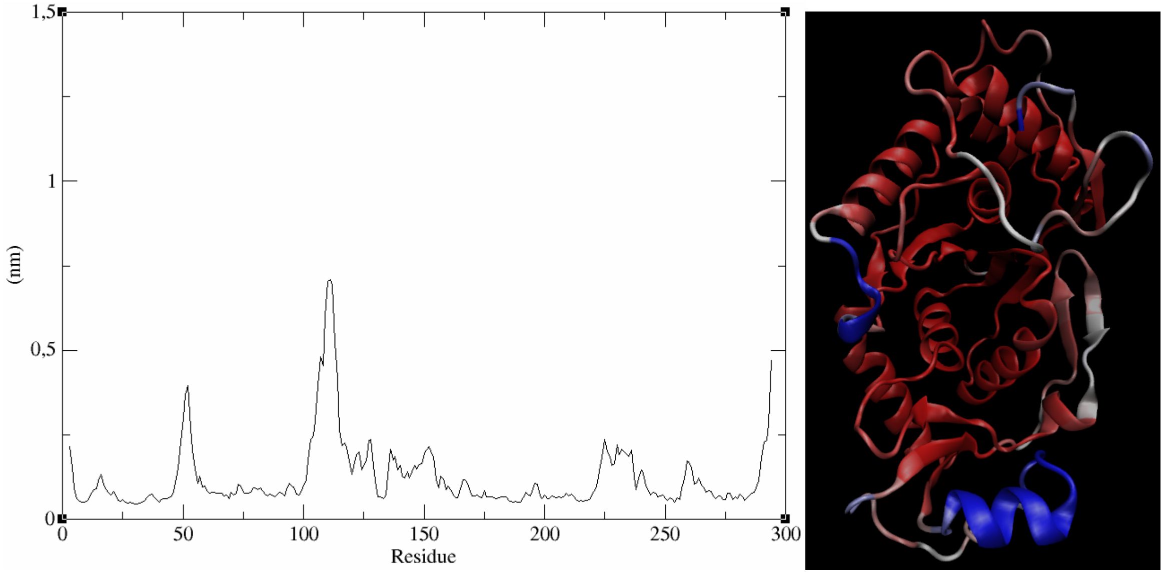

GluQRS/Glu novel site dock -6.0 kcal/mol

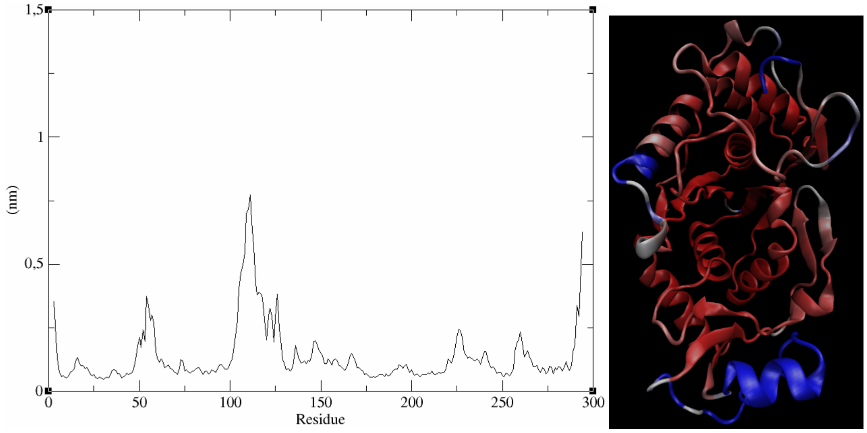

GluQRS/Asp

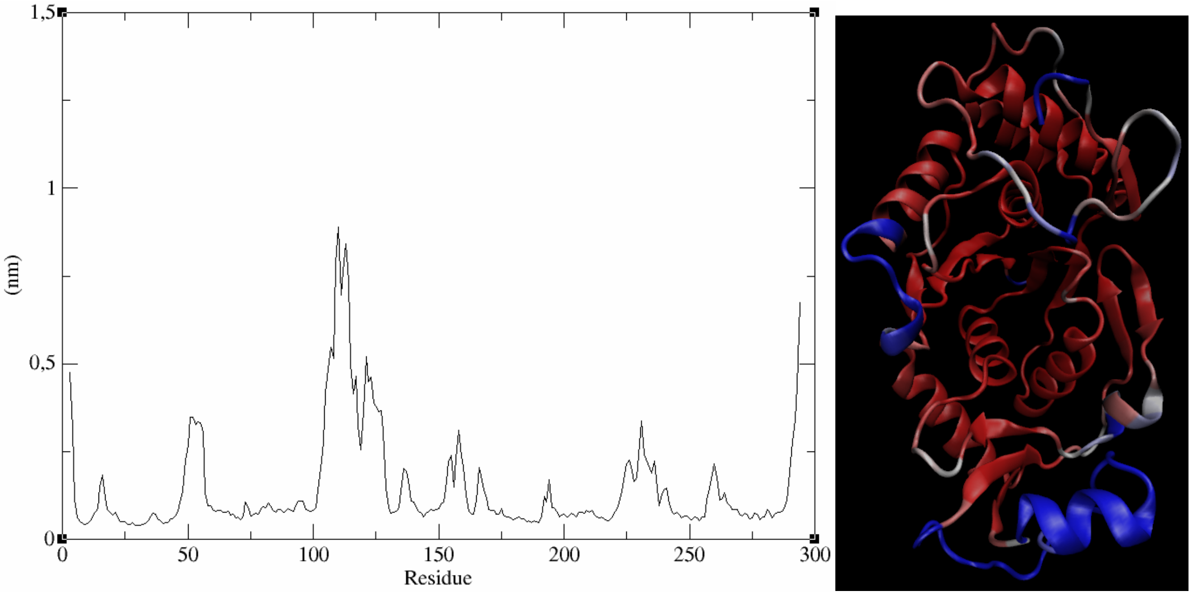

GluQRS/Asn

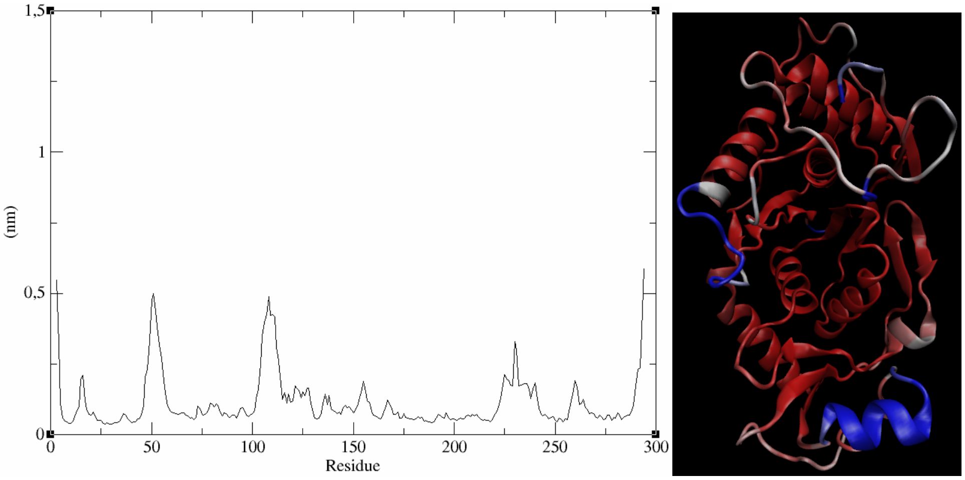

GluQRS/Ile

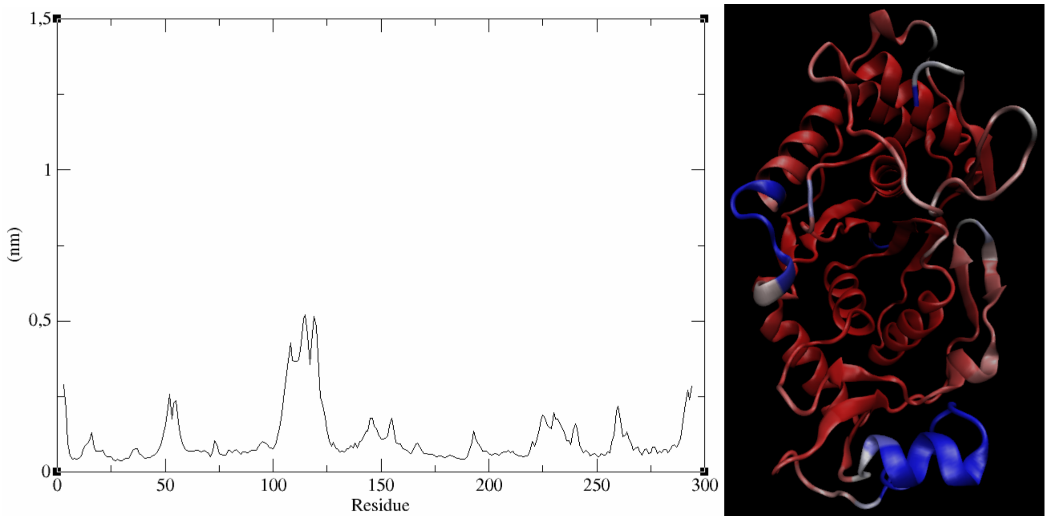

GluQRS/His

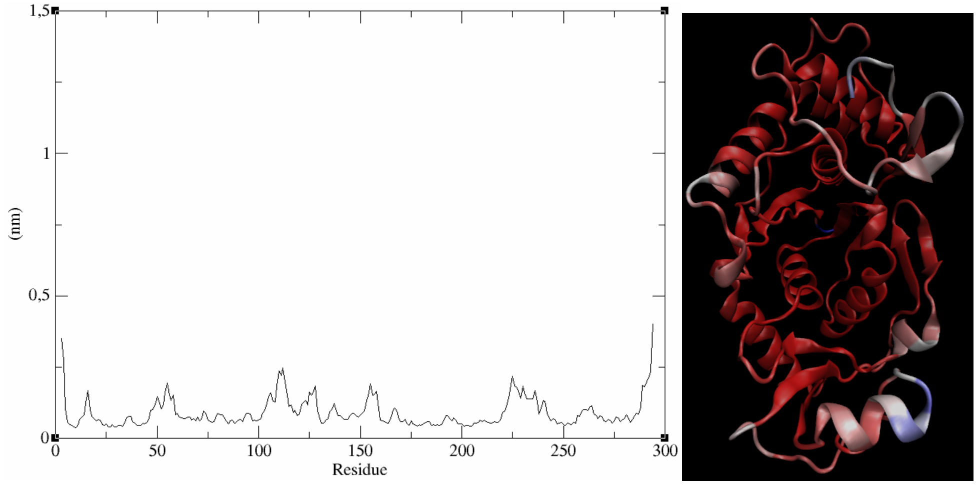

GluQRS/Met

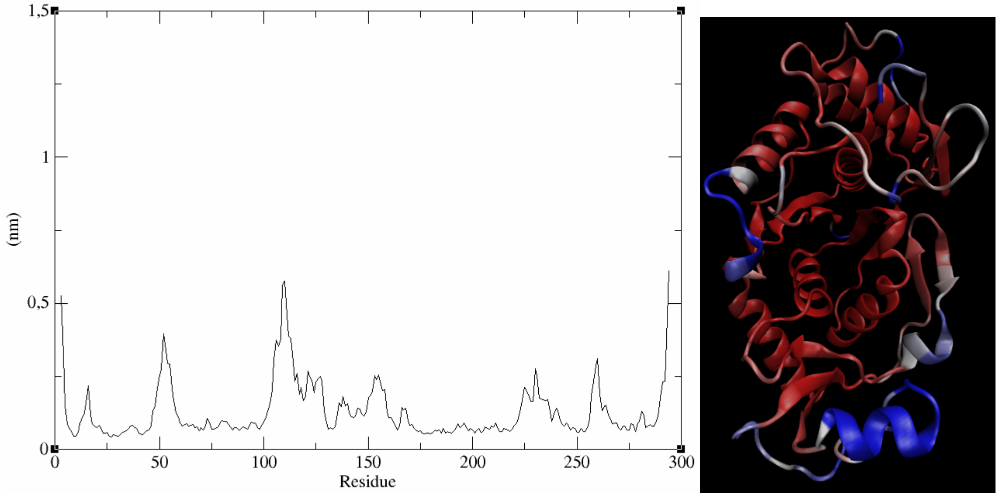

GluQRS/SAM

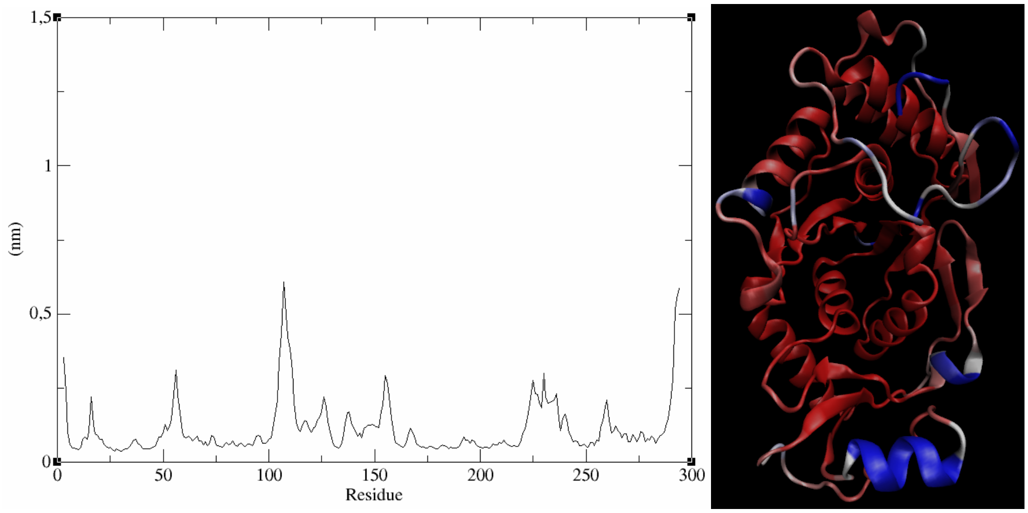

GluQRS/cyclic-AMP

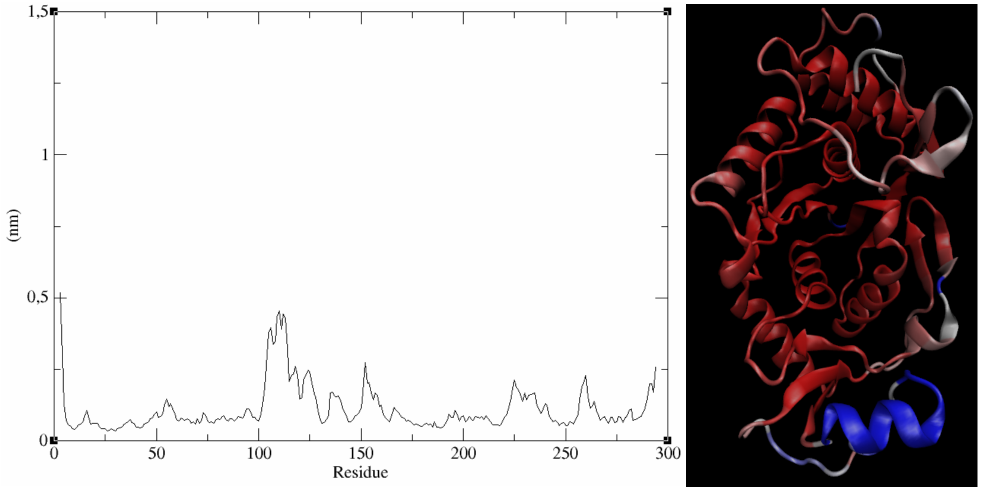

GluQRS/AMP @ ATP crystal site

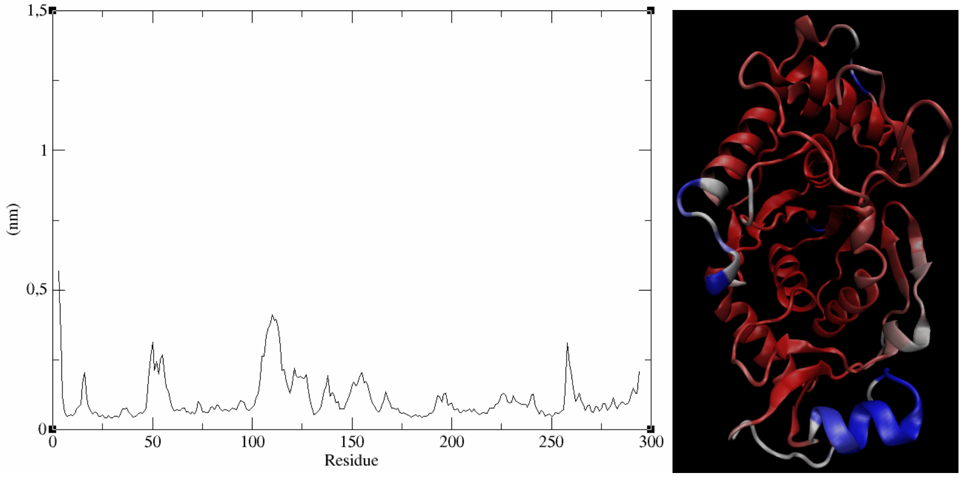

GluQRS/AMP @ Glu crystal site

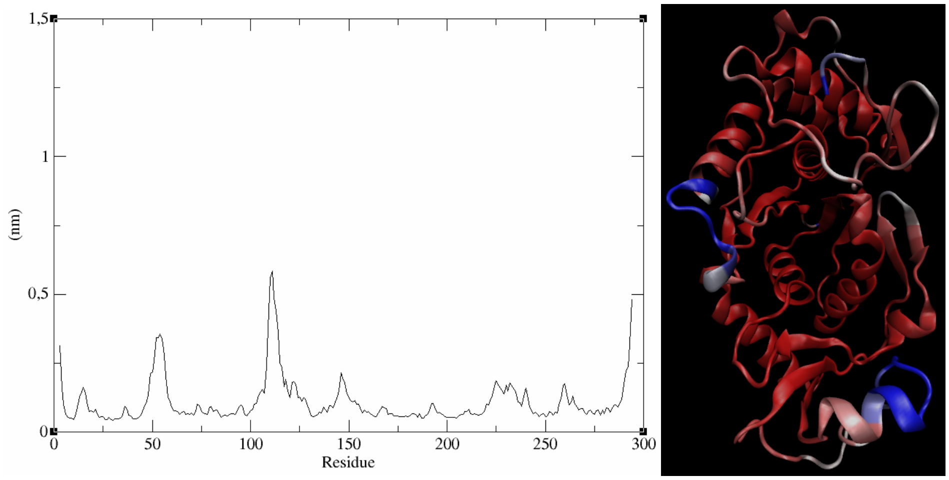

GluQRS/AMP novel site

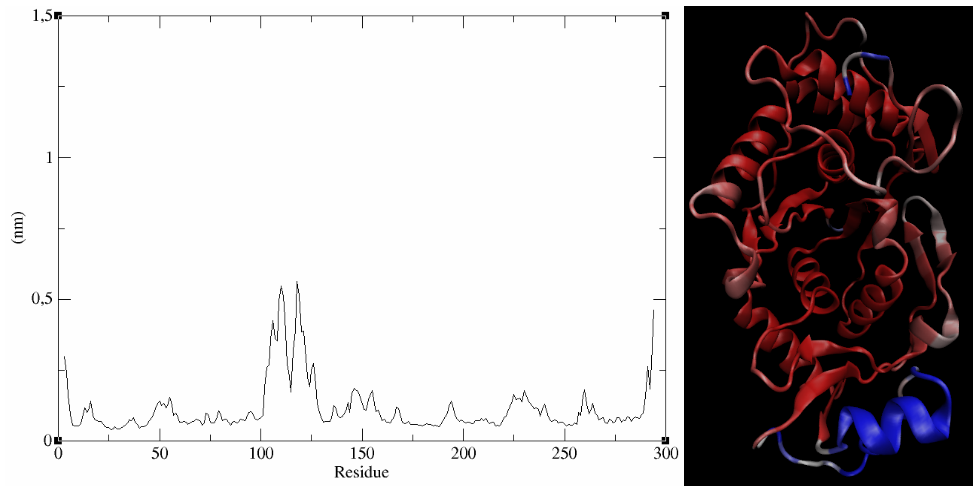
1,5
1
0,5
0
(nm)
0
50
100
150
200
250
300
Residue
