# Supplementary material for: An MD View of Ligand Binding
Source: Molecules. 2025 Dec 6;30(24):4678. doi: 10.3390/molecules30244678 (PMC12736043; doi:10.3390/molecules30244678)

Supplemental Figure S3 Histograms of atomic contact distances.

Contact distances populated at least 10% of the time during each one-microsecond simulation are defined on the x axis; population on the y axis. XL, interaction observed in the GluQRS/Glu crystal structure; DK, interaction observed in PoseEdit views of the docked pose selected for simulation.

GluQRS/Glu peptide bond

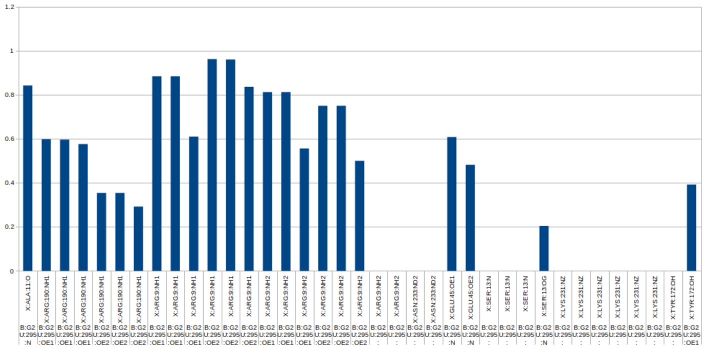

GluQRS/Glu peptide bond repeat

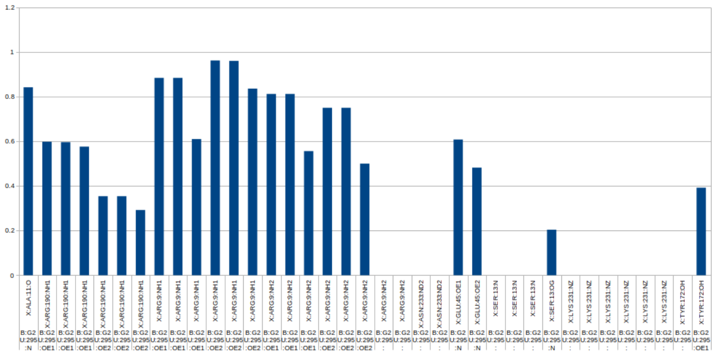

GluQRS/Glu modeled residues

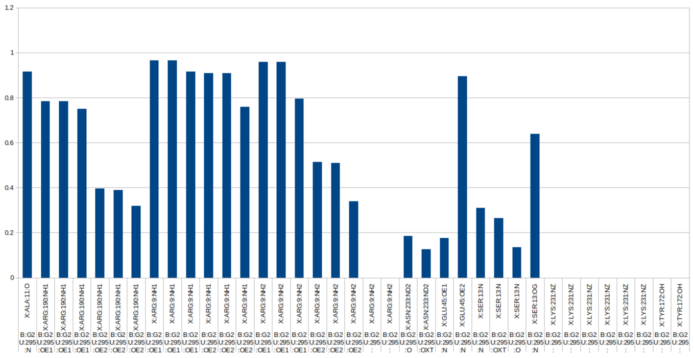

GluQRS/Glu modeled residues repeat

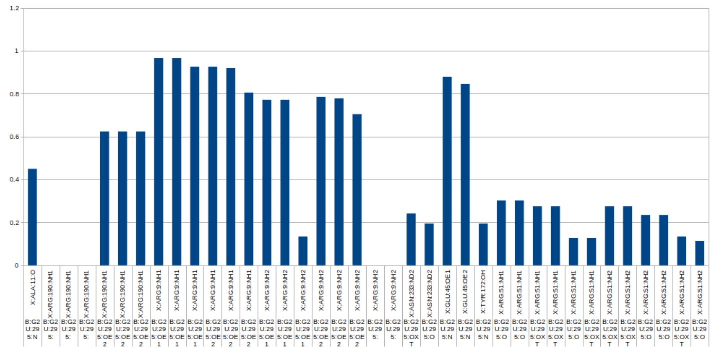

GluQRS/Glu docked @ crystal site -6.5 kcal/mol

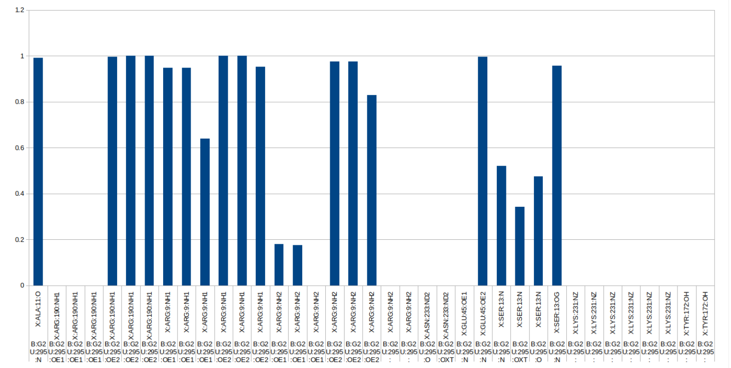

GluQRS/Glu docked @ crystal site -6.5 kcal/mol repeat

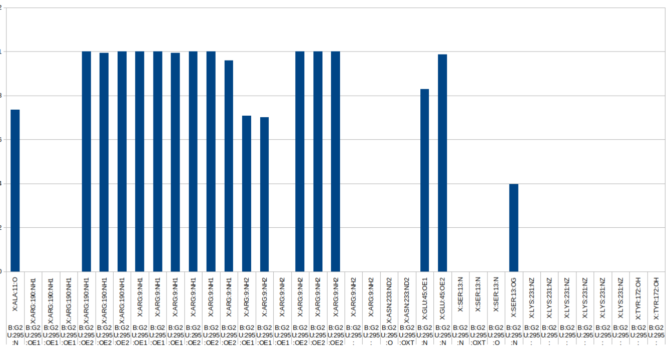

GluQRS/Glu docked @ crystal site -4.5 kcal/mol

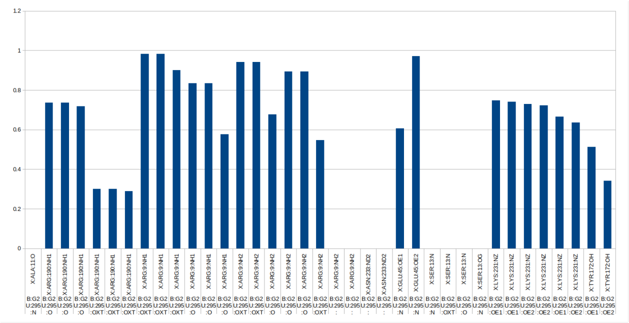

GluQRS/Glu docked @ novel site

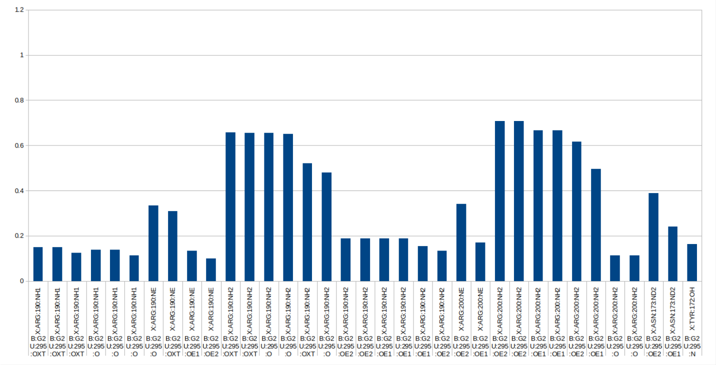

GluQRS/Asp

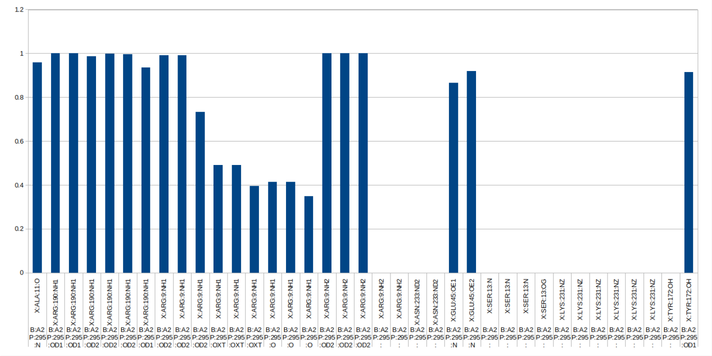

GluQRS/Asn

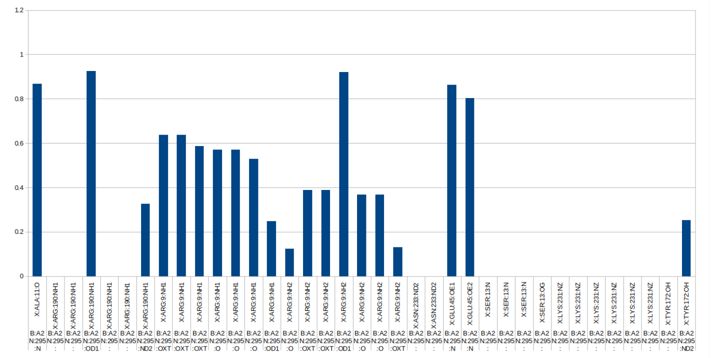

GluQRS/His

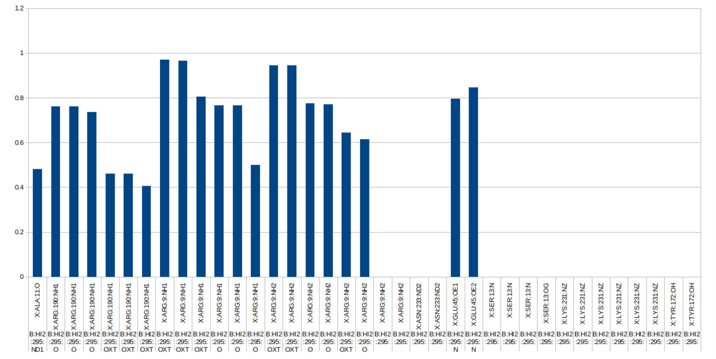

GluQRS/Ile

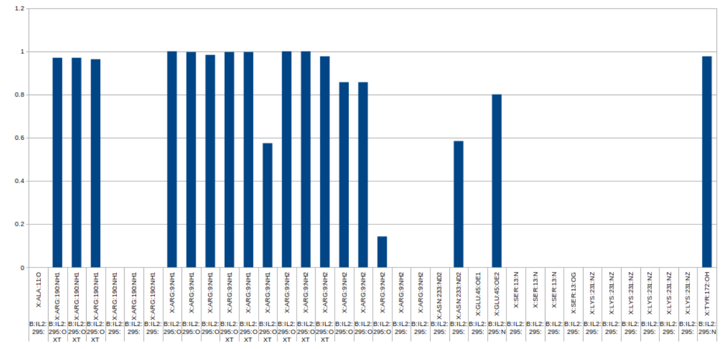

GluQRS/Met

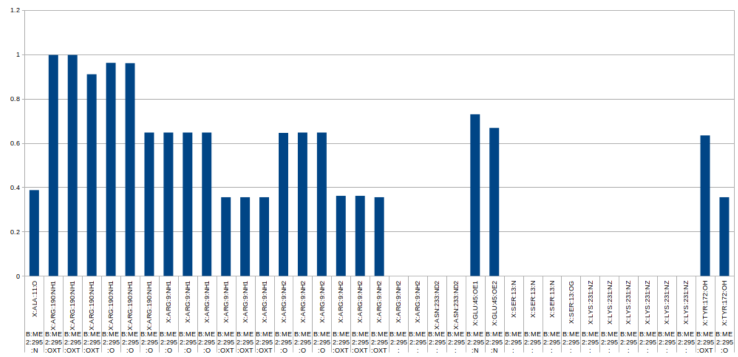

GluQRS/SAM

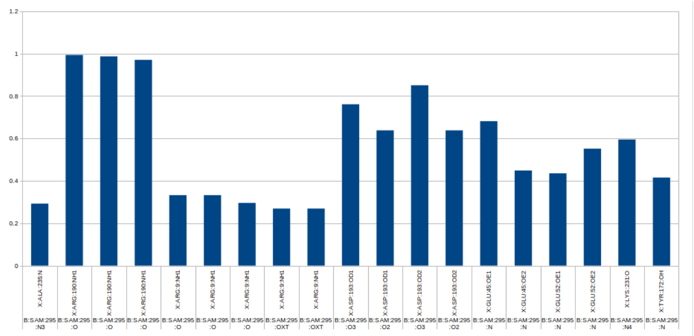

GluQRS/Cyclic-AMP

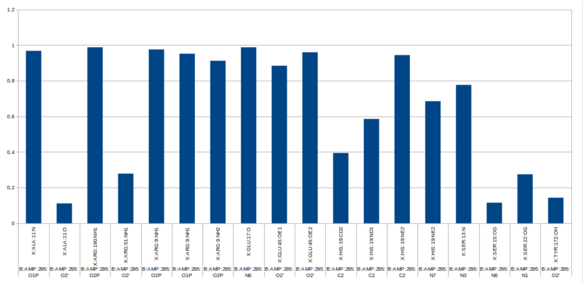

GluQRS/AMP @ AMP modeled site

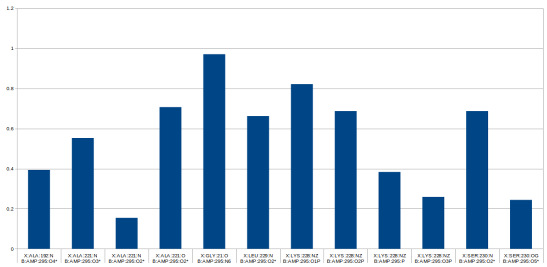

Supplement: Supplementary file 1 [file molecules-30-04678-s001.zip › Supplemental Figure S3 Histograms of atomic contact distances..pdf]
