# Supplementary material for: An MD View of Ligand Binding
Source: Molecules. 2025 Dec 6;30(24):4678. doi: 10.3390/molecules30244678 (PMC12736043; doi:10.3390/molecules30244678)

**Supplemental Figure S4**

PoseEdits views of all ten docked structures of Glu

~ -6.5 kcal/mol

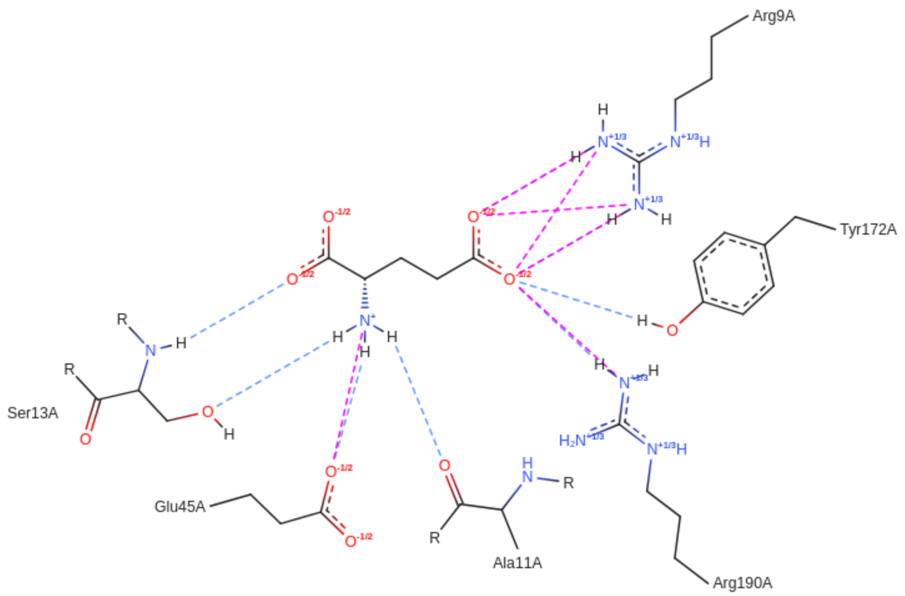


~ -6.4 kcal/mol

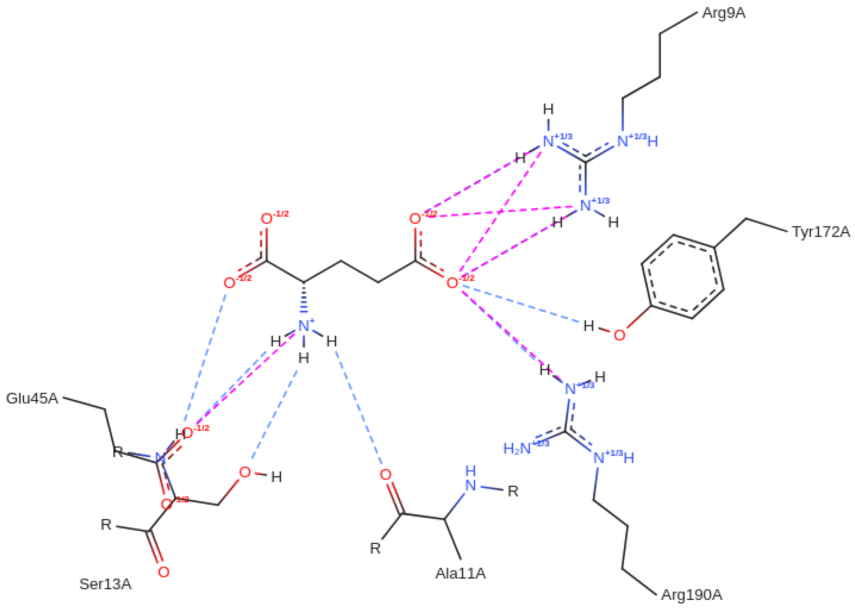

~ -6.2 kcal/mol

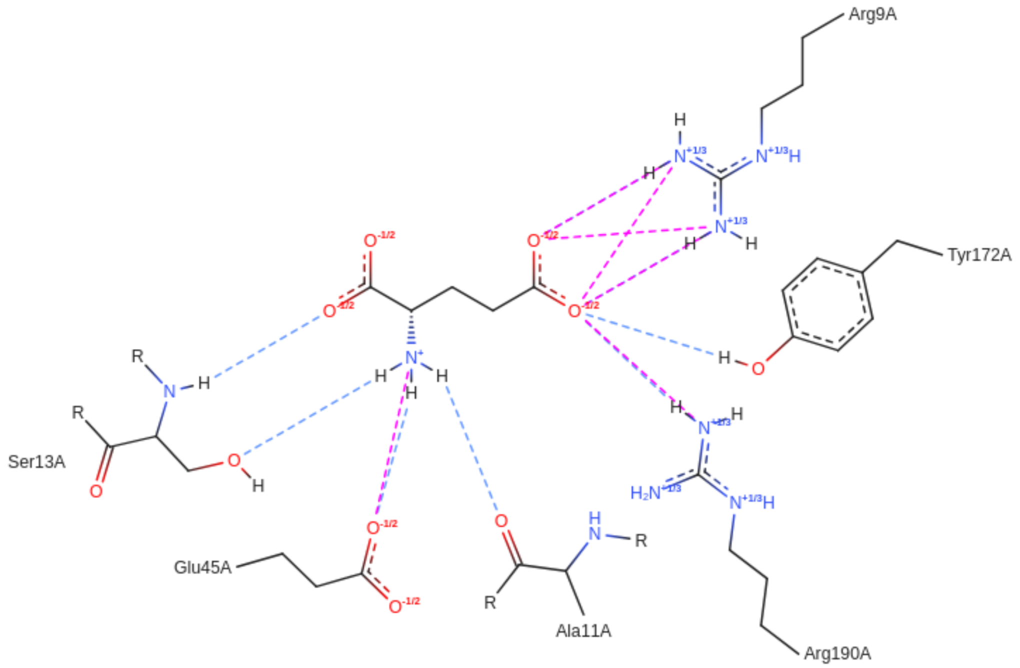


~ -4.5 kcal/mol

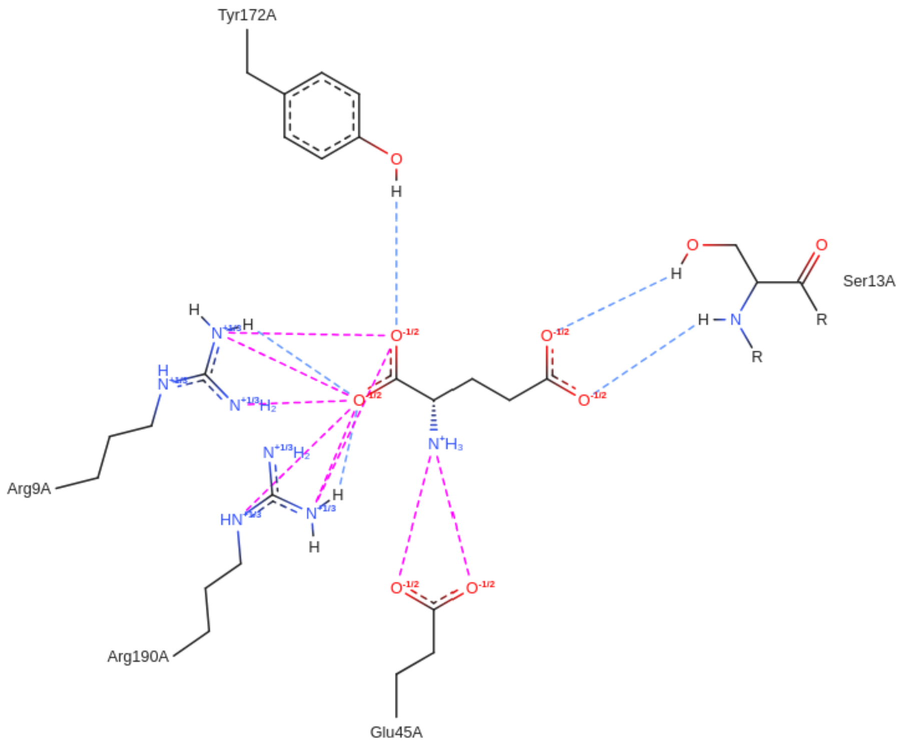

~ -4.5 kcal/mol

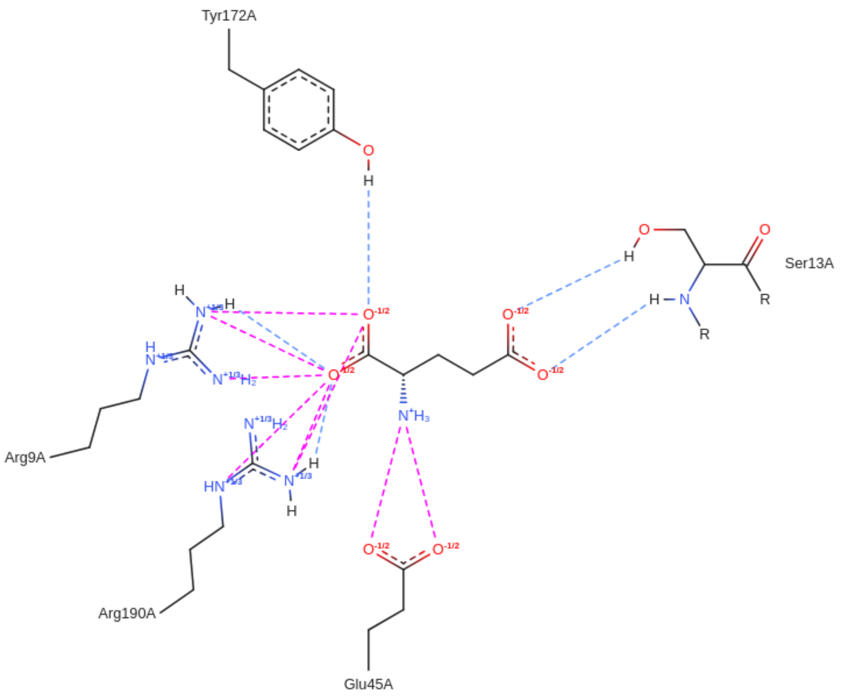


~ -4.5 kcal/mol

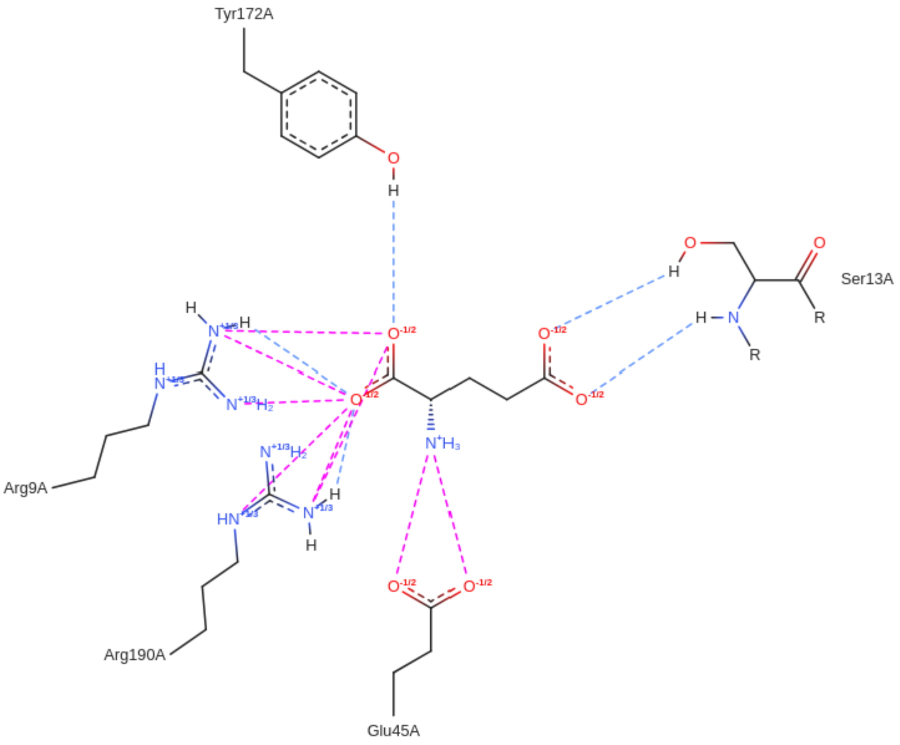

~ -4.5 kcal/mol

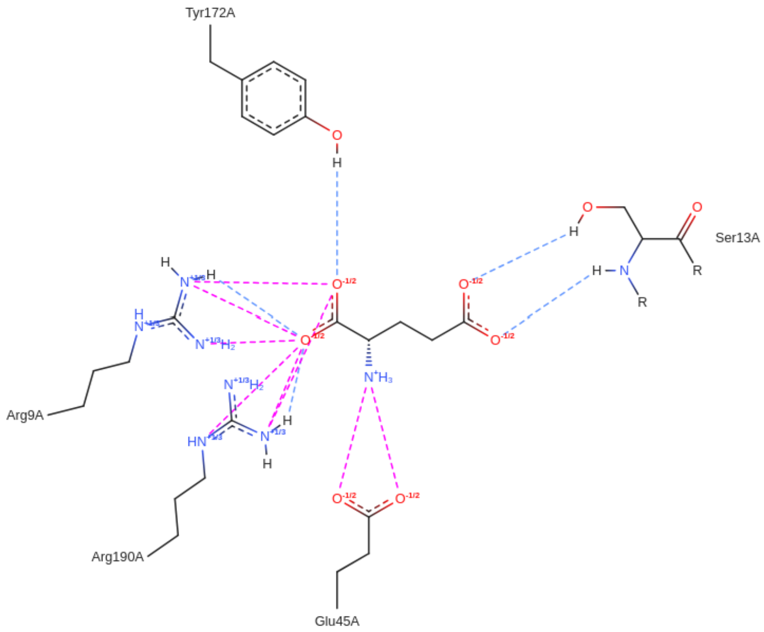


~ -4.5 kcal/mol

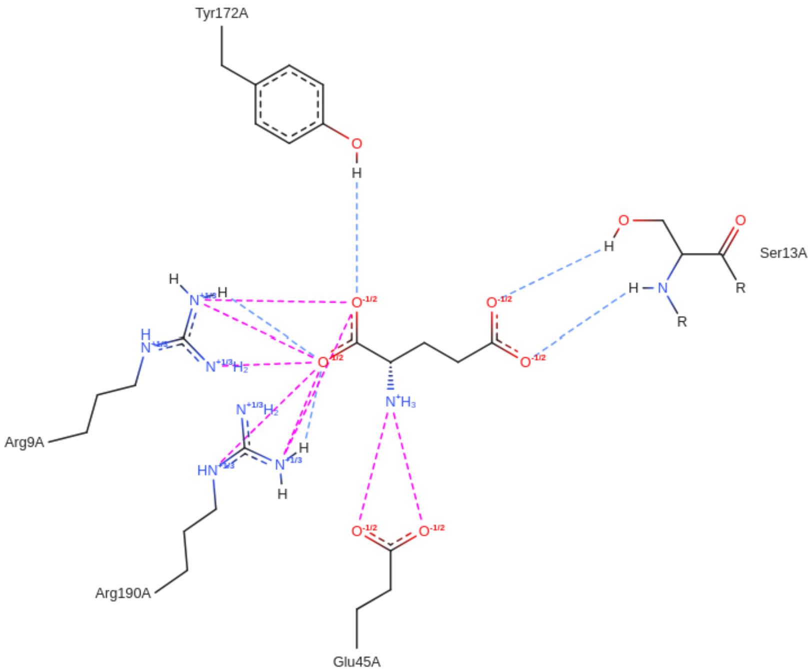

~ -4.5 kcal/mol

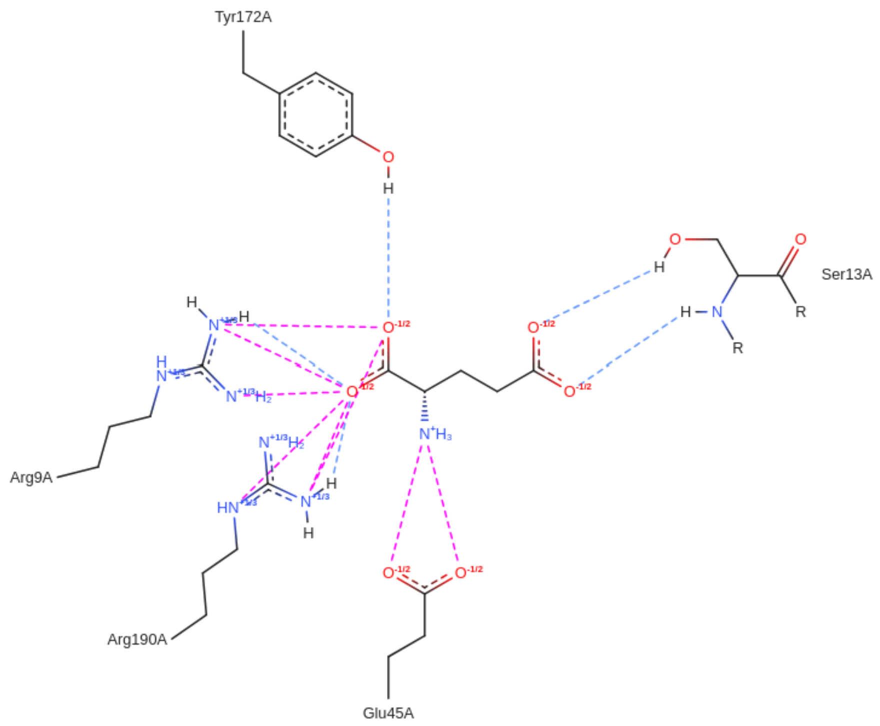


~ -4.25 kcal/mol

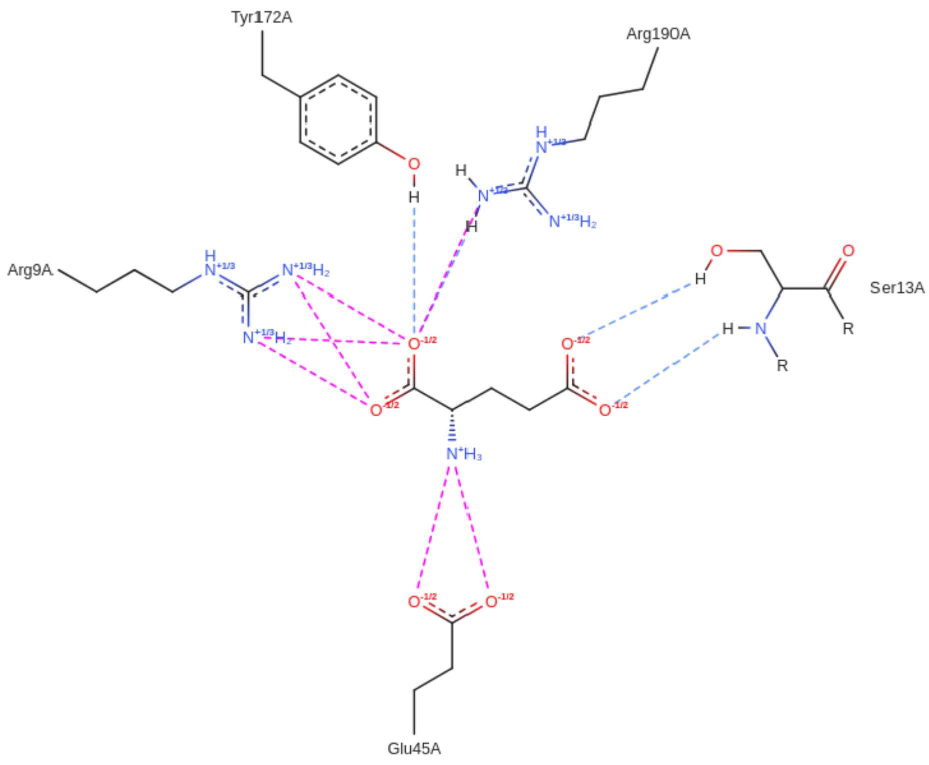

Supplement: Supplementary file 1 [file molecules-30-04678-s001.zip › Supplemental Figure S4 PoseEdit views of all ten docked structures of Glu.pdf]
