# Supplementary material for: An MD View of Ligand Binding
Source: Molecules. 2025 Dec 6;30(24):4678. doi: 10.3390/molecules30244678 (PMC12736043; doi:10.3390/molecules30244678)

Supplemental Figure S5 PoseEdit views of all analogs

as docked

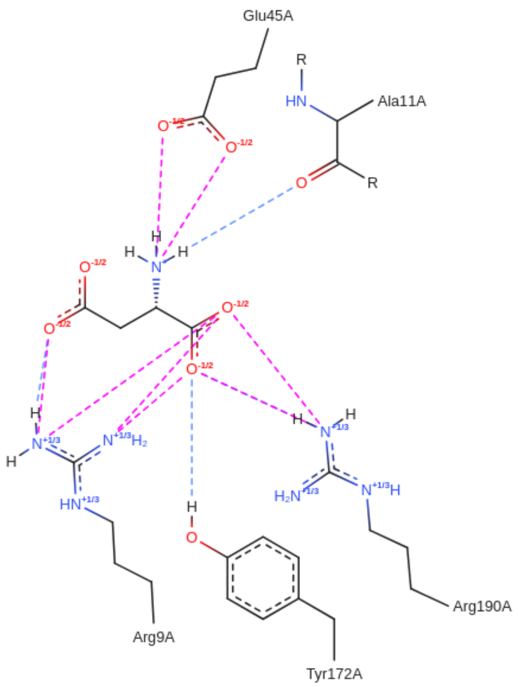

Asn

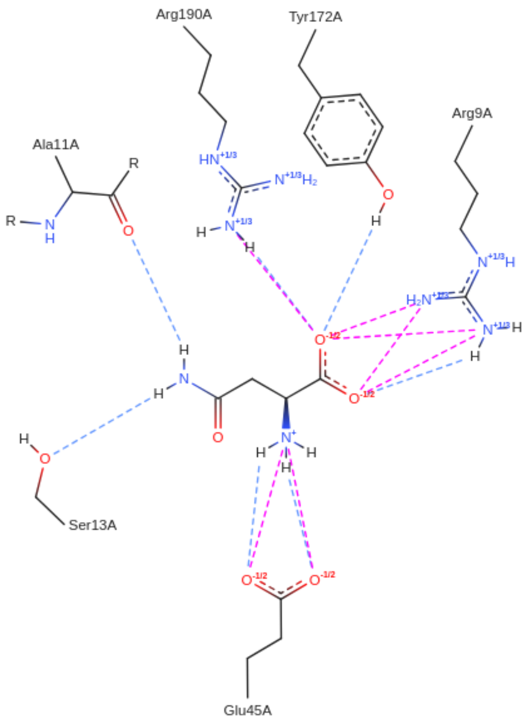

His

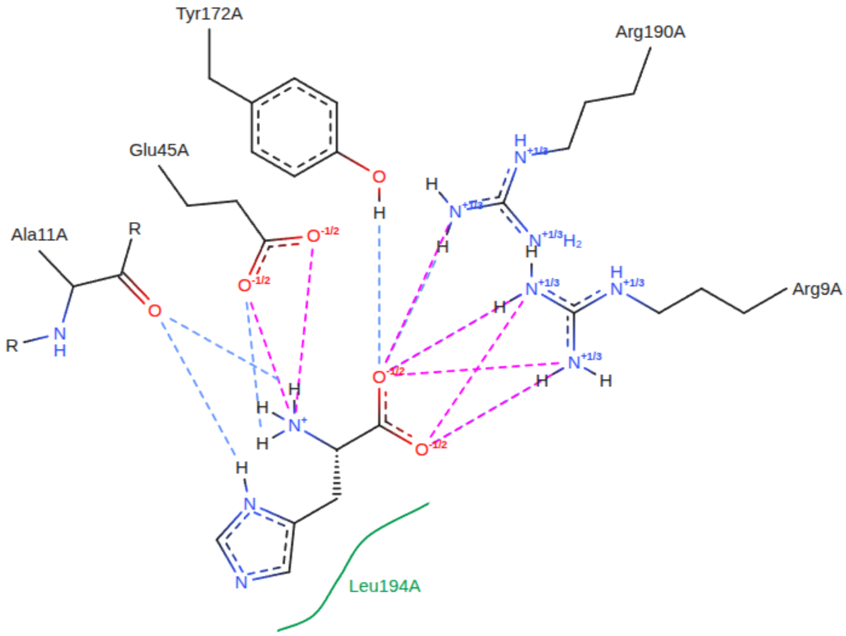

Tyr172A
Arg190A
Glu45A
Ala11A
Arg9A
Leu194A

Ile

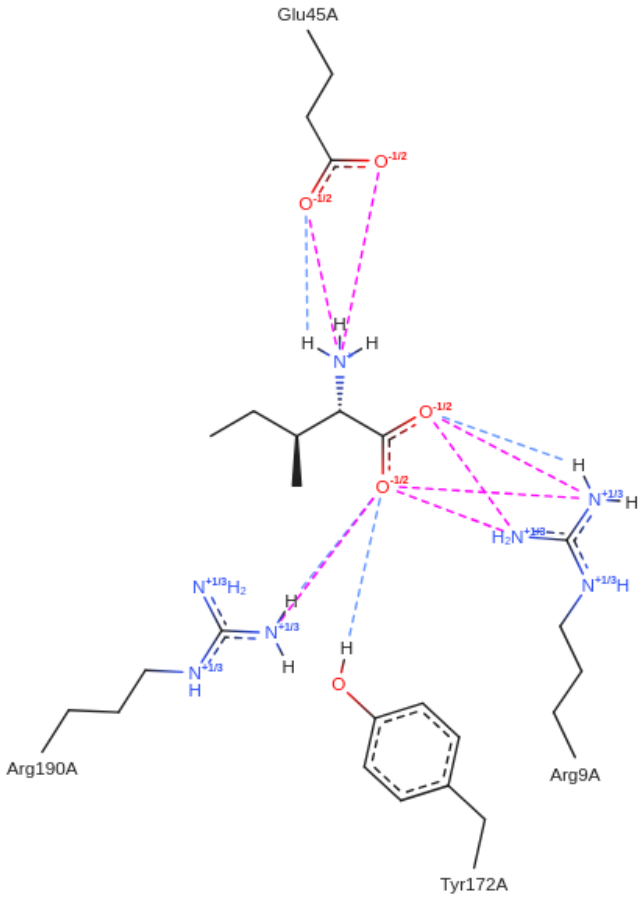

Glu45A
Arg190A
Arg9A
Tyr172A

Met

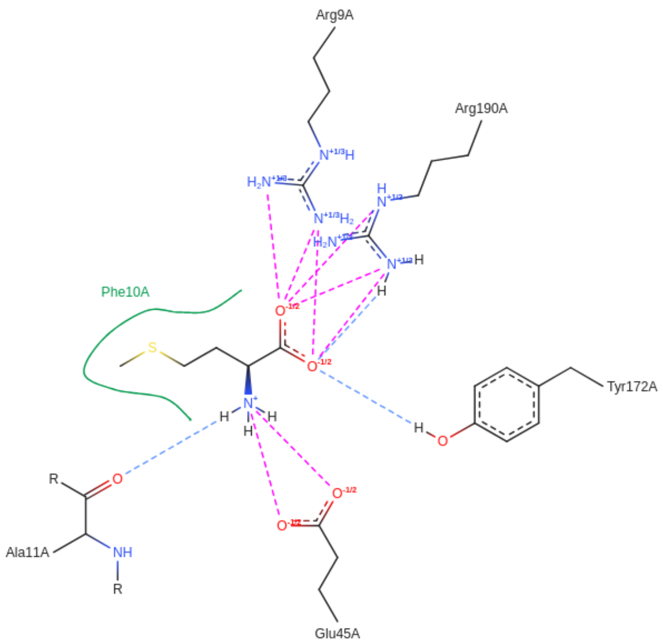
Arg9A
Arg190A
Phe10A
Tyr172A
Ala11A
Glu45A

SAM

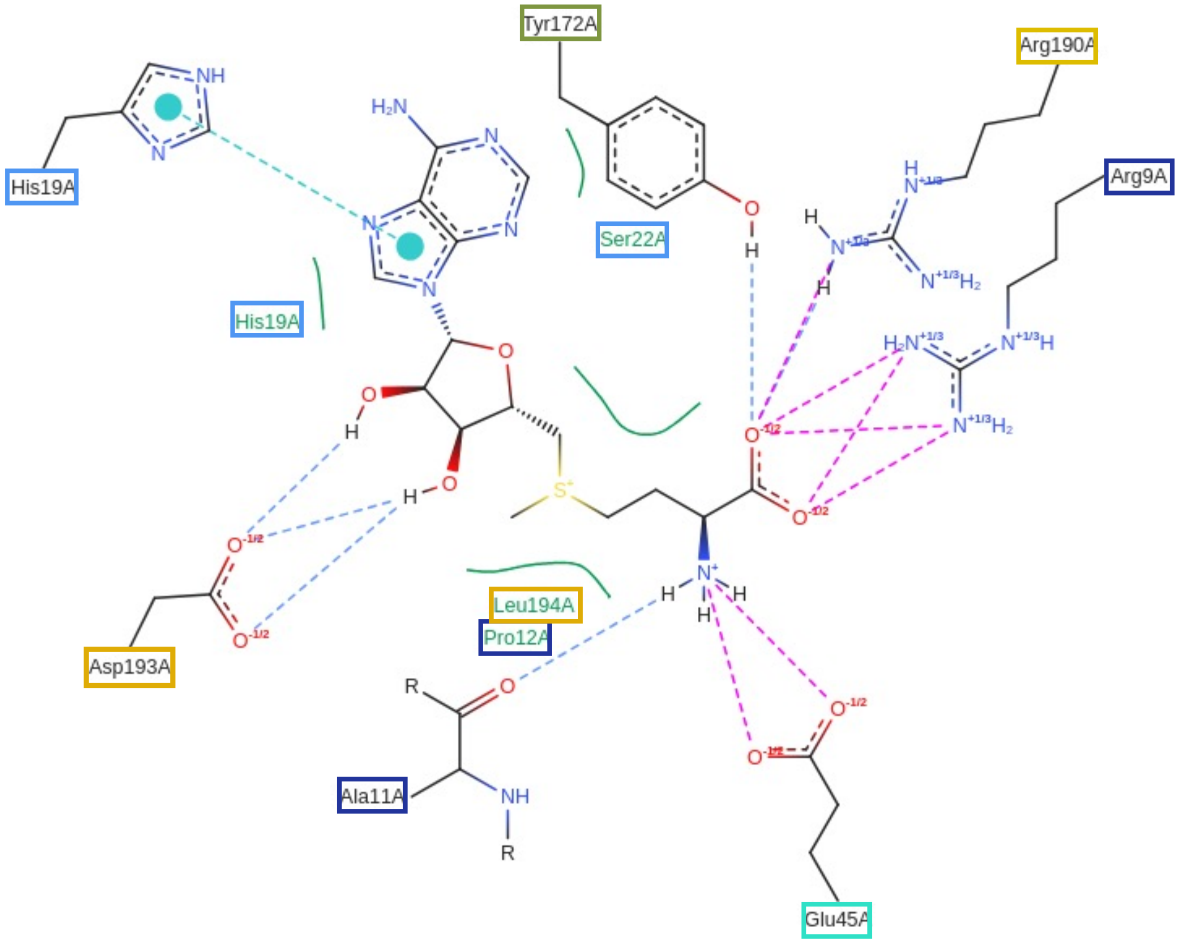
Tyr172A
Arg190A
His19A
Arg9A
Ser22A
His19A
Leu194A
Pro12A
Asp193A
Ala11A
Glu45A

cAMP

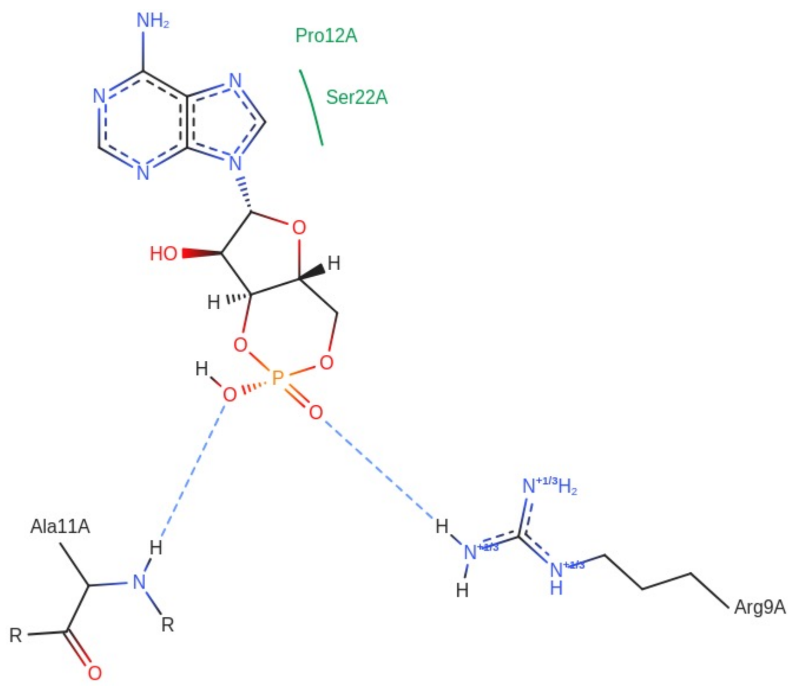

AMP @ AMP crystal site

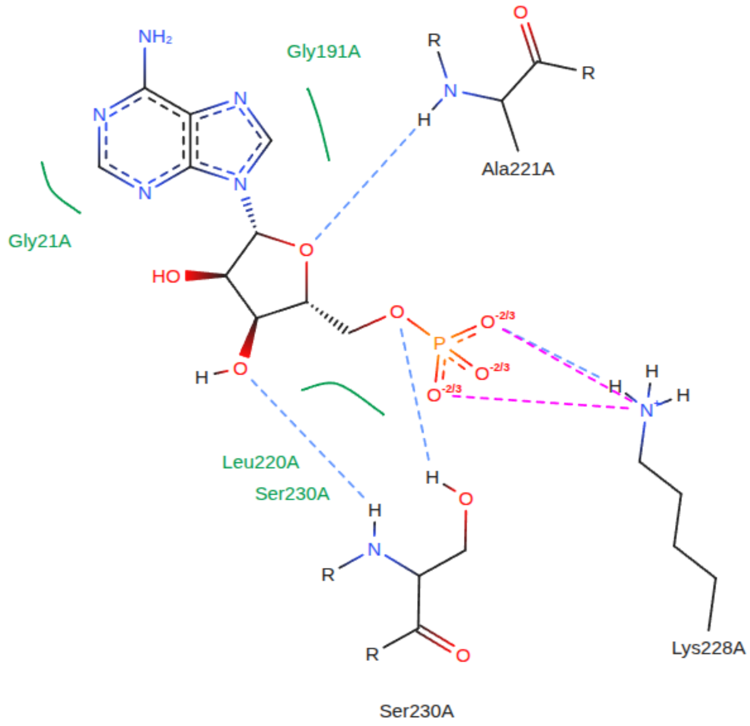

AMP @ Glu crystal site

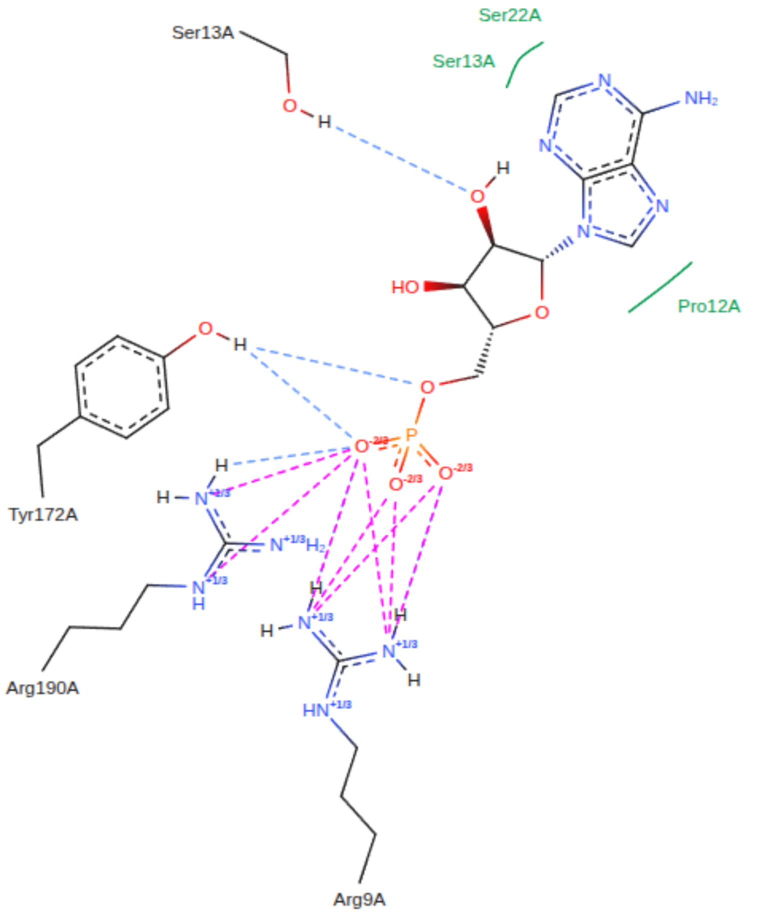

AMP @ novel site

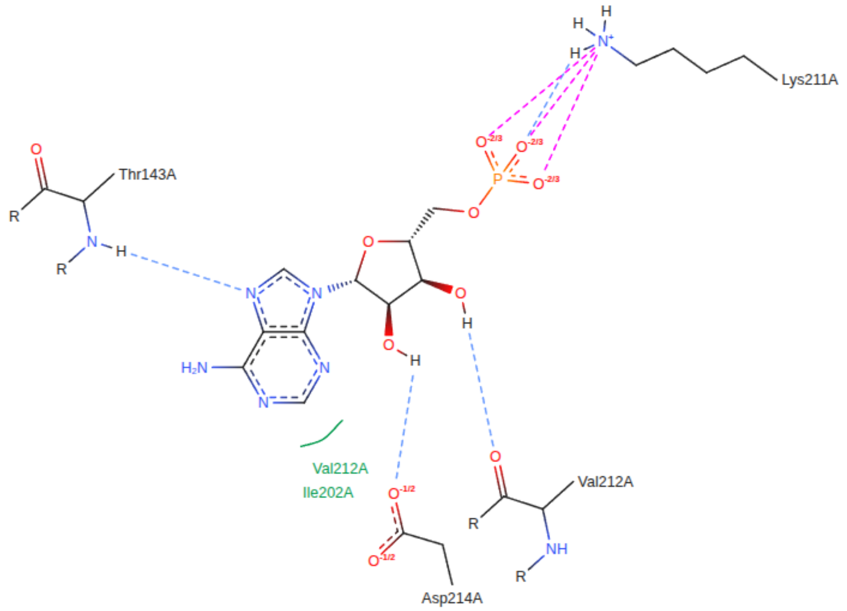

Supplement: Supplementary file 1 [file molecules-30-04678-s001.zip › Supplemental Figure S5 PoseEdits of all analogs as docked.pdf]
