# Supplementary material for: An MD View of Ligand Binding
Source: Molecules. 2025 Dec 6;30(24):4678. doi: 10.3390/molecules30244678 (PMC12736043; doi:10.3390/molecules30244678)

Supplemental Figure S6 conformational changes of Asp

PoseEdit views of conformational changes of Asp.

20 ns

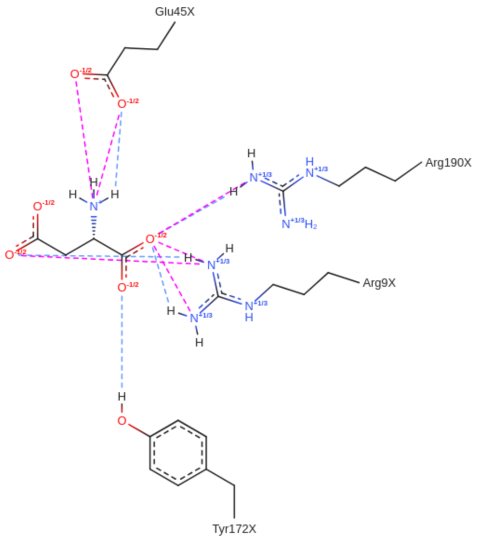

92 ns

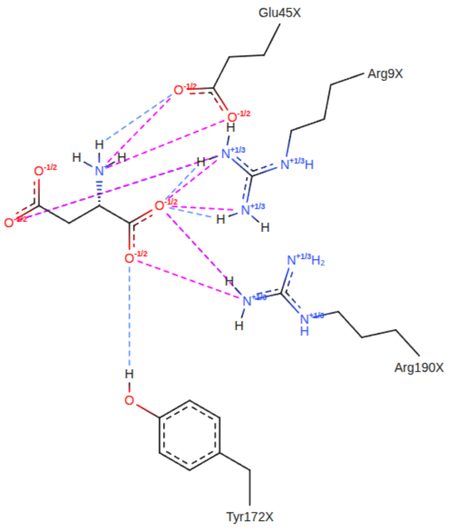

105 ns

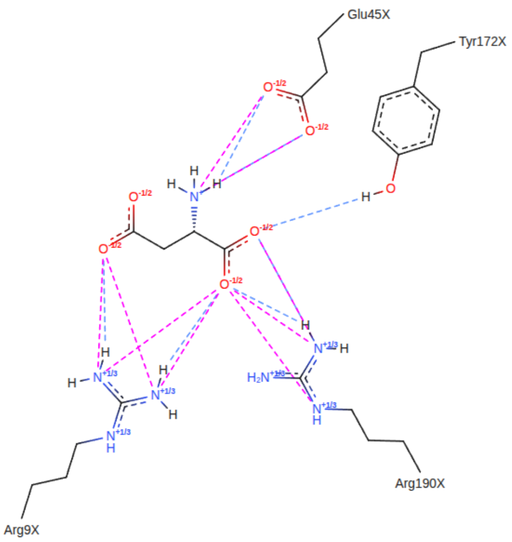

112 ns

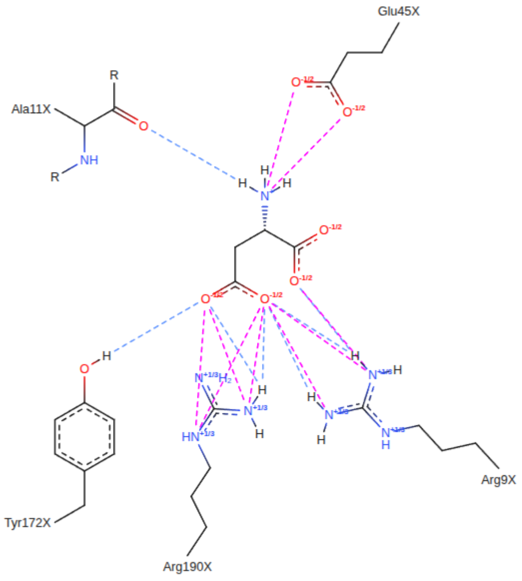

Supplement: Supplementary file 1 [file molecules-30-04678-s001.zip › Supplemental Figure S6 PoseEdit views of conformational changes of Asp.pdf]
