# Supplementary material for: An MD View of Ligand Binding
Source: Molecules. 2025 Dec 6;30(24):4678. doi: 10.3390/molecules30244678 (PMC12736043; doi:10.3390/molecules30244678)

Supplemental Figure S8. Histograms of GBSA system energies. GBSA dot plots vs. time for each simulation were grouped (y axis) according to energy in bins of width 1 kcal/mol (x axis). Scale expansions (inset) are included where needed for full-height bins.

A. GluQRS/Glu crystal structure, 232-235 peptide bond, #1 (left), #2 (right)

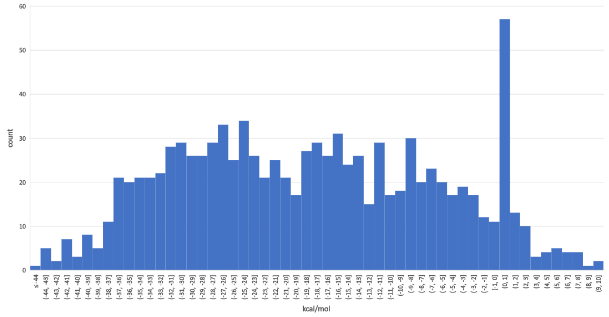

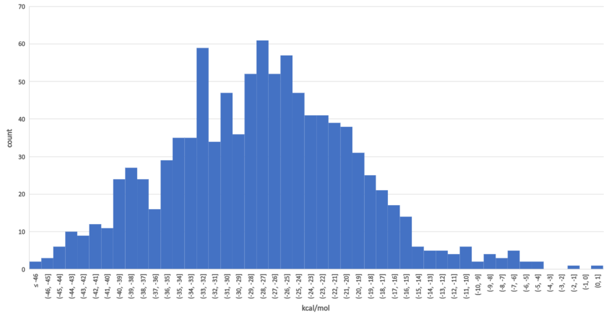

B. GluQRS/Glu crystal structure, 233-234 modeled residues, #1 (left), #2 (right)

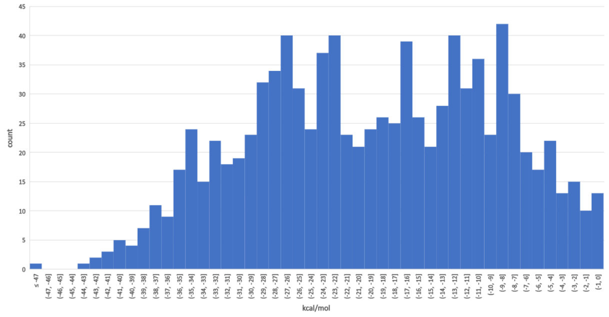

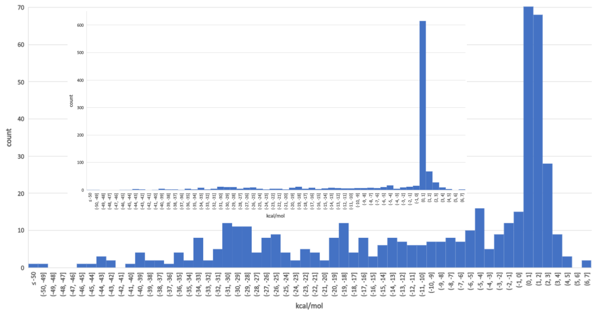

C. GluQRS/Glu dock ~ -6.5 kcal/mol, 232-235 peptide bond, #1 (left), #2 (right)

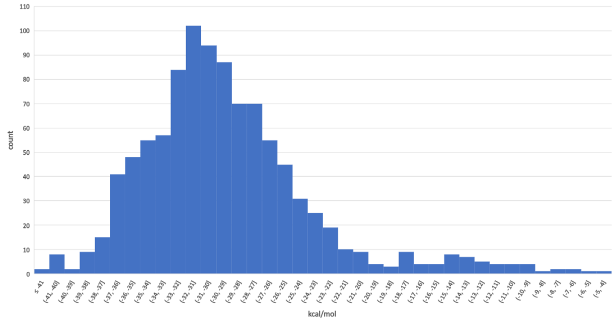

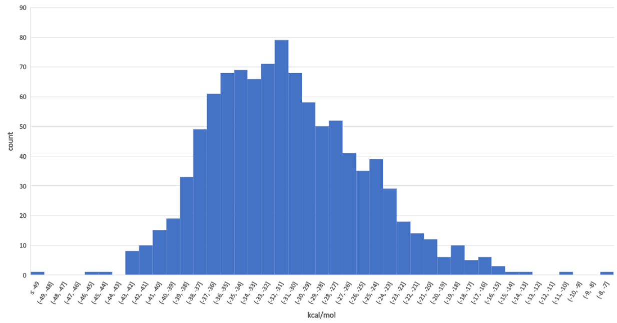

D. GluQRS/Glu dock ~ -4.25 kcal/mol, 232-235 peptide bond (left)

E. GluQRS/Glu dock ~ -5.0 kcal/mol, 232-235 peptide bond (right)

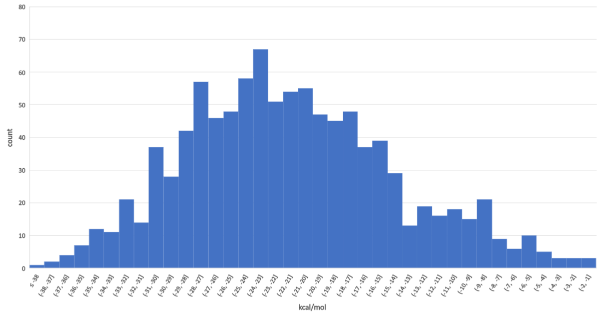

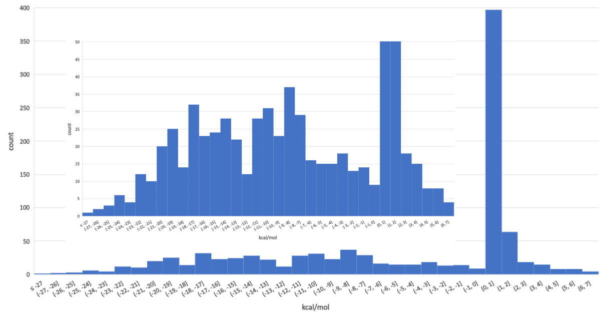

F. GluQRS/Asp dock ~ -4.65 kcal/mol, 232-235 peptide bond (left)

G. GluQRS/Asn dock ~ -6.45 kcal/mol, 232-235 peptide bond (right)

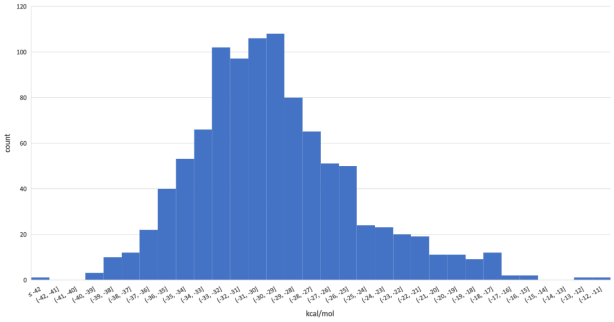

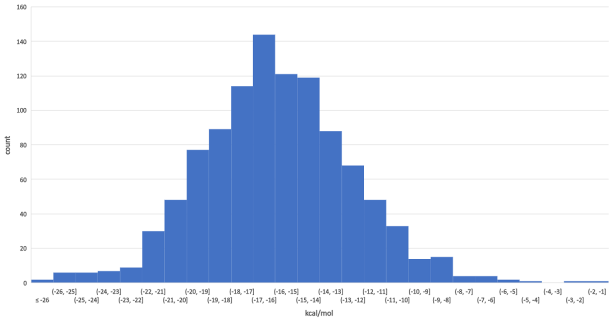

H. GluQRS/His dock ~ -6.25 kcal/mol, 232-235 peptide bond (left)

I. GluQRS/Ile dock ~ -5.8 kcal/mol, 232-235 peptide bond (right)

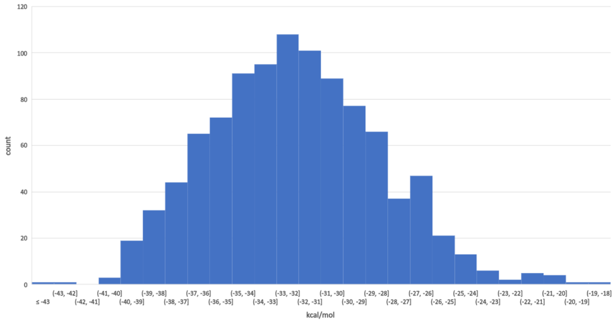

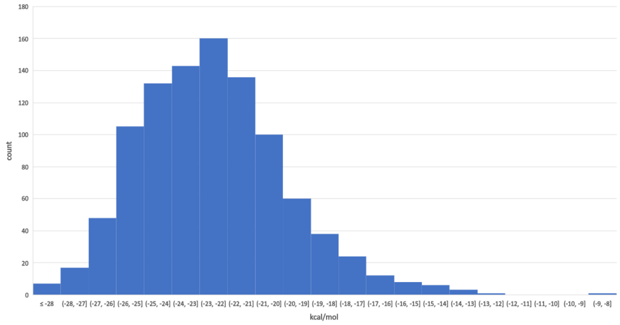

J. GluQRS/Met dock ~ -4.5 kcal/mol, 232-235 peptide bond (left)

K. GluQRS/SAM dock ~ -6.75 kcal/mol, 232-235 peptide bond (right)

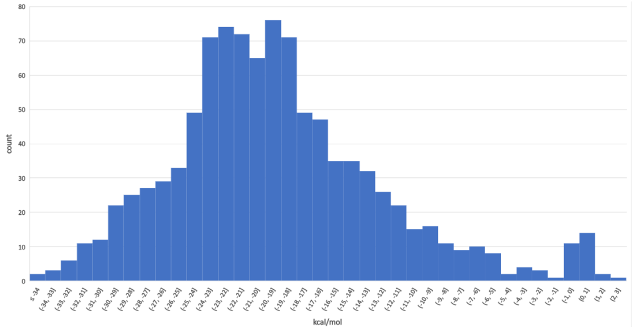

L. GluQRS/AMP @ AMP site dock ~ -5.0 kcal/mol, 232-235 peptide bond (left)

M. GluQRS/AMP @ Glu site dock ~ -5.0 kcal/mol, 232-235 peptide bond (right)

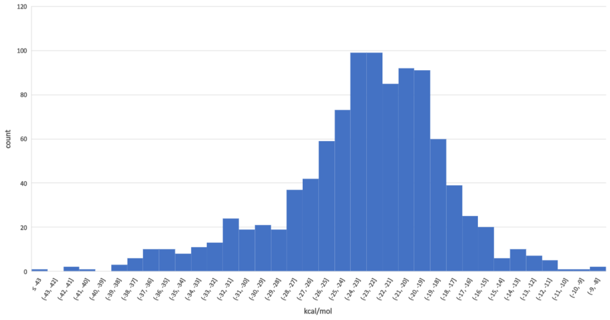

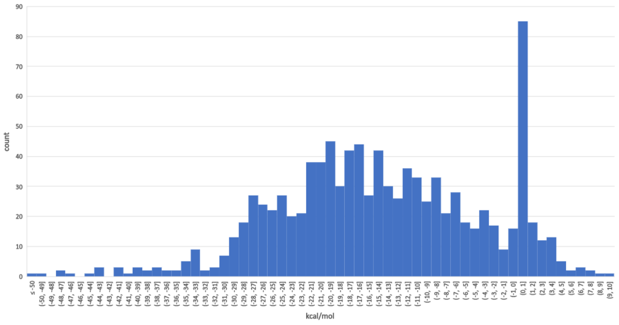

N. GluQRS/AMP @novel site dock ~ -6.25 kcal/mol, 232-235 peptide bond (left)

O. GluQRS/cAMP dock ~ -6.25 kcal/mol, 232-235 peptide bond (right)

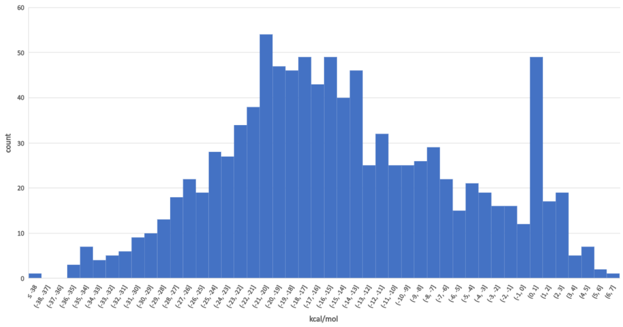

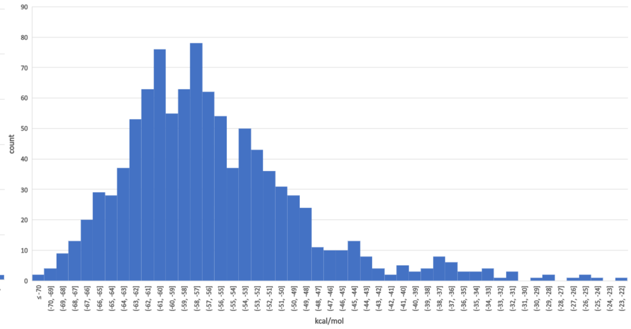

Supplement: Supplementary file 1 [file molecules-30-04678-s001.zip › Supplemental Figure S8 Histograms of GBSA system energies .pdf]
