# Supplementary material for: An MD View of Ligand Binding
Source: Molecules. 2025 Dec 6;30(24):4678. doi: 10.3390/molecules30244678 (PMC12736043; doi:10.3390/molecules30244678)
Supplement: Supplementary file 1 [file molecules-30-04678-s001.zip › Supplemental Figure S9 Gallery of results for AMP RMSD plots, GBSA energies, and PoseEdit views.pdf]

Supplemental Figure S9 Gallery of results for AMP: RMSD plots, GBSA energies, and PoseEdit views.

PEs **A. AMP at AMP crystal site**

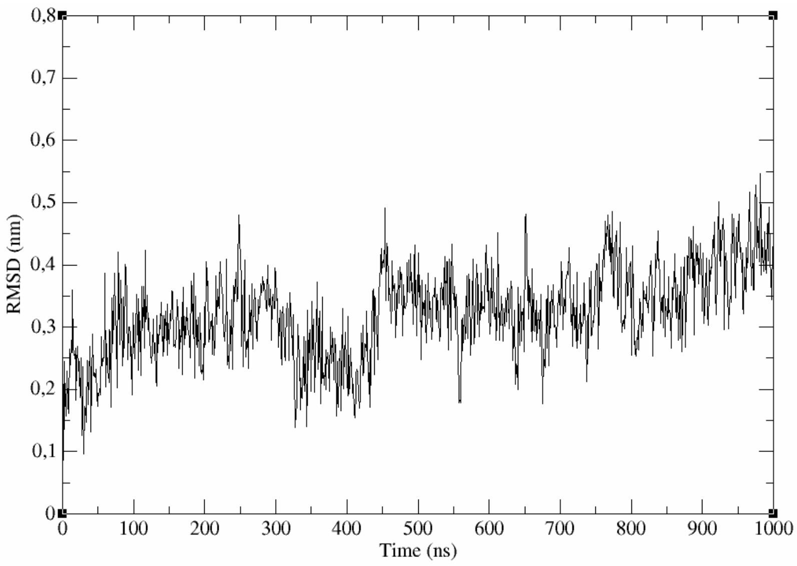

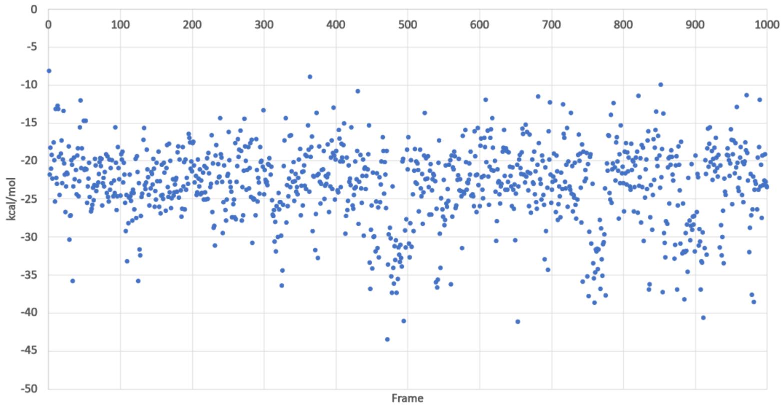

Docked

200 ns

400 ns

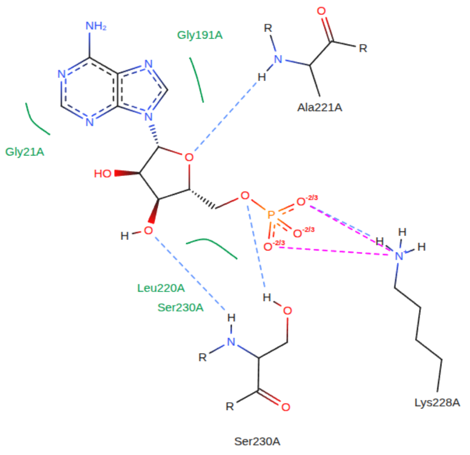

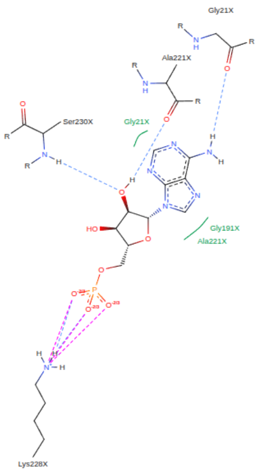

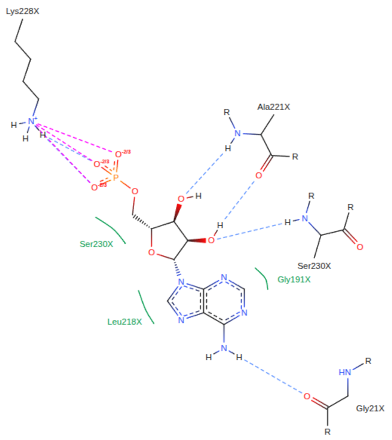

600 ns

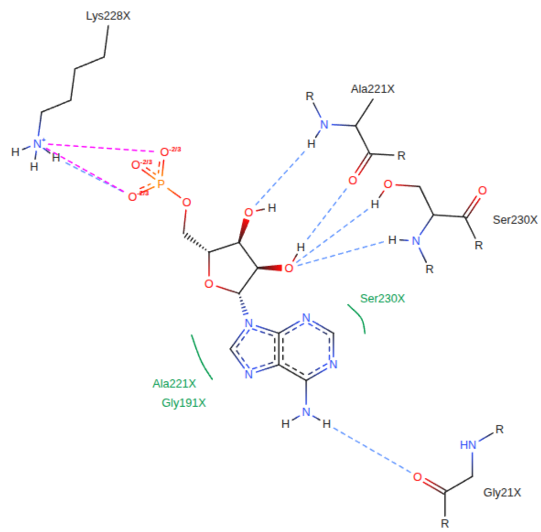

900 ns

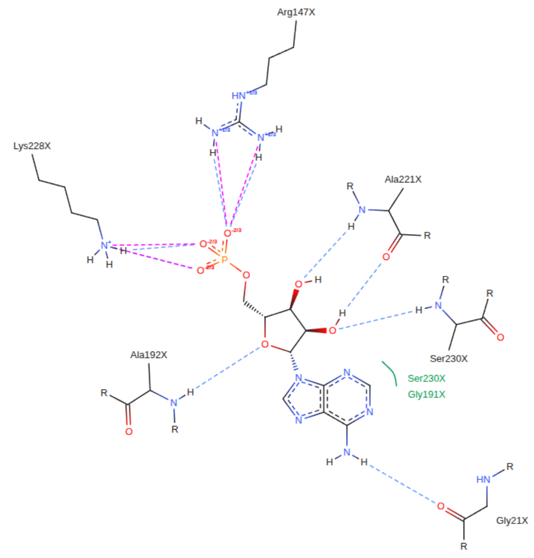

B. AMP at Glu crystal site

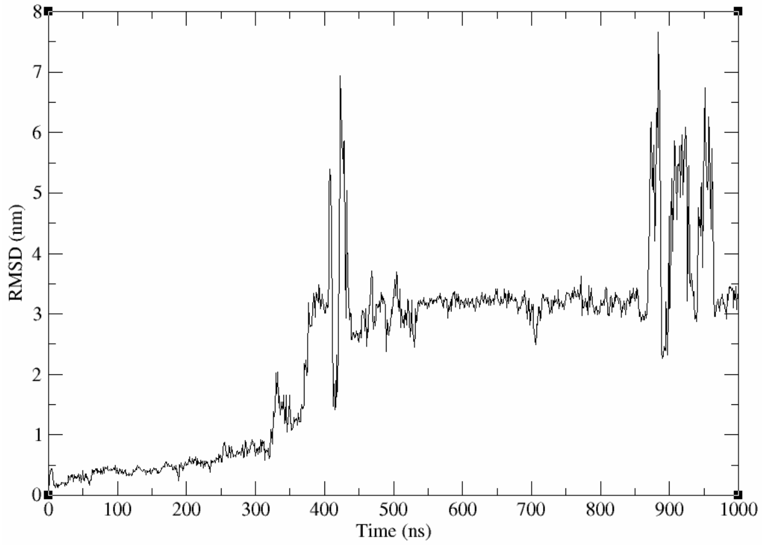

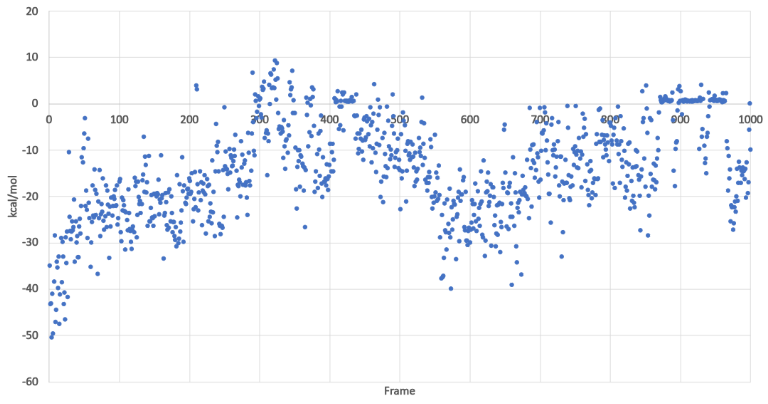

AMP at Glu crystal site

Docked 600 ns 800 ns

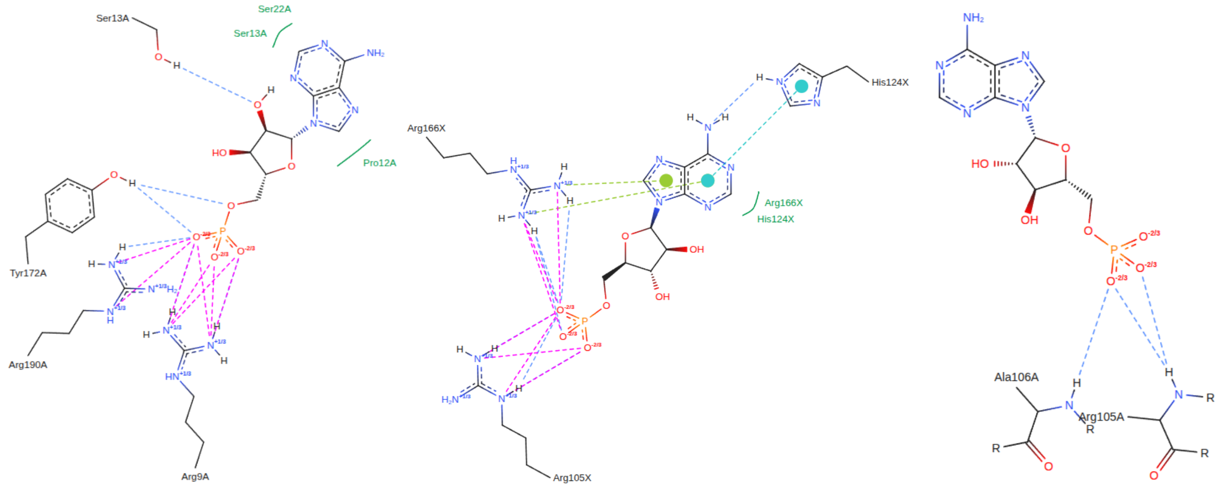

C. AMP at novel site

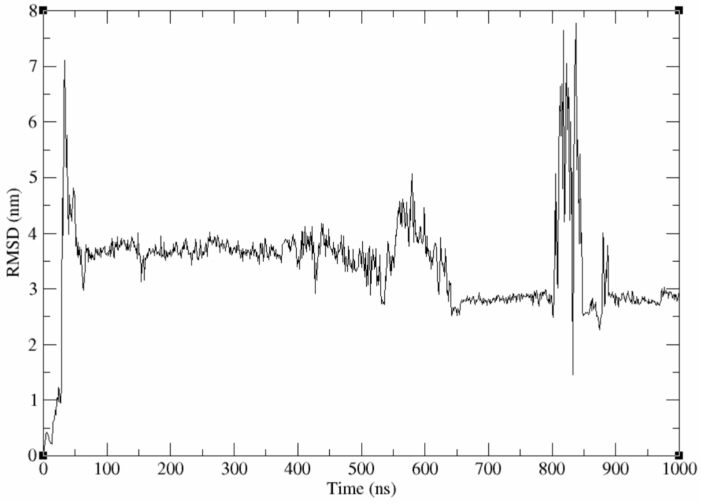

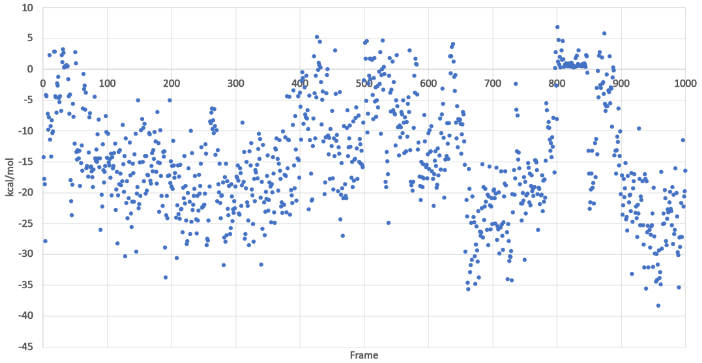

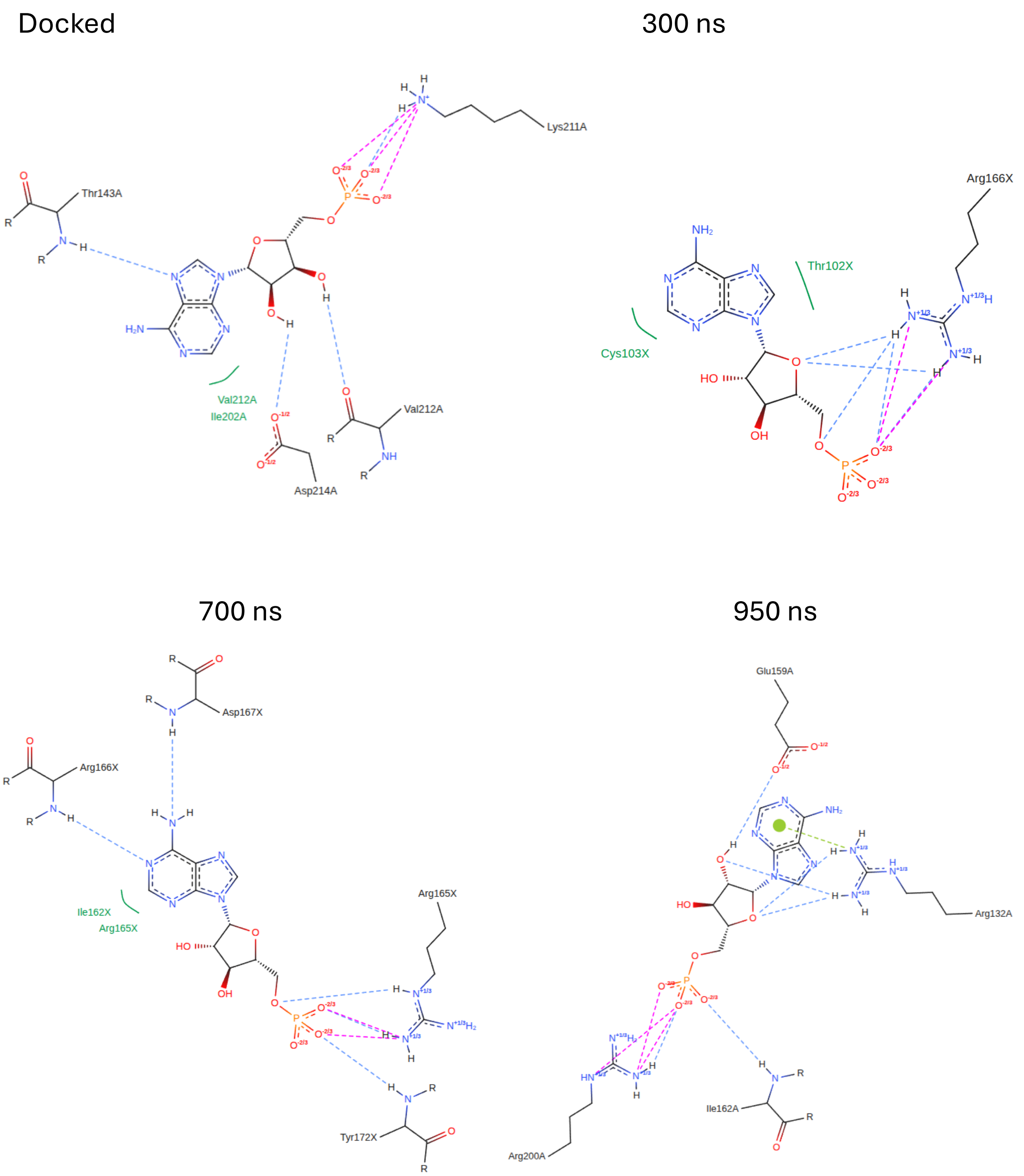

Docked
300 ns
700 ns
950 ns
Lys211A
Thr143A
Val212A
Ile202A
Val212A
Asp214A
Arg166X
Thr102X
Cys103X
Asp167X
Arg166X
Ile162X
Arg165X
Arg165X
Tyr172X
Glu159A
Arg132A
Ile162A
Arg200A
