# Supplementary figures and images for: An MD View of Ligand Binding
Source: Molecules. 2025 Dec 6;30(24):4678. doi: 10.3390/molecules30244678 (PMC12736043; doi:10.3390/molecules30244678)

## Queuosine

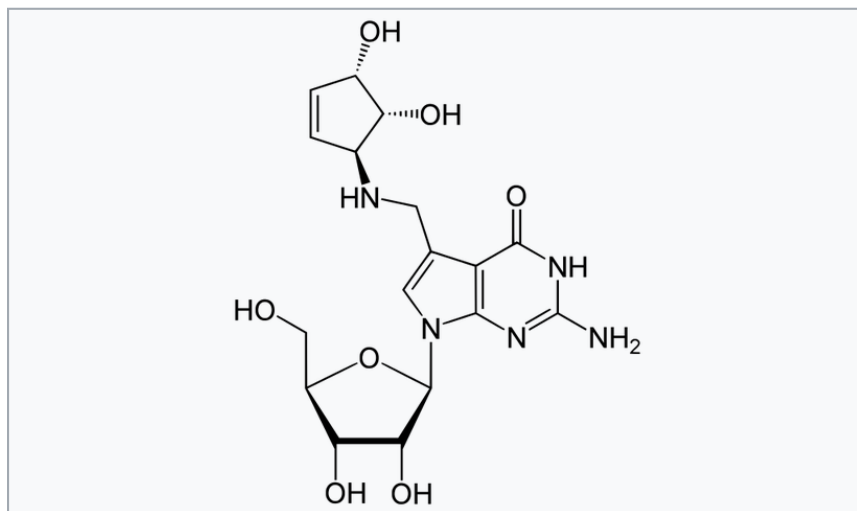

## Glutamyl-AMP

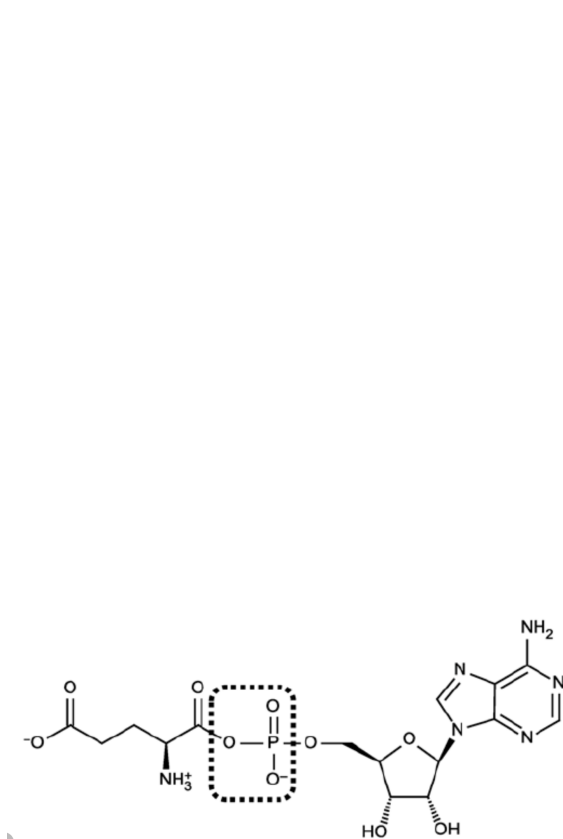

## Glutamol-AMP

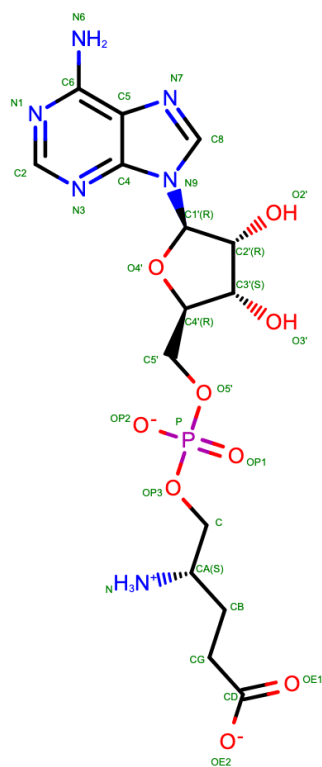

Supplement: Supplementary file 1 [file molecules-30-04678-s001.zip › Supplemental Figure S1 Queuosine GluAMP GloAMP structures.pdf]

### Supplemental Figure S7 PoseEdit views SAM and cAMP

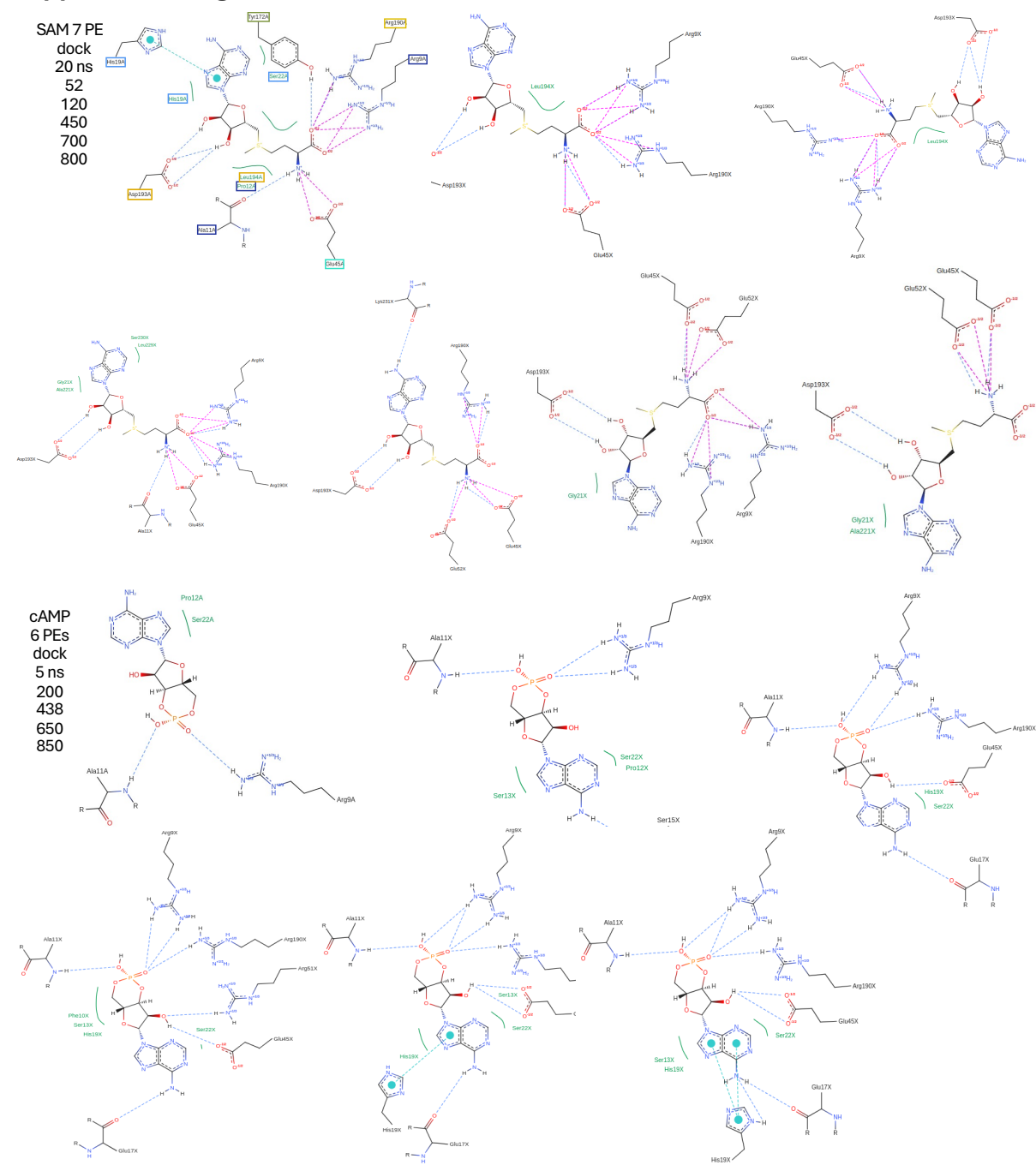

Supplement: Supplementary file 1 [file molecules-30-04678-s001.zip › Supplemental Figure S7 PoseEdit views SAM and cAMP.pdf]
